# Systematic Modification of Zingerone Reveals Structural Requirements for Attraction of Jarvis’s Fruit Fly

**DOI:** 10.1038/s41598-019-55778-4

**Published:** 2019-12-18

**Authors:** Benjamin L. Hanssen, Soo Jean Park, Jane E. Royer, Joanne F. Jamie, Phillip W. Taylor, Ian M. Jamie

**Affiliations:** 1 Department of Molecular Sciences, Macquarie University, North Ryde, NSW 2109 Australia; 2 Department of Agriculture and Fisheries, PO Box 267, Brisbane, Qld 4000 Australia; 3 Department of Biological Sciences, Macquarie University, North Ryde, NSW 2109 Australia

**Keywords:** Chemical biology, Environmental chemistry, Organic chemistry, Chemical synthesis

## Abstract

Tephritid fruit flies are amongst the most significant horticultural pests globally and male chemical lures are important for monitoring and control. Zingerone has emerged as a unique male fruit fly lure that can attract dacine fruit flies that are weakly or non-responsive to methyl eugenol and cuelure. However, the key features of zingerone that mediate this attraction are unknown. As Jarvis’s fruit fly, *Bactrocera jarvisi* (Tryon), is strongly attracted to zingerone, we evaluated the response of *B. jarvisi* to 37 zingerone analogues in a series of field trials to elucidate the functional groups involved in attraction. The most attractive analogues were alkoxy derivatives, with isopropoxy being the most attractive, followed by ethoxy and trifluoromethoxy analogues. All of the phenolic esters tested were also attractive with the response typically decreasing with increasing size of the ester. Results indicate that the carbonyl group, methoxy group, and phenol of zingerone are key sites for the attraction of *B. jarvisi* and identify some constraints on the range of structural modifications that can be made to zingerone without compromising attraction. These findings are important for future work in developing and optimising novel male chemical lures for fruit flies.

## Introduction

Males of many dacine fruit flies (*Bactrocera* Macquart, *Zeugodacus* Hendel, and *Dacus* Fabricius) are attracted to specific secondary metabolites produced by some plants and to analogues of these compounds^1,2^. The reason for the attraction of male fruit flies to these compounds, commonly referred to as male lures, attractants, or parapheromones, is generally considered to be related to mating success^1,3^. When males feed on these natural lures, the ingested lure compound is transported and stored in the rectal gland intact or modified, before it is released together with pheromones during sexual advertisement^4–7^. Lure consumption may also improve male competitiveness and mating success by increasing energy metabolism^8^ and allowing lure-fed males to attract females at an earlier time^9^, and can be used in sterile insect technique management programmes to accelerate sexual maturation^10^. The effects of lure consumption by males varies between species^11^ and male lure^12^. The strong attraction of male fruit flies to lures has allowed these compounds to be used for fruit fly population monitoring and control. Traps containing male lures are routinely used to estimate population sizes, to demonstrate pest-free status, or to detect incursions of invasive species^13,14^. Male lures are also used extensively in the male annihilation technique in which a lure is combined with a toxicant to kill large numbers of male fruit flies such that female fertility is reduced^15^. Lures with improved attractiveness to fruit flies enable more effective management and monitoring.

**Figure 1:**
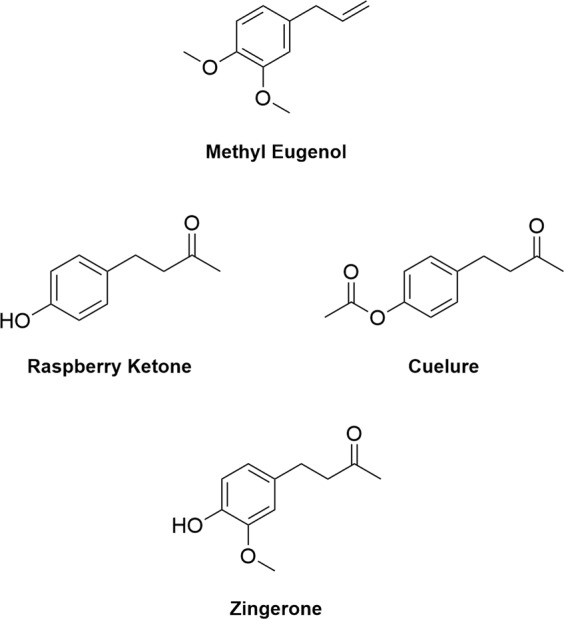
Chemical structures of the male chemical lures methyl eugenol, raspberry ketone, cuelure, and zingerone.

Many dacine fruit flies respond to either of two male lures: methyl eugenol (4-allyl-1,2-dimethoxybenzene) or cuelure (4-(4-acetoxyphenyl)-2-butanone) (Fig. 1)^16^. Cuelure is the acetyl ester of raspberry ketone (4-(4-hydroxyphenyl)-2-butanone) (Fig. 1), a far more common compound in nature that is also attractive, but less so than cuelure probably due to its lower volatility and release rate^17,18^. *Bactrocera*, *Zeugodacus*, and *Dacus* species are typically categorised as being methyl eugenol responsive, cuelure/raspberry ketone responsive, or non-responsive^19,20^. However, around 50% of species do not respond to either methyl eugenol or cuelure, or have never been trapped using these lures^16,20,21^.

Zingerone (4-(4-hydroxy-3-methoxyphenyl)-2-butanone) (Fig. 1) is a compound that illustrates the complexity of fruit fly lure attraction. The flowers of two *Bulbophyllum* orchids (*Bu. patens* King and *Bu. baileyi* F. Muell.) have been observed to attract males of some fruit flies, including the methyl eugenol responsive *B. carambolae* Drew & Hancock and *B. dorsalis* (Hendel), and the cuelure/raspberry ketone responsive *B. albistrigata* (de Meijere), *Z. caudatus* (Fabricius), *Z. cucurbitae* (Coquillett), and *Z. tau* (Walker)^22,23^. Laboratory and field trials showed that zingerone was the active component in the flower volatiles^22,23^. A mixture of methyl eugenol responsive and cuelure/raspberry ketone responsive fruit flies were attracted by zingerone, which makes zingerone unique among male lures as no species is attracted to both methyl eugenol and cuelure/raspberry ketone^19^.

While zingerone can attract both methyl eugenol responsive and cuelure/raspberry ketone responsive species, zingerone is a much weaker lure for *B. dorsalis* and *Z. cucurbitae* than methyl eugenol or cuelure, respectively^11,22,23^. Field trials with zingerone and a small number of zingerone analogues have revealed that some other fruit fly species are strongly attracted to zingerone, including Jarvis’s fruit fly, *B. jarvisi* (Tryon)^24–28^, which has an olfactory threshold to zingerone of 179 ng (effective median dose, ED_50_)^29^. Previously considered to be a non-responsive species or only weakly attracted to cuelure^16,24,27^, the discovery of the strong attraction of *B. jarvisi* to zingerone represents the first effective lure for this species. *Bactrocera jarvisi* is a moderate pest species, with mangoes being the most susceptible commercial crop^30,31^, and is distributed through northern Australia, from Broome to eastern Arnhem Land and along the east coast of Australia from Cape York to Sydney^16^. While zingerone is structurally similar to both methyl eugenol and raspberry ketone (Fig. 1), the particular structural features of zingerone that are responsible for attraction of *B. jarvisi* and other responsive fruit flies are poorly understood.

Experiments using lure analogues can provide instructive insights into the structural, chemical, and physical properties that are required for attraction, and can help to identify compounds that are more attractive than existing lures. Attraction to lures is mediated by protein receptors in the antennae, or other olfactory organs, and since the structure of these receptors has not yet been elucidated in *B. jarvisi*, the testing of analogues provides valuable information regarding the lure binding site in these receptors.

Many analogues of methyl eugenol and cuelure have been evaluated for their attraction of *B. dorsalis* and *Z. cucurbitae*, respectively. Patterns in the attraction of analogues were used to elucidate the key structural features of methyl eugenol and cuelure and sites of modification in laboratory bioassays^32–35^. Research into methyl eugenol analogues has also been concerned with developing analogues that eliminate or minimise the suspected carcinogenicity potential of methyl eugenol^36^, with side chain fluorinated analogues being the most effective compounds tested^37–40^. While chemical structure has been found to be more important for attraction than vapour pressure in some studies^18,35^, vapour pressure is considered important for effective lure attraction^41^, and efforts to increase the vapour pressure of cuelure have focused on developing analogues with phenolic ester, fluorinated, and silyl ether modifications^18,42,43^.

Unlike methyl eugenol and cuelure, few analogues of zingerone have been evaluated in the field for attraction of fruit flies. In the present study, zingerone analogues were synthesised and evaluated in the field to elucidate the functional groups that mediate attraction of *B. jarvisi* and to probe for sites of potential structural modification of zingerone. *Bactrocera jarvisi* was chosen as a model species due to its strong response to zingerone, but the insights of this study also provide important guidance for the development of effective lures for other species. Additionally, some analogues that were expected to have higher vapour pressures were included, including some fluorinated compounds. To evaluate the effect of structural modifications on vapour pressure, differential scanning calorimetry (DSC) was used to measure the vapour pressure of zingerone and a subset of zingerone analogues that were expected to have higher vapour pressures and hence higher release rates.

## Results

### Field trials

**Figure 2:**
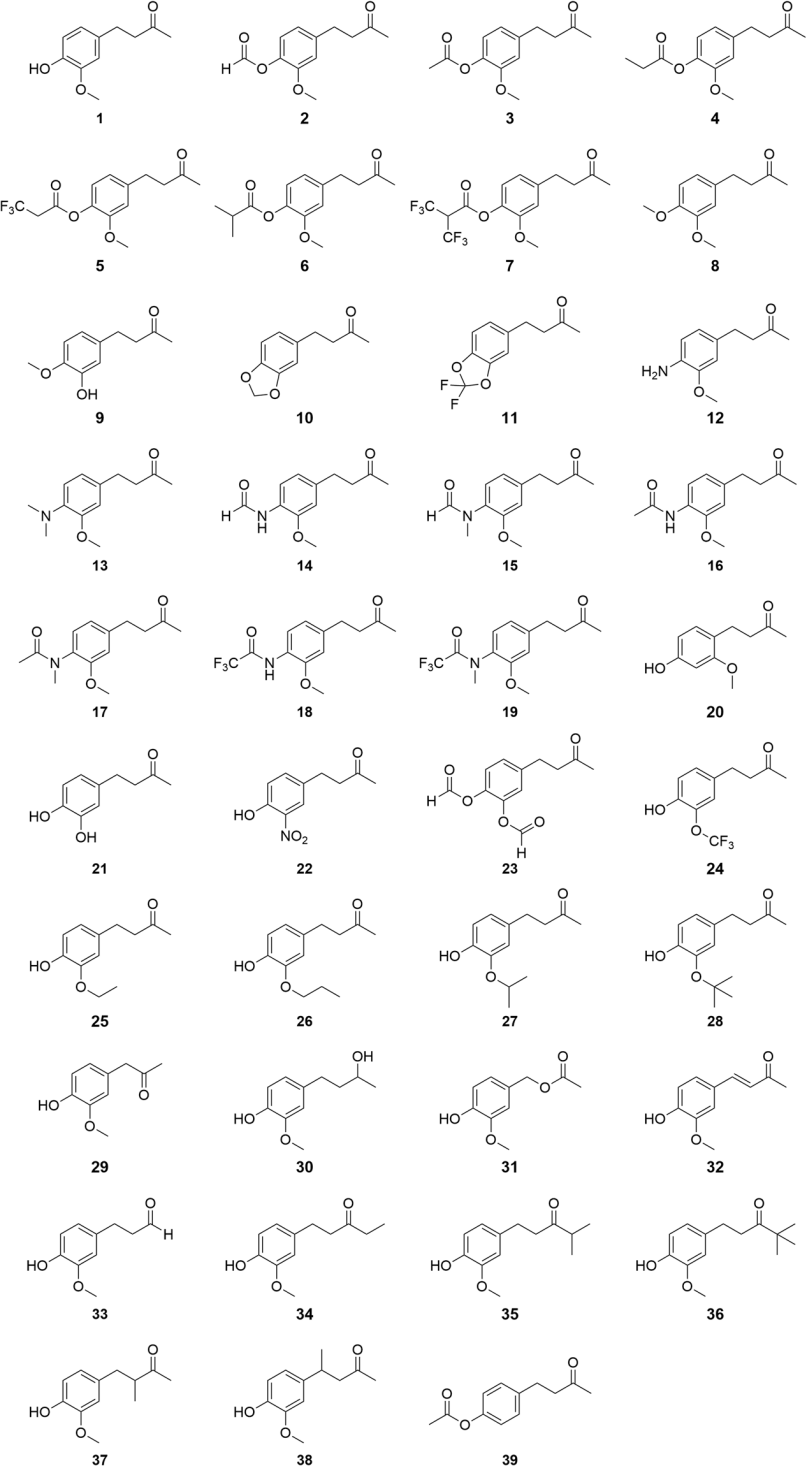
Chemical structures of the compounds investigated: zingerone (**1**), zingerone analogues **2**–**38**, and cuelure (**39**).

The zingerone analogues **2–38** together with zingerone (**1**) and cuelure (**39**) (Fig. 2) were tested over three periods during January and February across 2017–2019. The amount of compound used in each trap (300 mg) was between the minimum amount required for short range attraction^29^ and the amount used in longer term field studies^24,26,27^. The blank control traps did not capture any flies. *Bactrocera jarvisi* represented 99.0–99.9% of flies collected in zingerone or zingerone analogue traps. Other species collected at zingerone or zingerone analogue traps were predominately *B. tryoni* (Froggatt) with smaller numbers of *B. neohumeralis* (Hardy) as well as two specimens of *B. breviaculeus* (Hardy) and a single *D. secamoneae* Drew. A similar proportion of *B. tryoni* in zingerone traps was found by previous studies^24,27^. Cuelure (**39**) traps caught between seven and nine species each season, primarily *B. tryoni* (68.3–83.4%), with smaller numbers of *B. neohumeralis*, *B. breviaculeus*, *B. aeroginosa* (Drew & Hancock), *B. alyxiae* (May), *B. bryoniae* (Tryon), *B. quadrata* (May), *B. peninsularis* (Drew & Hancock), *B. rufofuscula* (Drew & Hancock), *B. silvicola* (May), *Z. choristus* (May), and *Z. cucumis* (French) (a single female), which have all been previously recorded in Australia at cuelure traps^16^. Three male *B. jarvisi* were caught by cuelure (**39**) traps during the 2019 field trial.

**Figure 3:**
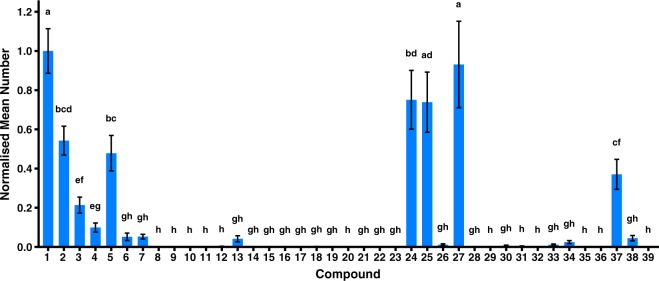
Number of *B. jarvisi* caught by each compound normalised to zingerone (**1**) (mean ± SE) of the respective year for the zingerone analogues **2**–**38** and cuelure (**39**). Means with a common letter are not significantly different (*p* > 0.05). Statistical analysis was performed on square root transformed values.

To allow for comparison across three years of trials, the number of *B. jarvisi* caught per trap per day was normalised to the value for zingerone (**1**) in the respective year. This controls for different abundances of *B. jarvisi* from year to year. The normalised field catches for the 37 zingerone analogues and cuelure (**39**) are presented in Fig. 3 and the absolute number of *B. jarvisi* caught per trap per day in each year is shown in Supplementary Table S1. Zingerone (**1**), the phenolic esters **2**–**3** and **5**, the methoxy derivatives **24**–**25** and **27**, and the 3-methyl-2-butanone analogue **37** were the most attractive compounds (Fig. 3). While the phenolic esters (**2**–**7**) were less attractive than zingerone, all of the phenolic esters were attractive. Despite having the same ester chain length as **4**, the 3,3,3- trifluoropropionyl ester **5** was significantly more attractive than **4** (*p* < 0.001) and was as attractive as the smaller formyl ester **2**. Several nitrogen-containing analogues were tested including an aniline **12**, an *N,N*-dimethylaniline **13**, and the amides (**14**–**19**); of which, only the *N,N*-dimethylaniline **13** was weakly attractive. The attraction of the methoxy derivatives (**20**–**28**) was clearly divided between unattractive and highly attractive compounds. The only attractive methoxy derivatives were the trifluoromethoxy **24**, ethoxy **25**, and isopropoxy **27** analogues. Methylation of the terminal end of the butanone chain resulted in a rapid reduction in attraction with the 3-pentanone analogue **34** being weakly attractive and the 2-methyl-3-pentanone **35** and 2,2-dimethyl-3-pentanone **36** analogues being unattractive. Methylation on the other side of the ketone, the benzene ring side of the ketone (**37**), was significantly more tolerated than terminal methylation as in the 3-pentanone **34** analogue (*p* < 0.001). There was also a difference in the attraction of the two analogues that had methyl groups added between the ketone and benzene ring, with the 3-methyl-2-butanone analogue **37** being significantly more attractive than the 4-methyl-2-butanone analogue **38** (*p* < 0.001).

### Compound retention and loss

**Figure 4:**
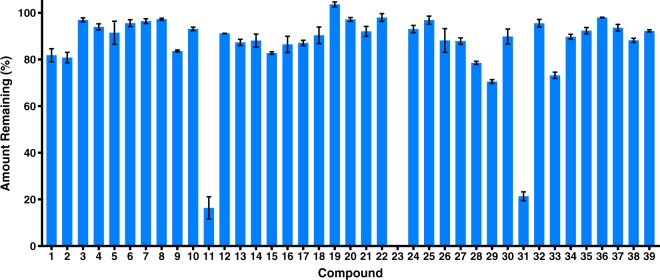
Relative amount of each compound (mean ± SE) remaining on the wicks at the end of field trials.

The wicks from field trials were collected and analysed to determine the amount of each compound remaining and if any chemical change had occurred, such as hydrolysis. Figure 4 shows the relative amount of each compound remaining from the three field trials.

After the field trials most wicks still had 80–100% of the initial amount of compound remaining. The diformyl ester **23**, however, had completely hydrolysed to the diphenol analogue **21** by the end of the field trial. The benzyl acetate analogue **31** had only 20% of the initial amount of material remaining on the wicks together with approximately 5% of the hydrolysis product, 4-hydroxy-3-methoxybenzyl alcohol. The formyl ester **2** was the only other ester that experienced significant hydrolysis, with the amount of the hydrolysis product, zingerone, increasing from an initial amount of 5% to approximately 12% at the end of the field trial. The difluoromethylenedioxy analogue **11** experienced a large loss of compound during the field trials with only 16% remaining on average across the three sites. The propanone **29** and propionaldehyde **33** analogues also had a greater loss of material than most of the other compounds. Except for the hydrolysis products of the esters **2**, **23**, and **31**, no other chemical transformation products were detected by GC-FID. While evaporation and hydrolysis were the main expected routes of compound loss, loss of the compounds may have also occurred through consumption of the compounds by flies^32,33,35^ and through degradation into products not detected by this method. However, the high recovery of most compounds indicates that any loss through consumption or degradation was minimal. Additionally, the greater loss of some compounds could generally be attributed to hydrolysis or relatively high vapour pressure.

### Vapour pressure

The boiling points of some of the analogues expected to have greater vapour pressures (**2**–**3**, **8**, **10**-**11**, and **24**), as well as zingerone, were measured using DSC under different applied reduced pressures to produce a set of temperature-pressure data for each compound, which are shown in Supplementary Table S2. These temperature-pressure data were used to obtain the Antoine Equation parameters *A*, *B*, and *C*, which are presented in Supplementary Table S3 together with the validity range of the fitted curve. Extrapolating the fitted Antoine Equations, the vapour pressure and volatility of each compound was determined at 298.15 K (25 °C) and is summarised in Table 1. The two phenolic esters examined, the formyl ester **2** and acetyl ester **3**, had a vapour pressure approximately half that of zingerone (**1**). Methylation of the phenol of zingerone to give methylzingerone (**8**) resulted in a 2.5-fold increase in vapour pressure compared to zingerone. The methylenedioxy analogues (**10** and **11**) had much higher vapour pressures than zingerone with the vapour pressure of the fluorinated methylenedioxy analogue **11** being 7-fold greater than the non-fluorinated methylenedioxy analogue **10**. The trifluoromethoxy analogue **24** also had a 7-fold greater vapour pressure than the non-fluorinated zingerone.

**Table 1:** Vapour pressure, volatility, and relative vapour pressure extrapolated to 298.15 K using the Antoine Equation for compounds **1**–**3**, **8**, **10**–**11**, and **24**.

Compound	Vapour Pressure at 298.15 K (Pa)	Volatility at 298.15 K (mg m^−3^)	Relative Vapour Pressure
1	0.011	0.87	1.0
2	0.0054	0.49	0.49
3	0.0057	0.54	0.51
8	0.028	2.4	2.5
10	0.10	7.8	9.0
11	0.73	67	66
24	0.078	7.8	7.0

## Discussion

**Figure 5:**
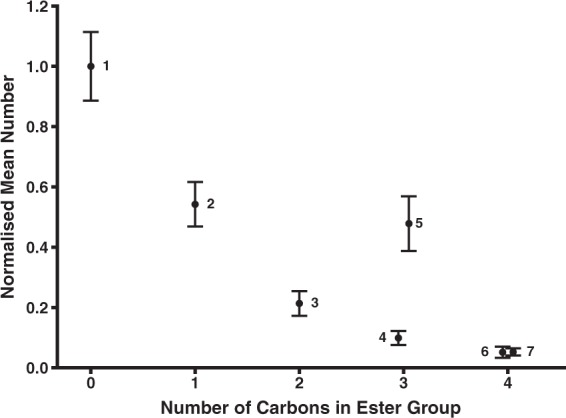
Number of *B. jarvisi* caught by zingerone (**1**) and the phenolic esters (**2**–**7**) normalised to zingerone (mean ± SE) against the number of carbons in the ester functional group. Numbers beside data points indicate the compound number.

This study identifies elements of the zingerone structure that are critical for attraction of *B. jarvisi* and elucidates some steric and electronic requirements of the receptor binding site. Many of the attractive compounds are phenolic esters (**2**–**7**) of zingerone. It was expected that the esters would be attractive because esters of raspberry ketone are attractive to raspberry ketone responsive flies with some being even more attractive than raspberry ketone, such as cuelure and melolure (4-(4-formoxyphenyl)-2-butanone)^41^. Unlike the esters of raspberry ketone, the esters of zingerone are not more attractive than the parent compound. This opposite trend could be due to the lower vapour pressure of the zingerone esters compared to zingerone (Table 1). Conversely, raspberry ketone esters have a higher vapour pressure than raspberry ketone^18^, which should increase the release rate and atmospheric concentration of the esters emitted from traps, and thereby attraction^41^. For the non-fluorinated phenolic esters, the order of the attractiveness to *B. jarvisi* (**6** ≤ **4** < **3** < **2**) is opposite to the size of the ester group (formyl **2** < acetyl **3** < propionyl **4** < isobutyryl **6**) (Fig. 5). An inverse relationship has been proposed for raspberry ketone and its esters^41^. Vapour pressure may be partially responsible for this relationship as a larger ester group will tend to decrease the vapour pressure of the compound. While melolure, which has a small formyl group, is more attractive than cuelure to some species, it is not universally more attractive to all cuelure responsive species than cuelure, which has a larger acetyl group^24,42^. The ability of the esters to fit in the binding site of the receptor may also explain this relationship, with smaller esters being more easily accommodated in the binding site. However, the attractiveness of the two fluorinated phenolic esters is contradictory. The 3,3,3-trifluoropropionyl ester **5**, which was as attractive as the formyl ester **2**, suggests that if the ester is not hydrolysed before reaching the receptor, the binding site can accommodate larger ester groups. This supports the vapour pressure explanation as the presence of fluorine could have significantly increased the vapour pressure of **5**. Despite also being fluorinated, the hexafluoroisobutyryl ester **7** was only as attractive as its non-fluorinated equivalent, the isobutyryl ester **6**, which may suggest that the vapour pressure of **7** is not appreciably greater than **6**.

Other modifications to the phenol of zingerone, including alkylation and replacement of the OH group with the isosteric NH_2_, resulted in much weaker attraction than with the phenolic esters. Simple methylation, as in methylzingerone (**8**), clearly demonstrates the loss of attraction with alkylation. Isozingerone (**9**) and the methylenedioxy analogues (**10** and **11**) are complicated by additional modification of the *meta* methoxy group as well as alkylation of the phenol. Alkylation removes the ability of the compounds to interact through hydrogen bond donation. The lack of attraction of these ether compounds could indicate that zingerone and similar phenols are acting as hydrogen bond donors. However, the phenolic esters cannot act as hydrogen bond donors yet are still attractive compounds. Hydrolysis of the phenolic esters to zingerone by esterases present in the sensilla lymph^44,45^ prior to receptor detection could explain the difference in attraction between the phenolic esters and the ethers. The methylenedioxy analogues (**10** and **11**) also show that despite possessing relatively high vapour pressures (Table 1) and a high rate of evaporation for **11** (Fig. 4), the chemical structure tends to be the dominant factor in determining attractiveness. The anilines (**12** and **13**) and amides (**14**–**19**), which are isosteric with zingerone (**1**), methylzingerone (**8**), and the phenolic esters (**2**–**7**) respectively, were unattractive except for the weakly attractive *N,N*-dimethylaniline **13**. This may have been due to the weaker hydrogen bonding properties or suboptimal structural conformations compared to zingerone and the phenolic esters. While methylation of zingerone to methylzingerone (**8**) eliminated attraction, methylation of the aniline **12** to *N,N*-dimethylaniline **13** increased attraction.

The strength of attraction of *B. jarvisi* to the analogues was very sensitive to changes to the methoxy group of zingerone. Moving the methoxy group *ortho* to the butanone chain, as in **20**, eliminated attraction, which indicates that the methoxy group is involved in some critical interaction with the receptor. The very weak attractiveness or unattractiveness of raspberry ketone and analogues, such as cuelure (**39**)^26,27,29^, which lack the *meta* methoxy group, further demonstrate the importance of a methoxy group in that position for *B. jarvisi* attraction. Replacing the methoxy group with functional groups other than alkoxys, such as phenol (**21**), nitro (**22**), and formoxy (**23**), also yielded unattractive analogues. The trifluoromethoxy analogue **24** is the most structurally similar to zingerone with fluorine often considered to approximate the size of hydrogen^46^. Despite this high structural similarity and the addition of fluorine, which increased the vapour pressure compared to zingerone (Table 1), the replacement of the methoxy group with a trifluoromethoxy group had an overall negative effect on attraction. The presence of the trifluoromethoxy group alters the electronic properties of the compound and the inductive electron-withdrawing effect of the trifluoromethoxy group might be responsible for reduced attractiveness. Analogues with larger alkoxy groups were investigated and provided some insight into the steric constraints of the receptor binding site. The ethoxy analogue **25** was as attractive as zingerone (**1**) but an increase in the alkoxy chain length to a propoxy group (**26**) virtually eliminated attraction with **26** catching only approximately 1% of the number of *B. jarvisi* caught by zingerone. The isopropoxy analogue **27** was also a three-carbon alkoxy group but is branched as opposed to the linear chain of **26**, and like the ethoxy analogue **25**, the isopropoxy analogue **27** was as attractive as zingerone. Increasing the bulkiness of the alkoxy group to a *tert*-butoxy group (**28**) resulted in an unattractive analogue. The field attraction of **25**–**28** suggests that the receptor binding site can accommodate compounds with an alkoxy group of 1–2 carbons in length and with some bulkiness but less than that of a *tert*-butoxy.

The lack of attraction of *B. jarvisi* to the butanone derivatives **29**–**32** highlights the importance of the carbonyl group for attraction. The carbonyl group has been moved closer to the benzene ring in the propanone analogue **29** and reduced to an alcohol in zingerol (**30**), which demonstrate that the presence and position of the carbonyl group is critical. Chemical analysis of the wicks after field trials indicated that there was some hydrolysis of the benzyl acetate analogue **31**, which may be the reason for its lack of attraction of *B. jarvisi*. The hydrolysis product of **31**, 4-hydroxy-3-methoxybenzyl alcohol, does not possess the carbonyl group necessary for effective attraction. The lack of *B. jarvisi* attraction by dehydrozingerone (**32**) may be due to the alkene preventing the compound from assuming a particular conformation that allows the carbonyl group to correctly interact with the receptor binding site.

The effect of increasing the steric bulk around the butanone chain on attraction was explored by adding methyl groups to the butanone chain. Placing an additional methyl group on the terminal end of the butanone chain (**34**) substantially reduced the attractiveness to *B. jarvisi*, which suggests that the receptor binding site cannot accommodate larger functional groups at the end of the butanone chain. Indeed, increasing the bulkiness of the end of the butanone chain further with 2-methyl-3-pentanone (**35**) and 2,2-dimethyl-3-pentanone (**36**) chains completely eliminated attraction. With a limited ability to accommodate bulkiness, it is interesting that the propionaldehyde analogue **33** is also unattractive to *B. jarvisi*. This may be due to this analogue possessing an aldehyde rather than a ketone, which confers greater reactivity to **33**, or the terminal methyl group of the butanone chain may be critical for receptor recognition. Since an additional methyl group was not tolerated on the terminal side of the ketone, it was expected that the 3-methyl-2-butanone analogue **37** would be unattractive, yet **37** was moderately attractive while the 4-methyl-2-butanone analogue **38** was unattractive. This suggests that the receptor binding site can accommodate somewhat more steric bulk on the benzene ring side of the ketone than the terminal methyl side. The unattractiveness of the 4-methyl-2-butanone analogue **38** may be due to an inability to sterically accommodate the additional methyl group or it interferes with recognition of the benzene ring.

In summary, we report on the response of *B. jarvisi* to a series of analogues of zingerone using field trials. Field trials of the compounds found that the most attractive analogues were the formyl **2** and 3,3,3-trifluoropropionyl **5** phenolic esters; the trifluoromethoxy **24**, ethoxy **25**, and isopropoxy **27** alkoxy analogues; and the 3-methyl-2-butanone analogue **37**, though none were more attractive than zingerone. For zingerone and the analogues with measured vapour pressures, there was little correlation between vapour pressure and field attractiveness to *B. jarvisi*. While all of the phenolic esters tested were somewhat attractive, the response decreased with increasing size of the ester, except for the fluorinated ester 3,3,3-trifluoropropionyl **5**, which was as attractive as the formyl ester **2**. The attraction of *B. jarvisi* to the alkoxy analogues suggests that the receptor can only accommodate alkoxy groups of 1–2 carbons in length and the bulkiness of an isopropoxy but not that of a *tert*-butoxy. Greater steric bulk around the butanone chain generally resulted in unattractive analogues with only methylation on the benzene ring side of the ketone being moderately attractive. These results demonstrate that the carbonyl group, phenol, and methoxy group are key sites for the attraction of *B. jarvisi* and identify some constraints on the range of structural modifications that can be made to zingerone without compromising attraction.

## Methods and Materials

### Reagents and synthesis of zingerone analogues

The compounds used in this study are presented in Fig. 2. Zingerone (**1**) ( ≥ 96% purity), the propanone analogue **29** (96%), dehydrozingerone (**32**) ( ≥ 98.5%), and cuelure (**39**) ( ≥ 96%) were purchased from Sigma-Aldrich and used without further purification. The esters **2**, **5**, and **7** were synthesised from zingerone and the appropriate carboxylic acid by the Steglich esterification^47^. The formyl ester **2** sample still contained approximately 5% zingerone by GC-FID and ^1^H-NMR after purification. The esters **3**, **4**, and **6** were synthesised from zingerone and the appropriate acid anhydride with pyridine as a catalyst^48^. Analogues **8**–**11**, **20**, and **24** were synthesised in two steps from the appropriate substituted benzaldehyde by an aldol condensation^49^ and rhodium on alumina catalysed hydrogenation^50^ of the subsequent enone. The benzaldehyde for the synthesis of **10** (piperonal) was synthesised from piperonyl alcohol using manganese(IV) oxide^51^. The aniline **12** was also synthesised from an aldol condensation of 4-nitro-3-methoxybenzaldehyde and acetone (optimised from Wang*, et al*.^52^) followed by a rhodium on carbon hydrogenation^50^ of the enone and nitro group. The *N,N*-dimethylaniline **13** was synthesised from the aniline **12** by a formaldehyde reductive amination^53^. The formamide **14** was synthesised from **12** using formic acetic anhydride^54^. The acetamide **16** and trifluoroacetamide **18** were synthesised from **12** and the appropriate acid anhydride^55^. The *N*-methylamides **15**, **17**, and **19** were synthesised by *N*-methylation of the appropriate secondary amide with methyl iodide but after purification still contained approximately 15%, 12%, and 5% impurities, respectively, by GC-FID and ^1^H-NMR^56^. The nitrophenol analogue **22** was synthesised by aromatic nitration of raspberry ketone using nitric acid^57^. The diformyl ester **23** was synthesised from 4-(4-benzyloxy-3-hydroxyphenyl)-2-butanone using formic acetic anhydride followed by deprotection with palladium on carbon^58^ and further reaction with formic acetic anhydride^54^. The starting material for **23** was synthesised by benzyl protecting 3,4-dihydroxybenzaldehyde^59^ and reacting this with acetone in an aldol condensation (optimised from Wang*, et al*.^52^) followed by rhodium on carbon hydrogenation^50^. Analogues **21**, **25**, and **34**–**36** were synthesised from the appropriate benzyl protected benzaldehyde^60^ by an aldol condensation (optimised from Wang*, et al*.^52^ for **21**, **25**, and **34** and modified from Plourde^58^ for **35**–**36**) and rhodium on alumina catalysed hydrogenation^50^ of the enone followed by deprotection using palladium on carbon^58^. Analogues **26** and **27** were synthesised by alkylating 4-(4-benzyloxy-3-hydroxyphenyl)-2-butanone with an appropriate alkyl halide^61^ followed by deprotection using palladium on carbon^58^. The *tert*-butoxy analogue **28** was synthesised by alkylating 4-(4-benzyloxy-3-hydroxyphenyl)-2-butanone with di-*tert*-butyl dicarbonate and scandium(III) triflate as a catalyst^62^ followed by deprotection using palladium on carbon^58^. Zingerol (**30**) was synthesised from the sodium borohydride reduction of zingerone^63^. The benzyl acetate analogue **31** was synthesised by selective acetylation of 4-hydroxy-3-methoxybenzyl alcohol with acetic acid and potassium fluoride^64^. The propionaldehyde analogue **33** was synthesised in a multiple step procedure starting with the benzyl protection of vanillin^60^ followed by a Horner-Wadsworth-Emmons reaction using triethyl phosphonoacetate^65^ then hydrogenation with rhodium on alumina^50^, reduction of the ester with DIBAL^66^, and finally deprotection with palladium on carbon^58^. The 3-methyl-2-butanone analogue **37** was synthesised by benzyl protecting zingerone^60^ followed by α-methylenation using paraformaldehyde and piperidine^67^ then concurrent deprotection and hydrogenation using palladium on carbon^58^. The 4-methyl-2-butanone analogue **38** was synthesised from benzyl protected dehydrozingerone (**32**)^52,60^ followed by conjugate addition with methyllithium and copper(I) iodide^68^ then deprotection with palladium on carbon^58^. Detailed reaction procedures and spectra are presented in the supplementary information.

### Field trials

Field trials were undertaken at Walkamin Research Facility (17.13797°S, 145.41508°E), Malone Road (17.00298°S, 145.46742°E), and Mareeba Research Facility (17.00724°S, 145.42984°E), on the Atherton Tableland region of north Queensland between 10 January 2017 and 27 January 2017, 24 January 2018 and 3 February 2018, and 29 January 2019 and 17 February 2019. Compounds (300 ± 3 mg) were dissolved in acetone (or methanol for **32**) and applied to cotton dental wicks. Untreated wicks were used as blank controls (negative control). Once the solvent had evaporated, the wicks were placed in the centre of McPhail traps (BioTrap V1 (2017 field trial), BioTrap V2 (2018 field trial), and BioTrap V2 X (2019 field trial); BioTrap Australia Pty Ltd.) with a single dichlorvos cube (2,2-dichlorovinyl dimethyl phosphate, BioTrap Australia Pty Ltd.). Replicate sets of traps, comprising the compounds and blank control, were hung in shade under trees (Walkamin Research Facility: fruiting mango trees (2017), non-fruiting mango trees (2018), and a mixture of non-fruiting mango and peach trees (2019); Malone Road: fruiting mango trees (2017), non-fruiting mango trees (2018–2019); Mareeba Research Facility: non-crop trees (2017–2019)) approximately 1.5 m above the ground with a minimum of 10 m separation between traps. The traps were cleared daily and moved into the next trap position such that each trap only spent one day in each position. Flies were examined under a stereomicroscope and identified by referencing Drew^16^.

Field trial data were analysed using the software R^69^ and RStudio^70^ with the emmeans package^71^ and plotted with the ggplot2 package^72^. For analysis, a linear model was used in which the daily *B. jarvisi* catch was the response variable. The daily *B. jarvisi* catch values were $\sqrt{x+0.5}$ transformed before statistical analysis. The predictors in the model were compound and site and the interaction between these two variables was included. A pairwise comparison between each compound was performed with Tukey’s Honest Significant Difference test for multiple comparisons. The statistical analysis was repeated with the transformed daily *B. jarvisi* catch values normalised to the transformed value of zingerone (**1**) in the respective year to account for variations in the abundance of *B. jarvisi* year-to-year. Graphical analysis of the Pearson residuals was used to assess model assumptions.

### Compound retention and loss

At the conclusion of field trials, the wicks were immediately removed from the traps and stored in sealed vials for later analysis. Wicks were extracted with ethyl acetate for two hours at room temperature and tridecane was added to each wick as an internal standard. The extracts were diluted with ethyl acetate to an appropriate concentration for analysis by GC-FID. Quantification of the extracts was performed using a Shimadzu GC-17A gas chromatograph with flame ionisation detection and a Shimadzu AOC-20i autosampler. A Restek Rxi-5Sil MS 30 m column was employed, with the injector at 270 °C, the initial oven temperature at 100 °C for 1 min, then ramped at 15 °C min^−1^ to a final temperature of 250 °C and held for 1 min. Extracts were analysed in triplicate and the quantity of compound reported relative to the initial 300 mg amount.

Compound retention and loss data were analysed using the software R^69^ and RStudio^70^ with the ggplot2^72^ package. The relative quantity of material remaining on each wick was averaged across the three sites and three field trials (for zingerone (**1**) and cuelure (**39**)) to give a mean value for each compound. The value for zingerone at the Walkamin Research Facility site in the 2019 field trial was excluded as an outlier.

### Vapour pressure

Vapour pressure measurements were conducted on a TA Instruments 2010 DSC equipped with a standard DSC cell. Vacuum was achieved with a Vacuubrand MD4 diaphragm vacuum pump between 15 kPa and 0.5 kPa and an Edwards E2M-1.5 high vacuum pump between 0.5 kPa and 0.15 kPa. The pressure was regulated by a needle valve to balance inflow and outflow with the absolute pressure measured by an Edwards Active Pirani Gauge APG-L-NW16.

Calibration of the DSC was performed in accordance with ASTM E967-08. Temperature calibration was conducted with indium and lead. After temperature calibration of the DSC the pressure gauge was calibrated by measuring the boiling point of 1-octanol under different reduced pressures between atmospheric and 0.14 kPa and comparing the measured pressure to literature pressure-boiling data for 1-octanol^73^.

Samples of 8–14 mg of **1**–**3**, **8**, **10**–**11**, and **24** were weighed on a micro-analytical balance with a precision of ± 0.01 mg and placed in hermetic aluminium pans (TA Instruments) and sealed hermetically with hermetic pinholes lids (TA Instruments). Samples of the formyl ester **2** were of lower purity and contained approximately 5% zingerone. For pressures between atmospheric and 7 kPa, pinholes with a diameter of 75 μm were used. Use of pressures below 7 kPa required pinholes larger than 75 μm, which were prepared by manually punching the lids with a needle and the pinhole size was determined by microscopy. For pressures between 7 kPa and 1 kPa, pinhole diameters ranging from approximately 290 μm to 340 μm were used. Below 1 kPa, use of larger pinhole diameters ranging from approximately 460 μm to 540 μm was necessary. Due to reduced thermal contact between the pans and the sample and reference platforms of the DSC at reduced pressures, thermally conductive paste was applied to the pans and platforms for pressures < 7 kPa.

The operation of the DSC for vapour pressure measurements was performed in accordance with ASTM E1782-14 with a modified pressure range (15 kPa to 0.15 kPa). After achieving the desired pressure, the sample in the DSC was rapidly heated to approximately 50 °C below its expected boiling point and allowed to equilibrate. Once equilibrated, heating at 5 °C min^−1^ was initiated and maintained until a stable baseline was achieved after the sample boiled. The pressure was measured when the sample began to boil, and the temperature of the extrapolated onset point was determined from the DSC endotherm.

The pressure-boiling point data from the DSC measurements were fitted to the Antoine Eq. (1).

1 $$\log \,P=A-\frac{B}{T+C}$$

where *P* is pressure (kPa), *T* is temperature (K), and *A*, *B*, and *C* are the Antoine parameters. The parameters were obtained by iterative least squares nonlinear regression with MATLAB R2015a (The MathWorks, Inc.). The vapour pressure and volatility of the compounds at room temperature (298.15 K) were calculated using the Antoine Equation (Eq. (1)) and Eq. (2), respectively.

2 $$Volatility=\frac{PM}{RT}\times {10}^{6}$$

where *volatility* is given in mg m^−3^, *P* is vapour pressure (kPa), *M* is molar mass (g mol^−1^), *R* is the ideal gas constant (J K^−1^ mol^−1^), and *T* is temperature (K).

## Supplementary information

Supplementary information
