# Supplementary material for: Systematic Modification of Zingerone Reveals Structural Requirements for Attraction of Jarvis’s Fruit Fly
Source: Sci Rep. 2019 Dec 18;9:19332. doi: 10.1038/s41598-019-55778-4 (PMC6920482; doi:10.1038/s41598-019-55778-4)
Supplement: Supplementary file 1 — Supplementary information [file 41598_2019_55778_MOESM1_ESM.pdf]

Systematic Modification of Zingerone Reveals Structural Requirements for Attraction of Jarvis's Fruit Fly

Benjamin L. Hanssen[1], Soo Jean Park[1], Jane E. Royer[2], Joanne F. Jamie[1], Phillip W. Taylor[3], Ian M. Jamie[1]*

[1] Department of Molecular Sciences, Macquarie University, North Ryde, NSW 2109, Australia.

[2] Department of Agriculture and Fisheries, PO Box 267, Brisbane, Qld 4000, Australia.

[3] Department of Biological Sciences, Macquarie University, North Ryde, NSW 2109, Australia.

* Correspondence and requests for materials should be addressed to I.M.J. (email: ian.jamie@mq.edu.au)

Synthesis
General Procedure

^{1}H, ^{13}C, and ^{19}F nuclear magnetic resonance (NMR) spectra were recorded using a Bruker Avance DPX 400 or Bruker Ascend 400 NMR spectrometer operating at 400 MHz for ^{1}H NMR, 101 MHz for ^{13}C NMR, and 376 MHz for ^{19}F NMR. $CDCl_3$ was used as the solvent for all NMR samples. ^{1}H NMR chemical shifts are reported in parts per million (δ) referenced to the proton signal of the deuterated solvent ($CDCl_3$: 7.26 ppm). ^{13}C NMR chemical shifts are reported in parts per million (δ) referenced to the carbon signal of the deuterated solvent ($CDCl_3$: 77.16 ppm). ^{19}F NMR chemical shifts are reported in parts per million (δ) referenced to the fluorine signal of trifluoroacetic acid (-76.55 ppm). The following abbreviations are used to describe the NMR data – singlet (s), doublet (d), triplet (t), quartet (q), septet (sep), doublet of doublets (dd), multiplet (m), and broad (br). Low resolution mass spectra were recorded on a Shimadzu GC-2010 and Shimadzu GCMS-QP2010 or Shimadzu GC-2010 Plus and Shimadzu GCMS-TQ8030 using electron ionisation (EI) (70 keV). High resolution mass spectra were recorded using ESI (electrospray ionisation) by the Australian Proteome Analysis Facility. Infrared spectra were recorded using a Thermo Scientific Nicolet iS5 FTIR spectrometer equipped with an attenuated total reflectance (ATR) accessory. Peak positions from ATR-IR spectra are given in wavenumbers, $\tilde{\nu}$ (cm^{-1}). Melting points were measured with a Stuart SMP10 apparatus (Cole-Parmer, UK). Elemental analysis was performed by the Chemical Analysis Facility, Macquarie University using a Vario MICRO cube elemental analyser (Elementar Analysensysteme GmbH, Germany) with the reported values being an average of two duplicate runs. Flash column chromatography was performed using a Biotage Isolera Four over normal phase Merck 60 silica gel (40-60 μm) packed in a Biotage cartridge. The progress of all reactions was monitored with thin layer chromatography (TLC) and was performed using Merck TLC silica gel 60 F_{254} on aluminium sheets (0.2 mm) and visualised with ultraviolet light at 254 nm. Solvents were removed under reduced pressure using a Büchi Rotavapor R-200, Büchi V-500 vacuum pump, and Büchi B-490 heating bath set to a temperature of 40 °C, or a Büchi Rotavapor R-100, Vacuubrand PC 510 NT vacuum pump, Julabo F250 recirculating cooler, and Büchi B-100 heating bath set to a temperature of 40 °C. Drying following solvent removal was performed with an Alcatel Pascal 2005 SD high vacuum pump. Dry solvents were prepared by drying the solvent over 3 Å molecular sieves under an argon atmosphere. All reagents were purchased from Sigma-Aldrich, Merck, Alfa-Aesar, or Combi-Blocks and used without further purification.

General Synthesis Methods

General Method A – Steglich Esterification
To a solution of carboxylic acid (1.0 eq) in DCM (20.0 mL), was added DMAP (0.1 eq) then zingerone (1.0 eq). The colourless solution was cooled to 0 °C and DCC (1.1 eq) was slowly added. The white suspension was stirred at 0 °C for 5 minutes and then at room temperature for 1 hour. The white precipitate was removed by filtration and hydrochloric acid (80 mL, 1.0 mol L^{-1}) was added to the filtrate. The crude product was extracted with DCM (3 × 80 mL). The combined organic layers were dried with sodium sulfate and the solvent removed under reduced pressure to yield the crude product, which was purified by flash column chromatography (eluted with 0-60% DCM in hexane).

General Method B – Acid Anhydride Esterification
Acid anhydride (8.5 mmol, 1.5 eq) was added to zingerone (1.01 g, 5.20 mmol, 1.0 eq) and cooled to 0 °C. Pyridine (0.60 mL, 7.4 mmol, 1.5 eq) was added to the colourless solution, which was then heated to 80 °C and refluxed for 2 hours. The colourless solution was again cooled to 0 °C and hydrochloric acid (20.0 mL, 0.5 mol L^{-1}) was added. The colourless aqueous solution was extracted with DCM (2 × 20.0 mL). The organic layers were combined, washed with $NaHCO_3$ solution (2 × 20.0 mL, 10% (w/v)), and dried with sodium sulfate. The solvent was removed under reduced pressure to give the crude product, which was purified by flash column chromatography (eluted with 10-40% ethyl acetate in hexane).

General Method C – Aldol Reaction and Catalytic Hydrogenation for 2017 Field Trial
To a solution of substituted benzaldehyde (1.00 g) in acetone (20.0 mL) was slowly added sodium hydroxide solution (5.00 mL, 10% (w/v)). The mixture was then stirred at 50 °C for 60 minutes. After removing the reaction mixture from heat, hydrochloric acid (1.0 mol L^{-1}) was added to achieve a pH of 1-2. The crude product was then extracted with ethyl acetate (3 × 30.0 mL). The combined organic layers were dried with $MgSO_4$ and the solvent removed under reduced pressure to give the crude enone product. To a solution of crude enone (1.0 eq) in methanol (30.0 mL) was added powdered Rh/Al_2O_3 (0.5 wt%) (0.20 mol%). The reaction vessel was evacuated and filled with hydrogen gas by a balloon. The mixture was stirred at room temperature for 60 minutes. The mixture was then filtered and the solvent removed under reduced pressure to yield the crude product, which was purified by flash column chromatography (eluted with 0-40% ethyl acetate in hexane).

General Method D – Aldol Reaction for 2018 and 2019 Field Trials
To a solution of substituted benzaldehyde (20.0 mmol, 1.0 eq) in acetone (88 mL, 1.2 mol, 60 eq), was added water (88 mL). The suspension was heated to 40 °C and sodium hydroxide solution (5% (w/v)) (1.60 mL, 2.00 mmol, 0.1 eq) was added. The solution was stirred at 40 °C for 4 hours. The heat was removed and the suspension was acidified with hydrochloric acid (1.0 mol L^{-1}). The volume of the mixture was reduced *in vacuo*. The crude product was then extracted with ethyl acetate (3 × 80 mL) and the combined organic layers were dried with sodium sulfate. The solvent was removed under reduced pressure to yield the crude product, which was purified by flash column chromatography (eluted with 0-30% ethyl acetate in hexane).

General Method E – Catalytic Hydrogenation
To a solution of substituted alkene (35.0 mmol, 1.0 eq) in ethanol (100 mL) was added powdered Rh/C (5 wt%, wet support) (3.5 mol%). The reaction vessel was evacuated and filled with hydrogen gas by a balloon. The mixture was stirred at room temperature for 20 hours. The Rh/C was removed by filtration through celite and the solvent removed under

reduced pressure to yield the crude product, which was purified by flash column chromatography (eluted with 0-40% ethyl acetate in hexane).

General Method F – Benzyl Protection
Acetone (40.0 mL) was added to a mixture of substituted phenol (30.0 mmol, 1.0 eq), potassium carbonate (6.22 g, 45.0 mmol, 1.5 eq), and potassium iodide (0.996 g, 6.00 mmol, 0.2 eq). Benzyl bromide (5.64 g, 33.0 mmol, 1.1 eq) was slowly added to the white suspension, which was then stirred at room temperature for 18 hours. The white suspension was diluted with water (80. mL) and the crude product was extracted with ethyl acetate (3 × 80. mL). The combined organic layers were washed with sodium hydroxide solution (1.0 mol L^{-1}) and dried with sodium sulfate. The solvent was removed under reduced pressure to yield the crude product, which was washed with hexane to give the pure product.

General Method G – Catalytic Hydrogenation and Deprotection
To a solution of benzyl-protected enone (15.0 mmol, 1.0 eq) in ethyl acetate (75 mL), was added Rh/Al_2O_3 (0.5 wt%) (1.54 g, 0.500 mol%). The grey suspension was evacuated and filled with hydrogen gas by balloon. The mixture was stirred at room temperature for 2 hours. The grey suspension was filtered through celite and the volume adjusted to 75 mL. Pd/C (10 wt%) (0.160 g, 1.00 mol%) was added and the flask was evacuated and filled with hydrogen gas by balloon. The black suspension was stirred at room temperature for 2 hours. The black suspension was filtered through celite and the solvent removed under reduced pressure to yield the crude product, which was purified by flash column chromatography (eluted with 0-30% ethyl acetate in hexane).

General Method H – Aldol Reaction with 3-Methyl-2-butanone and 3,3-Dimethyl-2-butanone
To a colourless solution of 4-benzyloxy-3-methoxybenzaldehyde (4.85 g, 20.0 mmol, 1.0 eq) in THF/ethanol (250 mL, 1:1 (v/v)), was added 3-methyl-2-butanone or 3,3-dimethyl-2-butanone (60. mmol, 3.0 eq). The colourless solution was heated to reflux and sodium hydroxide (0.400 g, 10.0 mmol, 0.5 eq) was added. The colourless solution was stirred at reflux for 4 hours. The solution was allowed to return to room temperature before hydrochloric acid (80 mL, 1.0 mol L^{-1}) was added, which caused the solution to become yellow. The volume of the yellow solution was reduced *in vacuo*. The crude product was then extracted with DCM (3 × 80 mL) and the combined organic layers were dried with sodium sulfate. The solvent was removed under reduced pressure to yield the crude product, which was purified by flash column chromatography (eluted with 0-30% ethyl acetate in hexane).

General Method I – Formylation with Formic Acetic Mixed Anhydride
A colourless solution of formic acid (3.8 mL, 0.10 mol, 5.0 eq) and acetic anhydride (7.6 mL, 80 mmol, 4.0 eq) was stirred at 60 °C for 1 hour. This colourless solution was then allowed to cool to room temperature and was added to a mixture of substituted phenol or aniline (20.0 mmol, 1.0 eq) and sodium acetate (0.820 g, 10.0 mmol, 0.5 eq). The solution was stirred at room temperature for 1 hour before being diluted with ethyl acetate (80 mL) and then washed with water (3 × 80 mL). The organic layer was dried with sodium sulfate and the solvent removed under reduced pressure to yield the crude product, which was purified by flash column chromatography (eluted with 0-60% ethyl acetate in hexane).

General Method J – *N*-Methylation of Amides
To a grey suspension of sodium hydride (60% dispersion in mineral oil) (0.400 g, 10.0 mmol, 1.0 eq) in dry THF (10 mL) at 0 °C, a solution of amide (10.0 mmol, 1.0 eq) in dry THF (10 mL) was added dropwise. The yellow suspension was stirred at 0 °C for 1 hour. A solution of methyl iodide (0.81 mL, 13 mmol, 1.3 eq) in dry THF (10 mL) was added

dropwise over 5 minutes at 0 °C. The yellow suspension was heated to 45 °C and stirred for 3 hours. The reaction was quenched with the addition of water (50 mL). The crude product was extracted with ethyl acetate (3 × 50 mL) and the combined organic layers were dried with sodium sulfate. The solvent was removed under reduced pressure to yield the crude product, which was purified by flash column chromatography (eluted with 0-40% ethyl acetate in hexane).

General Method K – Acid Anhydride Amidation

To a yellow solution of 4-(4-amino-3-methoxyphenyl)-2-butanone (3.87 g, 20.0 mmol, 1.0 eq) in DCM (50 mL), was added an acid anhydride (30.0 mmol, 1.5 eq). The solution was stirred at room temperature for 1 hour before being washed with sodium hydrogen carbonate solution (10% (w/v)). The organic layer was dried with sodium sulfate and the solvent removed under reduced pressure to yield the crude product, which was purified by flash column chromatography (eluted with 0-60% ethyl acetate in hexane).

General Method L – Benzyl Ether Deprotection

To a solution of benzyl-protected phenol (10.0 mmol, 1.0 eq) in ethyl acetate (50 mL), was added Pd/C (10 wt%) (0.106 g, 1.00 mol%). The flask was evacuated and filled with hydrogen gas by balloon. The black suspension was stirred at room temperature for 1 hour. The Pd/C was removed by vacuum filtration through celite and the solvent was removed under reduced pressure to yield the crude product, which was purified by flash column chromatography (eluted with 0-30% ethyl acetate in hexane).

General Method M – Alkylation of Phenol

To a pale brown suspension of 4-(4-benzyloxy-3-hydroxyphenyl)-2-butanone (2.70 g, 10.0 mmol, 1.0 eq) and potassium carbonate (2.76 g, 20.0 mmol, 2.0 eq) in acetone (40 mL), was added an alkyl halide (20.0 mmol, 2.0 eq). The pale brown suspension was then heated at reflux for 40 hours. The pale brown suspension was allowed to cool to room temperature and water (40 mL) was added before the crude product was extracted with ethyl acetate (3 × 40 mL) and the combined organic layers were dried with sodium sulfate. The solvent was removed under reduced pressure to yield the crude product.

2017 Field Trial Compounds

4-(4-Formoxy-3-methoxyphenyl)-2-butanone (2)

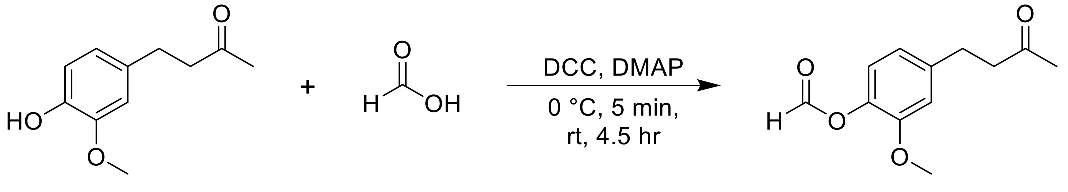

Synthesised using General Method A with formic acid (0.29 mL, 7.69 mmol, 1.5 eq), DMAP (62.9 mg, 0.515 mmol, 0.1 eq), zingerone (1.00 g, 5.15 mmol, 1.0 eq), and DCC (1.59 g, 7.71 mmol, 1.5 eq). The reaction was stirred at room temperature for 4.5 hours and the crude product was obtained after filtration and removal of the solvent. Flash column chromatography was performed with 0-25% ethyl acetate in hexane. Colourless oil (*impure*, 1.12 g, 98.0% yield, containing approximately 5% zingerone by ^{1}H NMR and GC-FID, R_f: 0.21 (3:1 (v/v) hexane:ethyl acetate)). ^{1}H NMR (400 MHz, $CDCl_3$): δ 2.15 (3H, s), 2.76 (2H, t, J = 7.5 Hz), 2.88 (2H, t, J = 7.4 Hz), 3.82 (3H, s), 6.76 (1H, dd, J = 1.5, 8.1 Hz), 6.82 (1H, s), 6.99 (1H, d, J = 8.1 Hz), 8.24 (1H, s) ppm. ^{13}C NMR (101 MHz, $CDCl_3$): δ 29.6, 30.2, 45.2, 56.0, 113.0, 120.6, 122.5, 137.2, 140.8, 150.7, 159.4, 207.7 ppm. HRMS (m/z): $[M+H]^+$

calc. for $C_{12}H_{15}O_4$, 223.09649; found, 223.09642. GC-MS (EI) *m/z* (% of base peak): 222 ($M^{\bullet+}$, 6.8), 194 ($M^{\bullet+}$-CO, 37.6), 151 ($M^{\bullet+}$-CO-$COCH_3$, 16.2), 137 ($M^{\bullet+}$-CO-CH_2COCH_3, 100.0), 124 (9.7), 122 (9.1), 119 (22.3), 91 (25.2), 79 (8.5), 77 (15.5), 65 (10.8), 51 (13.9). IR-ATR $\tilde{\nu}_{max}$: 2940 (C-H), 1738 (C=O, aldehyde), 1711 (C=O, ketone), 1604 (Ar C-C), 1508 (Ar C-C), 1311, 1151, 1125, 1095, 1030 cm^{-1}. Although no literature spectral data were available, experimental spectral data were consistent with that expected for the title compound.

4-(4-Acetoxy-3-methoxyphenyl)-2-butanone (3)

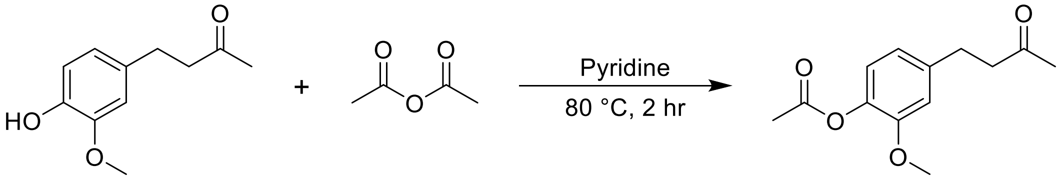

Synthesised using General Method B with acetic anhydride (0.80 mL, 8.5 mmol, 1.5 eq). White solid (1.01 g, 4.27 mmol, 82.2% yield, mp 43-44 °C (lit. mp 40-42 °C[1]), R_f: 0.16 (4:1 (v/v) hexane:ethyl acetate), elemental analysis C: 66.37% H: 6.97% (calc. C: 66.09% H: 6.83%)). ^{1}H NMR (400 MHz, $CDCl_3$): δ 2.15 (3H, s), 2.30 (3H, s), 2.76 (2H, t, *J* = 7.3 Hz), 2.88 (2H, t, *J* = 7.4 Hz), 3.81 (3H, s), 6.74 (1H, dd, *J* = 1.8, 8.0 Hz), 6.79 (1H, d, *J* = 1.7 Hz), 6.93 (1H, d, *J* = 8.0 Hz) ppm. ^{13}C NMR (101 MHz, $CDCl_3$): δ 20.8, 29.7, 30.3, 45.3, 56.0, 112.8, 120.5, 122.8, 138.1, 140.2, 151.0, 169.4, 207.9 ppm. GC-MS (EI) *m/z* (% of base peak): 236 ($M^{\bullet+}$, 5.8), 194 (72.6), 151 (18.3), 137 (100.0), 124 (12.2), 119 (17.4), 91 (10.5), 77 (4.9). Although no literature spectral data were available, experimental spectral data were consistent with that expected for the title compound.

4-(4-Propioxy-3-methoxyphenyl)-2-butanone (4)

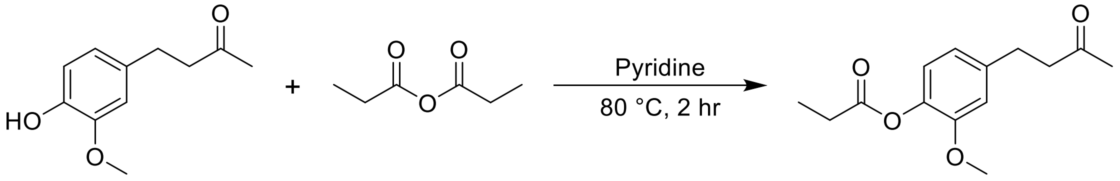

Synthesised using General Method B on a 25.9 mmol scale with propionic anhydride (5.01 g, 38.5 mmol, 1.5 eq). The reaction was neutralised with hydrochloric acid (1.0 mol L^{-1}) and the crude product extracted with DCM (3 × 30 mL). The organic layers were washed with $NaHCO_3$ solution (90 mL, 5% (w/v)). Flash column chromatography was performed with 0-20% ethyl acetate in hexane. Colourless liquid (6.17 g, 24.7 mmol, 95.2% yield, R_f: 0.14 (4:1 (v/v) hexane:ethyl acetate)). ^{1}H NMR (400 MHz, $CDCl_3$): δ 1.26 (3H, t, *J* = 7.6 Hz), 2.15 (3H, s), 2.67 (2H, t, *J* = 7.4 Hz), 2.76 (2H, t, *J* = 7.4 Hz), 2.87 (2H, t, *J* = 7.5 Hz), 3.80 (3H, s), 6.74 (1H, dd, *J* = 1.9, 8.0 Hz), 6.79 (1H, d, *J* = 1.8 Hz), 6.92 (1H, d, *J* = 8.0 Hz) ppm. ^{13}C NMR (101 MHz, $CDCl_3$): δ 9.2, 27.4, 29.6, 30.1, 45.2, 55.9, 112.7, 120.3, 122.6, 138.1, 139.9, 150.9, 172.7, 207.8 ppm. HRMS (*m/z*): $[M+H]^+$ calc. for $C_{14}H_{19}O_4$, 251.12779; found, 251.12767. GC-MS (EI) *m/z* (% of base peak): 250 ($M^{\bullet+}$, 4.5), 194 (83.1), 151 (21.0), 137 (100.0), 124 (14.0), 122 (3.2), 119 (13.8), 91 (7.2), 57 (7.0). Although no literature spectral data were available, experimental spectral data were consistent with that expected for the title compound.

4-(4-(3,3,3-Trifluoropropioxy)-3-methoxyphenyl)-2-butanone (5)

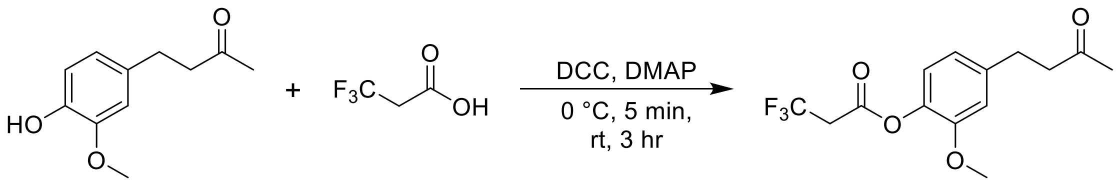

Synthesised using General Method A with 3,3,3-trifluoropropionic acid (3.51 g, 27.4 mmol, 1.0 eq) in DCM (100 mL), DMAP (335 mg, 2.74 mmol, 0.1 eq), zingerone (5.32 g, 27.4 mmol, 1.0 eq), and DCC (6.22 g, 30.1 mmol, 1.1 eq). The reaction was stirred at room temperature for 3 hours. The filtrate was washed with hydrochloric acid (100 mL, 0.1 mol L^{-1}), then $NaHCO_3$ solution (100 mL, 5% (w/v)). Flash column chromatography was performed with 0-20% ethyl acetate in hexane. White solid (7.39 g, 24.3 mmol, 88.7% yield, mp 62-63 °C, R_f: 0.13 (4:1 (v/v) hexane:ethyl acetate), elemental analysis C: 55.54% H: 5.10% (calc. C: 55.27% H: 4.97%)). ^{1}H NMR (400 MHz, $CDCl_3$): δ 2.15 (3H, s), 2.76 (2H, t, *J* = 7.5 Hz), 2.88 (2H, t, *J* = 7.4 Hz), 3.43 (2H, q, *J* = 9.9 Hz), 3.81 (3H, s), 6.76 (1H, dd, *J* = 1.9, 8.1 Hz), 6.81 (1H, d, *J* = 1.9 Hz), 6.95 (1H, d, *J* = 8.1 Hz) ppm. ^{13}C NMR (101 MHz, $CDCl_3$): δ 29.5, 30.1, 39.4 (q, *J* = 31.6 Hz), 45.1, 55.9, 112.8, 120.4, 122.2, 123.3 (q, *J* = 288 Hz), 137.3, 140.8, 150.6, 162.2 (q, *J* = 4.2 Hz), 207.6 ppm. ^{19}F NMR (376 MHz, $CDCl_3$): δ -64.37 (t, *J* = 9.6 Hz) ppm. GC-MS (EI) *m/z* (% of base peak): 304 ($M^{\bullet +}$, 37.1), 194 (68.2), 151 (22.5), 137 (100.0), 124 (13.4), 122 (4.5), 119 (17.1), 111 (4.4), 91 (11.1), 77 (4.4). Although no literature spectral data were available, experimental spectral data were consistent with that expected for the title compound.

Zingerol (4-(4-Hydroxy-3-methoxyphenyl)-2-butanol) (30)

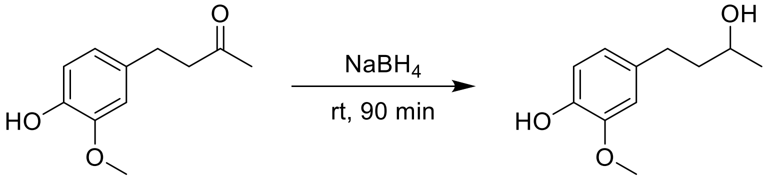

To a solution of zingerone (1.00 g, 5.15 mmol, 1.0 eq) in methanol (2.00 mL) was added a suspension of sodium borohydride (0.150 g, 3.97 mmol, 3.1 eq) in methanol (1.00 mL). The yellow suspension was stirred at room temperature for 90 minutes. Water (10.0 mL) was then added to the stirring yellow suspension. The product was extracted with ethyl acetate (3 × 10.0 mL) and the combined organic layers were washed with saturated brine solution (3 × 30.0 mL) and dried with $MgSO_4$. The solvent was removed under reduced pressure to yield the pure product as a very pale yellow oil (0.976 g, 4.97 mmol, 96.6% yield, R_f: 0.09 (3:1 (v/v) hexane:ethyl acetate)). ^{1}H NMR (400 MHz, $CDCl_3$): δ 1.23 (3H, d, *J* = 6.2 Hz), 1.75 (2H, m), 2.65 (2H, m), 3.83 (1H, m), 3.88 (3H, s), 5.46 (1H, br s), 6.69 (2H, m), 6.83 (1H, d, *J* = 7.7 Hz) ppm. ^{13}C NMR (101 MHz, $CDCl_3$): δ 23.8, 32.0, 41.3, 56.0, 67.7, 111.1, 114.4, 121.0, 134.1, 143.8, 146.6 ppm. HRMS (*m/z*): $[M]^+$ calc. for $C_{11}H_{16}O_3$, 196.10940; found, 196.10930. GC-MS (EI) *m/z* (% of base peak): 196 ($M^{\bullet +}$, 67.4), 163 (24.5), 147 (10.8), 138 (85.6), 137 ($M^{\bullet +}$-$CH_2CH(OH)CH_3$, 100.0), 131 (47.9), 123 (26.4), 122 (19.2), 107 (14.0), 106 (21.1), 91 (19.8), 77 (11.1). Experimental spectral data were consistent with literature data[2].

4-Hydroxy-3-methoxybenzyl acetate (31)

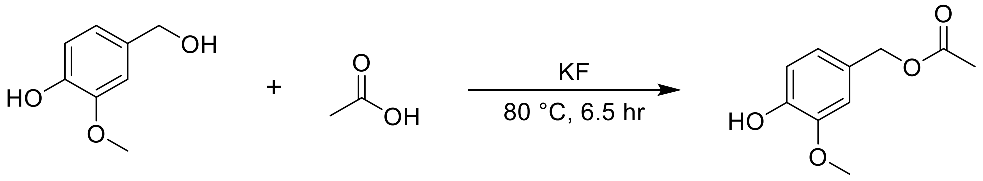

To vanillyl alcohol (5.00 g, 32.4 mmol, 1.0 eq) was added potassium fluoride (2.45 g, 42.2 mmol, 1.3 eq), then glacial acetic acid (75.0 mL, 1.31 mol, 40.4 eq). The colourless solution was stirred at 80 °C for 6.5 hours. Water (200 mL) was then added and the crude product was extracted with ethyl acetate (3 × 100 mL). The combined organic layers were washed with $NaHCO_3$ solution (300 mL, 5% (w/v)), then brine (300 mL) and dried with sodium sulfate. The solvent was then removed under reduced pressure to yield the crude product as a brown oil. The crude product was purified by flash column chromatography (eluted with 0-40% ethyl acetate in hexane) to yield the product as a white solid (5.09 g, 26.0 mmol, 80.0% yield, mp 47-48 °C (lit. mp 29-32 °C[3]), R_f: 0.30 (3:1 (v/v) hexane:ethyl acetate), elemental analysis C: 61.30% H: 6.33% (calc. C: 61.22% H: 6.17%)). ^{1}H NMR (400 MHz, $CDCl_3$): δ 2.09 (3H, s), 3.91 (3H, s), 5.02 (2H, s), 5.66 (1H, br s), 6.89 (3H, m) ppm. ^{13}C NMR (101 MHz, $CDCl_3$): δ 21.2, 56.1, 66.7, 111.5, 114.5, 122.3, 127.9, 146.0, 146.6, 171.1 ppm. GC-MS (EI) *m/z* (% of base peak): 196 ($M^{\bullet+}$, 66.0), 154 (60.7), 139 (11.8), 137 ($M^{\bullet+}$-$OCOCH_3$, 100.0), 136 (21.9), 123 (10.2), 122 (34.8), 107 (12.7), 94 (13.6), 93 (17.8), 79 (9.9), 65 (16.0). Experimental spectral data were consistent with literature data[4,5].

Piperonal (3,4-Methylenedioxybenzaldehyde)

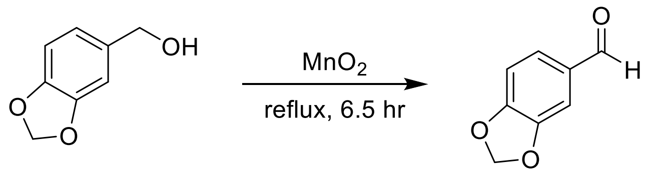

To a suspension of manganese dioxide (28.57 g, 0.329 mol, 10. eq) in DCM (250 mL) was added piperonyl alcohol (5.00 g, 32.9 mmol, 1.0 eq). The black suspension was heated to reflux for 6.5 hours. It was then filtered and the solvent removed under reduced pressure to yield a clear viscous oil. Further drying under high vacuum yielded the product as a white solid (4.48 g, 29.9 mmol, 90.8% yield, mp 33-34 °C (lit. mp 35-37 °C[6]), R_f: 0.42 (5:1 (v/v) hexane:ethyl acetate)). ^{1}H NMR (400 MHz, $CDCl_3$): δ 6.08 (2H, s), 6.93 (1H, d, J = 7.9 Hz), 7.34 (1H, d, J = 0.9 Hz), 7.41 (1H, dd, J = 1.0, 8.0 Hz), 9.81 (1H, s) ppm. ^{13}C NMR (101 MHz, $CDCl_3$): δ 102.2, 107.1, 108.5, 128.8, 132.0, 148.9, 153.2, 190.4 ppm. Experimental spectral data were consistent with literature data[6,7].

Methylzingerone (4-(3,4-Dimethoxyphenyl)-2-butanone) (8)

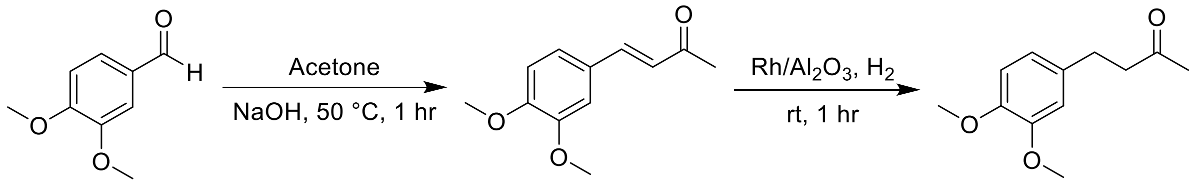

Synthesised using General Method C. White solid (1.02 g, 5.29 mmol, 81.4% yield, mp 55-56 °C (lit. mp 55-56 °C[1,8]), R_f: 0.19 (4:1 (v/v) hexane:ethyl acetate), elemental analysis

C: 69.11% H: 7.71% (calc. C: 69.21% H: 7.74%)). ^{1}H NMR (400 MHz, $CDCl_3$): δ 2.14 (3H, s), 2.74 (2H, t, *J* = 7.1 Hz), 2.85 (2H, t, *J* = 7.1 Hz), 3.85 (3H, s), 3.87 (3H, s) 6.72 (2H, m), 6.79 (1H, d, *J* = 4.3 Hz) ppm. ^{13}C NMR (101 MHz, $CDCl_3$): δ 29.5, 30.3, 45.6, 56.0, 56.1, 111.5, 111.9, 120.2, 133.8, 147.5, 149.0, 208.2 ppm. GC-MS (EI) *m/z* (% of base peak): 208 ($M^{•+}$, 54.2), 165 ($M^{•+}$-$COCH_3$, 28.6), 151 ($M^{•+}$-CH_2COCH_3, 100.0), 150 (6.8), 138 (5.5), 135 (5.4), 119 (5.0), 107 (8.5), 105 (5.8), 91 (9.6), 77 (6.5). Experimental spectral data were consistent with literature data[9,10].

Isozingerone (4-(3-Hydroxy-4-methoxyphenyl)-2-butanone) (9)

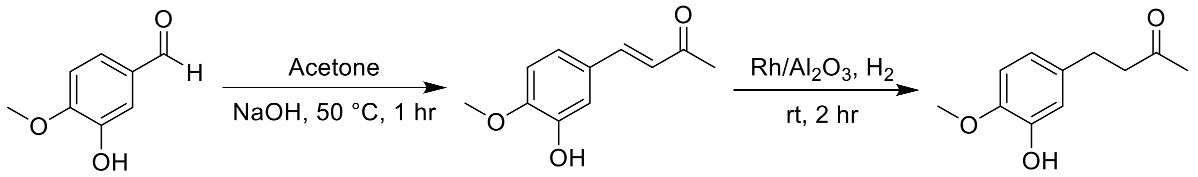

Synthesised using General Method C except the reaction mixture was stirred under hydrogen gas for 120 minutes instead of 60 minutes. Flash column chromatography was performed with 0-50% ethyl acetate in hexane. White solid (1.06 g, 5.44 mmol, 82.8% yield, mp 36-37 °C (lit. mp 28-29 °C[11] and 41-42 °C[12]), R_f: 0.21 (3:1 (v/v) hexane:ethyl acetate), elemental analysis C: 68.00% H: 7.44% (calc. C: 68.02% H: 7.27%)). ^{1}H NMR (400 MHz, $CDCl_3$): δ 2.13 (3H, s), 2.71 (2H, t, *J* = 7.3 Hz), 2.80 (2H, t, *J* = 7.3 Hz), 3.86 (3H, s), 5.59 (1H, br s), 6.65 (1H, dd, *J* = 2.0, 8.2 Hz), 6.76 (2H, m) ppm. ^{13}C NMR (101 MHz, $CDCl_3$): δ 29.3, 30.2, 45.4, 56.1, 110.8, 114.6, 119.8, 134.4, 145.1, 145.7, 208.3 ppm. GC-MS (EI) *m/z* (% of base peak): 194 ($M^{•+}$, 62.7), 151 ($M^{•+}$-$COCH_3$, 32.7), 137 ($M^{•+}$-CH_2COCH_3, 100.0), 124 (8.1), 122 (14.0), 119 (33.9), 107 (6.4), 94 (5.8), 91 (23.1), 79 (5.9), 77 (7.4), 65 (4.9). Although no literature spectral data were available, experimental spectral data were consistent with that expected for the title compound.

4-(3,4-Methylenedioxyphenyl)-2-butanone (10)

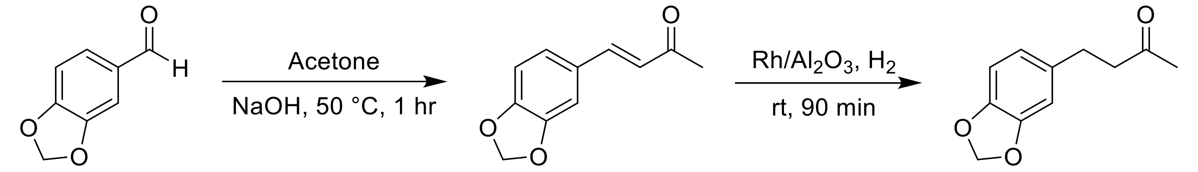

Synthesised using General Method C except water (20.0 mL) was added instead of hydrochloric acid and the reaction mixture was stirred under hydrogen gas for 90 minutes instead of 60 minutes. White solid (1.10 g, 5.73 mmol, 86.1% yield, mp 44-46 °C (lit. mp 50 °C[13] and 55 °C[14]), R_f: 0.37 (5:1 (v/v) hexane:ethyl acetate), elemental analysis C: 68.66% H: 6.07% (calc. C: 68.74% H: 6.29%)). ^{1}H NMR (400 MHz, $CDCl_3$): δ 2.13 (3H, s), 2.71 (2H, t, *J* = 7.1 Hz), 2.81 (2H, t, *J* = 7.1 Hz), 5.91 (2H, s), 6.62 (1H, dd, *J* = 1.4, 7.9 Hz), 6.67 (1H, d, *J* = 1.2 Hz), 6.72 (1H, d, *J* = 7.9 Hz) ppm. ^{13}C NMR (101 MHz, $CDCl_3$): δ 29.6, 30.3, 45.6, 101.0, 108.4, 108.9, 121.2, 134.9, 146.0, 147.8, 208.1 ppm. GC-MS (EI) *m/z* (% of base peak): 192 ($M^{•+}$, 58.6), 149 ($M^{•+}$-$COCH_3$, 22.5), 147 (6.6), 135 ($M^{•+}$-CH_2COCH_3, 100.0), 122 (7.5), 119 (29.1), 105 (5.0), 91 (19.1), 79 (7.7), 77 (16.2), 65 (7.9), 51 (8.4). Experimental spectral data were consistent with literature data[9].

4-(3,4-(Difluoromethylenedioxy)phenyl)-2-butanone (11)

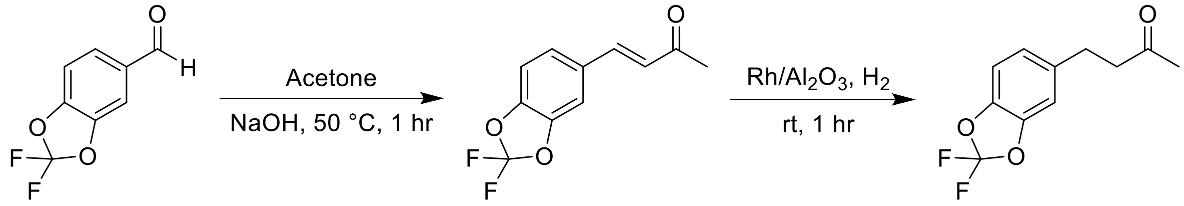

Synthesised using General Method C except water (20.0 mL) was added instead of hydrochloric acid and the crude product was purified by Kugelrohr distillation instead of flash column chromatography. Colourless liquid (0.836 g, 3.66 mmol, 68.1% yield, R_f: 0.36 (5:1 (v/v) hexane:ethyl acetate)). ^{1}H NMR (400 MHz, $CDCl_3$): δ 2.14 (3H, s), 2.74 (2H, t, *J* = 7.3 Hz), 2.88 (2H, t, *J* = 7.3 Hz), 6.87 (1H, dd, *J* = 1.5, 8.1 Hz), 6.91 (1H, d, *J* = 1.6 Hz), 6.94 (1H, d, *J* = 8.2 Hz) ppm. ^{13}C NMR (101 MHz, $CDCl_3$): δ 29.5, 30.2, 45.2, 109.4, 109.8, 123.4, 131.8 (t, *J* = 255.8 Hz), 137.3, 142.3, 144.0, 207.4 ppm. ^{19}F NMR (376 MHz, $CDCl_3$): δ -50.82 ppm. HRMS (*m*/*z*): $[M\text{-}H]^-$ calc. for $C_{11}H_9F_2O_3$, 227.05252; found, 227.05204. GC-MS (EI) *m/z* (% of base peak): 228 ($M^{\bullet+}$, 99.9), 185 ($M^{\bullet+}$-$COCH_3$, 59.7), 171 ($M^{\bullet+}$-CH_2COCH_3, 100.0), 119 (22.8), 105 (30.6), 91 (59.7), 89 (25.7), 77 (49.3), 65 (40.8), 63 (32.9), 51 (60.6), 50 (17.7). IR-ATR $\tilde{\nu}_{max}$: 2935 (C-H), 1715 (C=O, ketone), 1498 (Ar C-C), 1447, 1232, 1143, 1035, 805, 703 cm^{-1}. Although no literature spectral data were available, experimental spectral data were consistent with that expected for the title compound.

4-(4-Hydroxy-2-methoxyphenyl)-2-butanone (20)

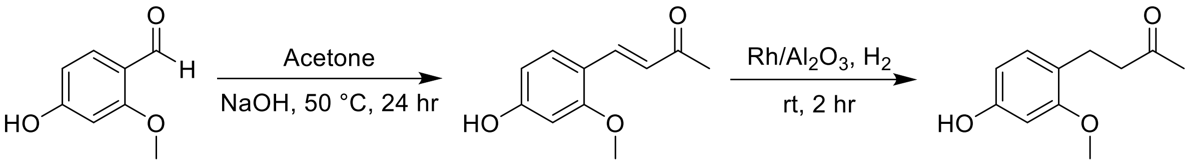

Synthesised using General Method C except the aldol reaction mixture was stirred at 50 °C for 24 hours and the hydrogenation reaction mixture was stirred under hydrogen gas for 120 minutes instead of 60 minutes. Flash column chromatography was performed with 0-50% ethyl acetate in hexane. White solid (1.06 g, 5.44 mmol, 82.7% yield, mp 113-115 °C (lit. mp 116-118 °C[15]), R_f: 0.19 (3:1 (v/v) hexane:ethyl acetate), elemental analysis C: 67.82% H: 7.34% (calc. C: 68.02% H: 7.27%)). ^{1}H NMR (400 MHz, $CDCl_3$): δ 2.13 (3H, s), 2.68 (2H, t, *J* = 7.4 Hz), 2.80 (2H, t, *J* = 7.3 Hz), 3.78 (3H, s), 5.01 (1H, br s), 6.31 (1H, dd, *J* = 2.4, 8.1 Hz), 6.39 (1H, d, *J* = 2.4 Hz), 6.95 (1H, d, *J* = 8.1 Hz) ppm. ^{13}C NMR (101 MHz, $CDCl_3$): δ 24.6, 30.1, 44.2, 55.3, 99.0, 106.7, 121.3, 130.5, 155.6, 158.5, 209.9 ppm. GC-MS (EI) *m/z* (% of base peak): 194 ($M^{\bullet+}$, 36.6), 151 ($M^{\bullet+}$-$COCH_3$, 9.0), 137 ($M^{\bullet+}$-CH_2COCH_3, 100.0), 124 (15.4), 107 (32.9), 91 (4.9), 79 (6.9), 77 (11.0), 65 (4.3). Although no literature spectral data were available, experimental spectral data were consistent with that expected for the title compound.

4-(4-Hydroxy-3-(trifluoromethoxy)phenyl)-2-butanone (24)

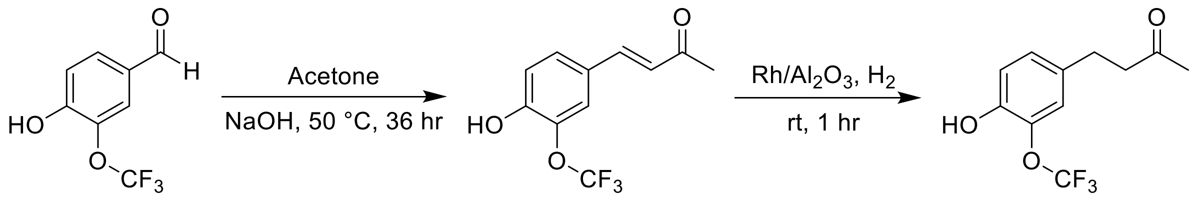

Synthesised using General Method C at half scale; 0.500 g of 4-hydroxy-3-(trifluoromethoxy)benzaldehyde instead of 1.00 g and 15.0 mL of methanol instead of 30.0 mL. Aldol reaction mixture was stirred at 50 °C for 36 hours. White solid (0.483 g, 1.95 mmol, 80.2% yield, mp 59-61 °C, R_f: 0.24 (4:1 (v/v) hexane:ethyl acetate), elemental analysis C: 53.31% H: 3.94% (calc. C: 53.23% H: 4.47%)). ^{1}H NMR (400 MHz, $CDCl_3$): δ 2.14 (3H, s), 2.73 (2H, t, *J* = 7.2 Hz), 2.83 (2H, t, *J* = 7.1 Hz), 5.67 (1H, br s), 6.93 (1H, d, *J* = 8.3 Hz), 7.00 (1H, dd, *J* = 1.7, 8.3 Hz), 7.03 (1H, br s) ppm. ^{13}C NMR (101 MHz, $CDCl_3$): δ 28.8, 30.2, 45.2, 117.5, 120.8 (q, *J* = 259.9 Hz), 121.4, 128.0, 133.9, 136.4 (*J* = 1.3 Hz), 146.3, 208.2 ppm. ^{19}F NMR (376 MHz, $CDCl_3$): δ -58.64 ppm. GC-MS (EI) *m/z* (% of base peak): 248 ($M^{\bullet+}$, 100.0), 233 ($M^{\bullet+}$-CH_3, 18.7), 191 ($M^{\bullet+}$-CH_2COCH_3, 85.8), 171 (78.9), 105 (53.9), 91 (63.8), 89 (24.5), 77 (67.8), 69 (18.3), 65 (40.0), 63 (19.8), 51 (53.1). IR-ATR $\tilde{\nu}_{max}$: 3304 (O-H), 2939 (C-H), 1693 (C=O, ketone), 1520 (Ar C-C), 1243, 1199, 1163, 1144, 1114, 819 cm^{-1}. Although no literature spectral data were available, experimental spectral data were consistent with that expected for the title compound.

2018 Field Trial Compounds

4-(4-Isobutyroxy-3-methoxyphenyl)-2-butanone (6)

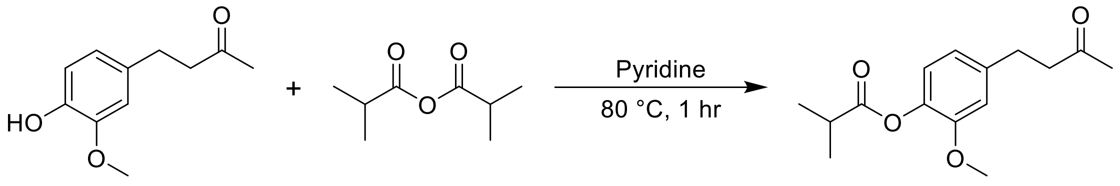

Synthesised using General Method B with isobutyric anhydride (3.32 mL, 3.17 g, 20.0 mmol, 1.0 eq), zingerone (3.88 g, 20.0 mmol, 1.0 eq), and pyridine (1.61 mL, 1.58 g, 20.0 mmol, 1.0 eq). The reaction time was 1 hour. The reaction was neutralised with hydrochloric acid (40.0 mL, 1.0 mol L^{-1}) and the crude product extracted with DCM (3 × 40.0 mL). The organic layers were washed with $NaHCO_3$ solution (40.0 mL, 10% (w/v)). Flash column chromatography was performed with 0-35% ethyl acetate in hexane. White solid (5.05 g, 19.1 mmol, 95.6% yield, mp 32-33 °C, R_f: 0.39 (3:1 (v/v) hexane:ethyl acetate), elemental analysis C: 68.61% H: 7.76% (calc. C: 68.16% H: 7.63%)). ^{1}H NMR (400 MHz, $CDCl_3$): δ 1.31 (6H, d, *J* = 7.0 Hz), 2.14 (3H, s), 2.75 (2H, t, *J* = 7.4 Hz), 2.82 (1H, m, *J* = 7.0 Hz), 2.87 (2H, t, *J* = 7.5 Hz), 3.79 (3H, s), 6.74 (1H, dd, *J* = 1.8, 8.0 Hz), 6.78 (1H, d, *J* = 1.8 Hz), 6.91 (1H, d, *J* = 8.0 Hz) ppm. ^{13}C NMR (101 MHz, $CDCl_3$): δ 19.1, 29.7, 30.2, 34.1, 45.3, 56.0, 112.8, 120.4, 122.7, 138.3, 139.9, 151.1, 175.5, 207.9 ppm. GC-MS (EI) *m/z* (% of base peak): 264 ($M^{\bullet+}$, 4.8), 194 (100.0), 151 (18.2), 137 (86.5), 124 (13.7), 119 (11.5), 91 (6.9), 71 (6.5). Although no literature spectral data were available, experimental spectral data were consistent with that expected for the title compound.

4-(4-(3,3,3-Trifluoro-2-(trifluoromethyl)propioxy)-3-methoxyphenyl)-2-butanone (7)

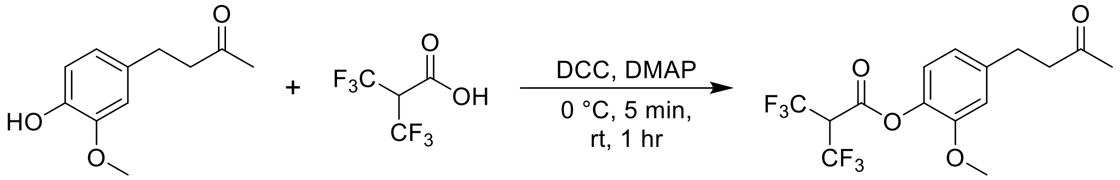

Synthesised using General Method A with 3,3,3-trifluoro-2-(trifluoromethyl)propionic acid (3.92 g, 20.0 mmol, 1.0 eq), DMAP (0.611 g, 5.00 mmol, 0.25 eq), zingerone (3.88 g, 20.0 mmol, 1.0 eq), and DCC (4.54 g, 22.0 mmol, 1.1 eq). White solid (2.08 g, 5.58 mmol, 28.0% yield, mp 93-96 °C, R_f: 0.36 (3:1 (v/v) hexane:ethyl acetate), elemental analysis C: 48.87% H: 4.03% (calc. C: 48.40% H: 3.79%)). ^{1}H NMR (400 MHz, $CDCl_3$): δ 2.15 (3H, s), 2.76 (2H, t, *J* = 7.3 Hz), 2.89 (2H, t, *J* = 7.4 Hz), 3.80 (3H, s), 4.21 (1H, sep, *J* = 7.3 Hz), 6.76 (1H, dd, *J* = 1.9, 8.1 Hz), 6.82 (1H, d, *J* = 1.8 Hz), 6.96 (1H, d, *J* = 8.1 Hz) ppm. ^{13}C NMR (101 MHz, $CDCl_3$): δ 29.7, 30.2, 45.1, 54.3 (sep, *J* = 122 Hz), 56.0, 113.1, 120.5, 120.9 (q, *J* = 284 Hz), 121.9, 137.2, 141.6, 150.5, 157.9 (m, *J* = 2.5 Hz), 207.6 ppm. ^{19}F NMR (376 MHz, $CDCl_3$): δ -65.33 (d, *J* = 7.9 Hz) ppm. GC-MS (EI) *m/z* (% of base peak): 372 ($M^{\bullet+}$, 71.2), 329 ($M^{\bullet+}$-$COCH_3$, 14.6), 295 (5.1), 194 (66.1), 179 (6.1), 151 (44.1), 150 (7.7), 137 (100.0), 136 (7.5), 124 (14.2), 119 (23.0), 91 (13.9). Although no literature spectral data were available, experimental spectral data were consistent with that expected for the title compound.

3-Methoxy-4-nitrobenzaldehyde

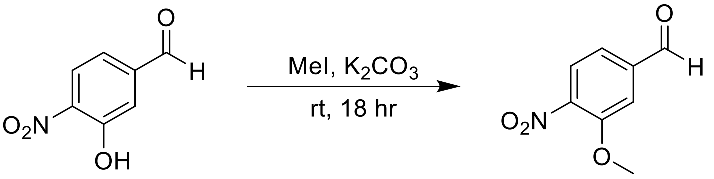

To a yellow suspension of 3-hydroxy-4-nitrobenzaldehyde (8.36 g, 50.0 mmol, 1.0 eq) and potassium carbonate (8.98 g, 65.0 mmol, 1.3 eq) in DMF (40 mL), was added methyl iodide (9.23 g, 65.0 mmol, 1.3 eq). The yellow/red suspension was stirred at room temperature for 18 hours. The red suspension was diluted with water (160 mL) and the precipitate was collected by vacuum filtration and washed with water and dried, yielding the product as an off-white solid (8.10 g, 44.7 mmol, 89.4% yield, mp 98-100 °C (lit. mp 97-98 °C[16] and 97-99 °C[17]), R_f: 0.29 (4:1 (v/v) hexane:ethyl acetate)). ^{1}H NMR (400 MHz, $CDCl_3$): δ 4.03 (3H, s), 7.54 (1H, dd, *J* = 1.5, 8.1 Hz), 7.60 (1H, d, *J* = 1.4 Hz), 7.92 (1H, d, *J* = 8.1 Hz), 10.05 (1H, s) ppm. ^{13}C NMR (101 MHz, $CDCl_3$): δ 56.9, 112.7, 122.7, 126.1, 139.8, 143.4, 153.1, 190.4 ppm. Experimental spectral data were consistent with literature data[18,19].

4-(3-Methoxy-4-nitrophenyl)but-3-en-2-one

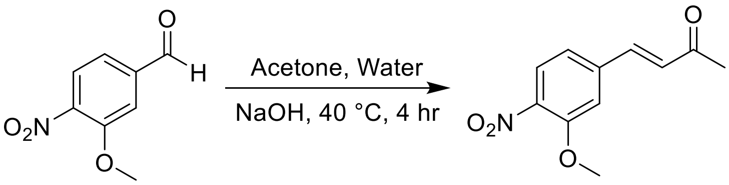

Synthesised using General Method D on a 40.0 mmol scale with 3-methoxy-4-nitrobenzaldehyde (7.25 g, 40.0 mmol, 1.0 eq) and water (440 mL). The crude product was

collected by vacuum filtration, washed with water and dried, yielding the pure product as a yellow solid (8.30 g, 37.5 mmol, 93.8% yield, mp 126-128 °C, R_f: 0.15 (3:1 (v/v) hexane:ethyl acetate), elemental analysis C: 59.97% H: 4.93% N: 5.99% (calc. C: 59.73% H: 5.01% N: 6.33%)). ^{1}H NMR (400 MHz, $CDCl_3$): δ 2.41 (3H, s), 3.99 (3H, s), 6.76 (1H, d, *J* = 16.2 Hz), 7.19 (2H, m), 7.46 (1H, d, *J* = 16.3 Hz), 7.86 (1H, d, *J* = 8.7 Hz) ppm. ^{13}C NMR (101 MHz, $CDCl_3$): δ 28.1, 56.7, 112.9, 119.9, 126.4, 130.2, 140.2, 140.5, 140.6, 153.4, 197.7 ppm. GC-MS (EI) *m/z* (% of base peak): 221 ($M^{\bullet+}$, 56.1), 206 ($M^{\bullet+}$-CH_3, 57.0), 204 (72.8), 190 (16.1), 173 (43.9), 160 (100.0), 145 (31.7), 132 (23.3), 131 (26.9), 130 (20.9), 102 (59.2), 89 (28.1). Although no literature spectral data were available, experimental spectral data were consistent with that expected for the title compound.

4-(4-Amino-3-methoxyphenyl)-2-butanone (12)

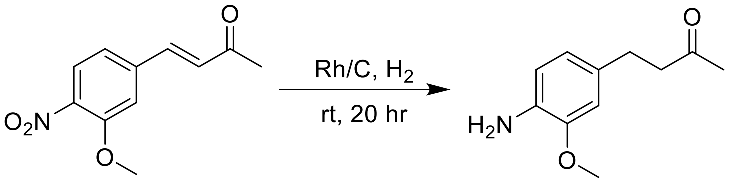

Synthesised using General Method E with 4-(3-methoxy-4-nitrophenyl)but-3-en-2-one (7.74 g, 35.0 mmol 1.0 eq). Yellow solid (4.25 g, 22.0 mmol, 62.8% yield, mp 45-47 °C (lit. mp 40 °C[20]), R_f: 0.24 (3:1 (v/v) hexane:ethyl acetate), elemental analysis C: 68.63% H: 7.86% N: 7.00% (calc. C: 68.37% H: 7.82% N: 7.25%)). ^{1}H NMR (400 MHz, $CDCl_3$): δ 2.13 (3H, s), 2.71 (2H, m), 2.80 (2H, m), 3.69 (2H, br s), 3.83 (3H, s), 6.61 (3H, m) ppm. ^{13}C NMR (101 MHz, $CDCl_3$): δ 29.6, 30.3, 45.8, 55.5, 110.9, 115.1, 120.5, 131.4, 134.3, 147.4, 208.6 ppm. GC-MS (EI) *m/z* (% of base peak): 193 ($M^{\bullet+}$, 32.4), 136 ($M^{\bullet+}$-CH_2COCH_3, 100.0), 121 (20.1), 119 (4.3), 106 (7.0), 104 (4.4), 93 (8.2), 91 (5.0), 77 (5.1), 66 (4.2). Although no literature spectral data were available, experimental spectral data were consistent with that expected for the title compound.

4-Benzyloxy-3-ethoxybenzaldehyde

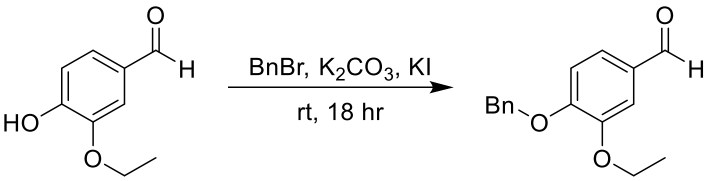

Synthesised using General Method F with ethylvanillin (4.99 g, 30.0 mmol, 1.0 eq). White solid (*impure*, 7.16 g, 27.9 mmol, 93.1% yield, containing approximately 7% (4-(4-benzyloxy-3-ethoxyphenyl)-4-hydroxy-2-butanone by ^{1}H-NMR, R_f: 0.39 (5:1 (v/v) hexane:ethyl acetate)). ^{1}H NMR (400 MHz, $CDCl_3$): δ 1.49 (3H, t, *J* = 7.0 Hz), 4.18 (2H, q, *J* = 7.0 Hz), 5.25 (2H, s), 6.99 (1H, d, *J* = 8.2 Hz), 7.37 (7H, m), 9.82 (1H, s) ppm. ^{13}C NMR (101 MHz, $CDCl_3$): δ 14.8, 64.7, 70.9, 111.0, 113.0, 126.5, 127.1, 128.2, 128.8, 130.5, 136.4, 149.6, 154.0, 191.1 ppm. GC-MS (EI) *m/z* (% of base peak): 256 ($M^{\bullet+}$, 12.0), 165 (1.3), 137 (1.0), 119 (1.7), 109 (1.0), 91 ($Bn^{\bullet+}$, 100.0), 89 (1.2), 81 (1.2), 65 (6.4). Although no literature spectral data were available, experimental spectral data were consistent with that expected for the title compound.

4-(4-Benzyloxy-3-ethoxyphenyl)but-3-en-2-one

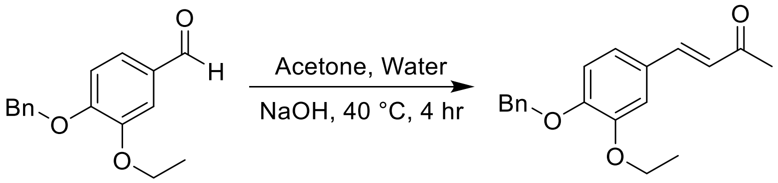

Synthesised using General Method D with 4-benzyloxy-3-ethoxybenzaldehyde (5.13 g, 20.0 mmol, 1.0 eq). Yellow solid (5.57 g, 18.8 mmol, 93.9% yield, mp 115-117 °C, R_f: 0.21 (5:1 (v/v) hexane:ethyl acetate), elemental analysis C: 76.68% H: 6.63% (calc. C: 77.00% H: 6.80%)). ^{1}H NMR (400 MHz, $CDCl_3$): δ 1.48 (3H, t, *J* = 7.0 Hz), 2.36 (3H, s), 4.14 (2H, q, *J* = 7.0 Hz), 5.19 (2H, s), 6.58 (1H, d, *J* = 16.2 Hz), 6.89 (1H, d, *J* = 8.3 Hz), 7.05 (1H, dd, *J* = 2.0, 8.3 Hz), 7.10 (1H, d, *J* = 2.0 Hz), 7.37 (6H, m) ppm. ^{13}C NMR (101 MHz, $CDCl_3$): δ 15.0, 27.5, 64.8, 71.0, 112.3, 114.3, 122.9, 125.5, 127.2, 127.9, 128.0, 128.7, 136.9, 143.7, 149.4, 151.0, 198.5 ppm. GC-MS (EI) *m/z* (% of base peak): 296 ($M^{\bullet+}$, 19.3), 254 (11.2), 252 (3.3), 205 (6.9), 177 (9.4), 149 (8.7), 121 (2.5), 91 ($Bn^{\bullet+}$, 100.0), 77 (2.5), 65 (5.0). Although no literature spectral data were available, experimental spectral data were consistent with that expected for the title compound.

4-(3-Ethoxy-4-hydroxyphenyl)-2-butanone (25)

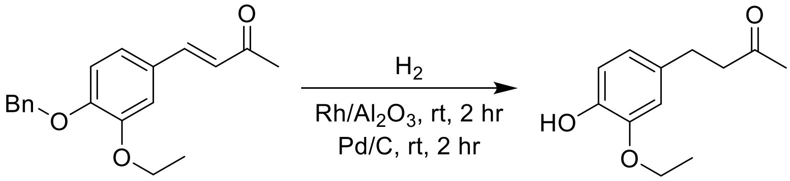

Synthesised using General Method G. White solid (2.81 g, 13.5 mmol, 90.0% yield, mp 42-44 °C (lit. mp 42-43 °C[21]), R_f: 0.24 (4:1 (v/v) hexane:ethyl acetate), elemental analysis C: 69.27% H: 7.79% (calc. C: 69.21% H: 7.74%)). ^{1}H NMR (400 MHz, $CDCl_3$): δ 1.43 (3H, t, *J* = 7.0 Hz), 2.13 (3H, s), 2.72 (2H, t, *J* = 7.6 Hz), 2.81 (2H, t, *J* = 7.6 Hz), 4.09 (2H, q, *J* = 7.0 Hz), 5.57 (1H, s), 6.65 (1H, dd, *J* = 1.9, 8.0 Hz), 6.68 (1H, d, *J* = 1.9 Hz), 6.82 (1H, d, *J* = 8.0 Hz) ppm. ^{13}C NMR (101 MHz, $CDCl_3$): δ 15.0, 29.6, 30.3, 45.7, 64.5, 112.1, 114.4, 120.8, 132.9, 144.2, 145.8, 208.3 ppm. GC-MS (EI) *m/z* (% of base peak): 208 ($M^{\bullet+}$, 94.9), 165 ($M^{\bullet+}$-$COCH_3$, 18.6), 151 ($M^{\bullet+}$-CH_2COCH_3, 81.2), 147 (6.5), 138 (11.7), 137 (45.1), 123 (100.0), 119 (37.0), 110 (11.5), 105 (5.2), 91 (21.0), 77 (9.4). Although no literature spectral data were available, experimental spectral data were consistent with that expected for the title compound.

4-Benzyloxy-3-methoxybenzaldehyde

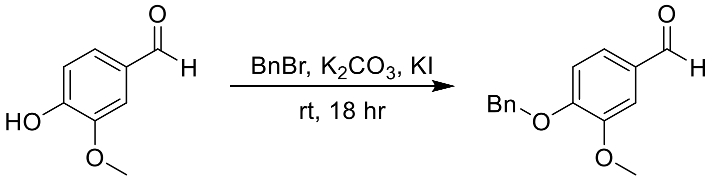

Synthesised using General Method F on a 200. mmol scale with vanillin (30.4 g, 200. mmol, 1.0 eq) and acetone (250 mL). At the end of the reaction, the white suspension was diluted with water (200 mL), the volume reduced *in vacuo*, and the crude product extracted with ethyl acetate (3 × 200 mL). Pale yellow solid (44.2 g, 182 mmol, 91.3% yield, mp 60-61 °C

(lit. mp 60-62 °C[22] and 61-62 °C[23]), R_f: 0.46 (3:1 (v/v) hexane:ethyl acetate)). ^{1}H NMR (400 MHz, $CDCl_3$): δ 3.95 (3H, s), 5.24 (2H, s), 6.99 (1H, d, *J* = 8.2 Hz), 7.38 (7H, m), 9.83 (1H, s) ppm. ^{13}C NMR (101 MHz, $CDCl_3$): δ 56.2, 71.0, 109.5, 112.5, 126.7, 127.3, 128.3, 128.8, 130.4, 136.1, 150.2, 153.7, 191.0 ppm. Experimental spectral data were consistent with literature data[24,25].

1-(4-Benzyloxy-3-methoxyphenyl)pent-1-en-3-one

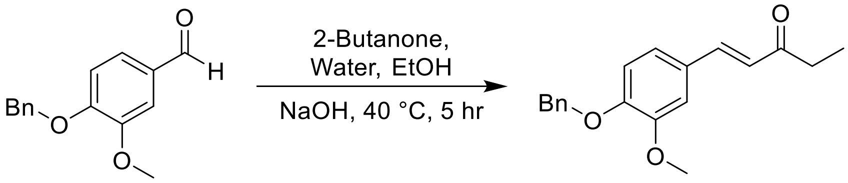

Synthesised using General Method D with 4-benzyloxy-3-methoxybenzaldehyde (4.85 g, 20.0 mmol, 1.0 eq), 2-butanone (108 mL, 1.21 mol, 60 eq) in place of acetone, and water (27 mL). Sufficient ethanol (24 mL) was added to give a homogenous solution. The reaction time was 5 hours. Water (80 mL) was added before extraction with ethyl acetate. The crude product was purified by recrystallisation from aqueous ethanol (approximately 70% (v/v)). Pale yellow needles (4.55 g, 15.4 mmol, 76.8% yield, mp 119-120 °C, R_f: 0.26 (4:1 (v/v) hexane:ethyl acetate), elemental analysis C: 77.18% H: 6.79% (calc. C: 77.00% H: 6.80%)). ^{1}H NMR (400 MHz, $CDCl_3$): δ 1.16 (3H, t, *J* = 7.3 Hz), 2.68 (2H, q, *J* = 7.3 Hz), 3.92 (3H, s), 5.19 (2H, s), 6.61 (1H, d, *J* = 16.1 Hz), 6.88 (1H, d, *J* = 8.3 Hz), 7.05 (1H, dd, *J* = 2.0, 8.3 Hz), 7.09 (1H, d, *J* = 2.0 Hz), 7.37 (5H, m), 7.49 (1H, d, *J* = 16.1 Hz) ppm. ^{13}C NMR (101 MHz, $CDCl_3$): δ 8.5, 33.9, 56.1, 71.0, 110.4, 113.6, 122.8, 124.4, 127.3, 128.0, 128.1, 128.8, 136.7, 142.3, 149.9, 150.5, 201.0 ppm. GC-MS (EI) *m/z* (% of base peak): 296 ($M^{\bullet+}$, 16.7), 267 ($M^{\bullet+}$-CH_2CH_3, 3.6), 240 (11.5), 205 (7.9), 177 (1.6), 117 (1.6), 107 (1.8), 91 ($Bn^{\bullet+}$, 100.0), 89 (1.8), 65 (4.5), 57 (1.5). Although no literature spectral data were available, experimental spectral data were consistent with that expected for the title compound.

1-(4-Hydroxy-3-methoxyphenyl)-3-pentanone (34)

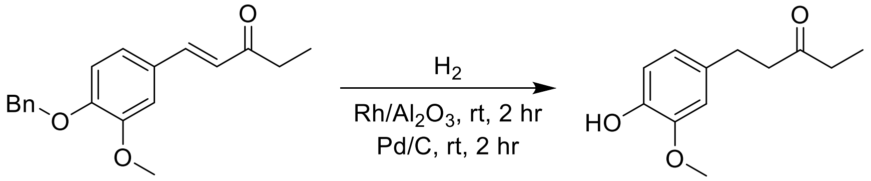

Synthesised using General Method G. White solid (2.90 g, 13.9 mmol, 92.9% yield, mp 37-39 °C (lit. mp 36-37 °C[26]), R_f: 0.22 (4:1 (v/v) hexane:ethyl acetate), elemental analysis C: 69.76% H: 7.96% (calc. C: 69.21% H: 7.74%)). ^{1}H NMR (400 MHz, $CDCl_3$): δ 1.04 (3H, t, *J* = 7.3 Hz), 2.40 (2H, q, *J* = 7.3 Hz), 2.70 (2H, t, *J* = 7.5 Hz), 2.83 (2H, t, *J* = 7.5 Hz), 3.86 (3H, s), 5.51 (1H, s), 6.66 (1H, dd, *J* = 1.8, 8.0 Hz), 6.69 (1H, d, *J* = 1.9 Hz), 6.82 (1H, d, *J* = 8.0 Hz) ppm. ^{13}C NMR (101 MHz, $CDCl_3$): δ 7.9, 29.7, 36.3, 44.4, 56.0, 111.2, 114.4, 120.9, 133.2, 144.0, 146.5, 211.0 ppm. GC-MS (EI) *m/z* (% of base peak): 208 ($M^{\bullet+}$, 47.0), 179 ($M^{\bullet+}$-CH_2CH_3, 15.5), 151 ($M^{\bullet+}$-$COCH_2CH_3$, 18.5), 147 (5.4), 137 ($M^{\bullet+}$-$CH_2COCH_2CH_3$, 100.0), 124 (7.5), 122 (8.4), 119 (21.4), 91 (13.0), 57 (7.8). Experimental spectral data were consistent with that expected for the title compound and with literature data[27].

1-(4-Benzyloxy-3-methoxyphenyl)-4-methylpent-1-en-3-one

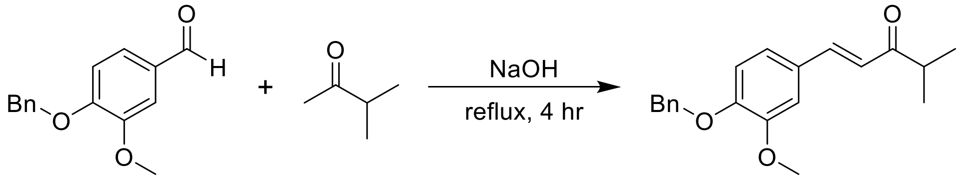

Synthesised using General Method H with 3-methyl-2-butanone (6.4 mL, 60. mmol, 3.0 eq). Pale yellow solid (4.01 g, 12.9 mmol, 64.6% yield, mp 65-69 °C, R_f: 0.38 (5:1 (v/v) hexane:ethyl acetate)). ^{1}H NMR (400 MHz, $CDCl_3$): δ 1.18 (6H, d, *J* = 6.9 Hz), 2.94 (1H, sep, *J* = 6.9 Hz), 3.93 (3H, s), 5.19 (2H, s), 6.67 (1H, d, *J* = 15.9 Hz), 6.88 (1H, d, *J* = 8.2 Hz), 7.07 (1H, dd, *J* = 2.0, 8.2 Hz), 7.10 (1H, d, *J* = 2.0 Hz), 7.36 (5H, m), 7.54 (1H, d, *J* = 16.0 Hz) ppm. ^{13}C NMR (101 MHz, $CDCl_3$): δ 18.7, 39.1, 56.1, 71.0, 110.6, 113.6, 122.8, 122.8, 127.3, 128.1, 128.1, 128.7, 136.7, 142.5, 149.9, 150.4, 203.9 ppm. GC-MS (EI) *m/z* (% of base peak): 310 ($M^{•+}$, 18.2), 267 ($M^{•+}$-$CH(CH_3)_2$, 12.6), 240 (10.9), 219 (6.9), 189 (1.5), 159 (4.0), 131 (3.3), 91 ($Bn^{•+}$, 100.0), 89 (1.9), 65 (4.0). Although no literature spectral data were available, experimental spectral data were consistent with that expected for the title compound.

1-(4-Hydroxy-3-methoxyphenyl)-4-methyl-3-pentanone (35)

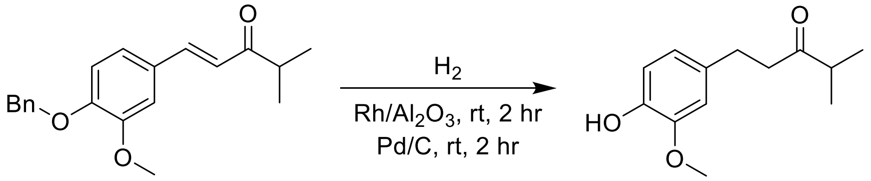

Synthesised using General Method G on a 12.0 mmol scale. White solid (2.41 g, 10.9 mmol, 90.6% yield, mp 29-31 °C, R_f: 0.23 (5:1 (v/v) hexane:ethyl acetate), elemental analysis C: 70.10% H: 8.31% (calc. C: 70.24% H: 8.16%)). ^{1}H NMR (400 MHz, $CDCl_3$): δ 1.06 (6H, d, *J* = 7.0 Hz), 2.56 (1H, sep, *J* = 6.9 Hz), 2.73 (2H, m), 2.82 (2H, m), 3.86 (3H, s), 5.51 (1H, s), 6.66 (1H, dd, *J* = 1.9, 8.0 Hz), 6.69 (1H, d, *J* = 1.8 Hz), 6.82 (1H, d, *J* = 8.0 Hz) ppm. ^{13}C NMR (101 MHz, $CDCl_3$): δ 18.2, 29.7, 41.2, 42.5, 56.0, 111.2, 114.4, 120.9, 133.4, 144.0, 146.5, 214.1 ppm. GC-MS (EI) *m/z* (% of base peak): 222 ($M^{•+}$, 34.6), 179 ($M^{•+}$-$CH(CH_3)_2$, 27.6), 151 ($M^{•+}$-$COCH(CH_3)_2$, 13.1), 147 (4.2), 137 ($M^{•+}$-$CH_2COCH(CH_3)_2$, 100.0), 124 (3.4), 122 (6.4), 119 (13.6), 94 (3.5), 91 (9.7), 77 (4.1), 71 (3.5). Experimental spectral data were consistent with literature data[28].

1-(4-Benzyloxy-3-methoxyphenyl)-4,4-dimethylpent-1-en-3-one

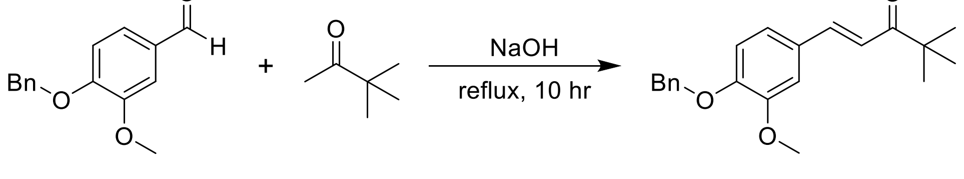

Synthesised using General Method H with 3,3-dimethyl-2-butanone (7.5 mL, 60.0 mmol, 3.0 eq). The reaction time was 10 hours. Pale yellow solid (5.36 g, 16.5 mmol, 82.6% yield, R_f: 0.46 (5:1 (v/v) hexane:ethyl acetate)). ^{1}H NMR (400 MHz, $CDCl_3$): δ 1.23 (9H, s), 3.94 (3H, s), 5.19 (2H, s), 6.88 (1H, d, *J* = 8.1 Hz), 6.98 (1H, d, *J* = 15.5 Hz), 7.10 (2H, m), 7.37 (5H, m), 7.62 (1H, d, *J* = 15.5 Hz) ppm. ^{13}C NMR (101 MHz, $CDCl_3$): δ 26.6, 43.3, 56.2,

71.0, 111.1, 113.7, 118.9, 122.5, 127.3, 128.1, 128.4, 128.8, 136.7, 143.0 149.8, 150.3, 204.3 ppm. GC-MS (EI) *m/z* (% of base peak): 324 ($M^{•+}$, 12.7), 267 ($M^{•+}$-$C(CH_3)_3$, 41.0), 240 (2.6), 233 (2.0), 207 (2.7), 189 (2.2), 177 (2.7), 145 (3.9), 91 ($Bn^{•+}$, 100.0), 89 (2.4), 65 (5.2), 57 (6.9). Although no literature spectral data were available, experimental spectral data were consistent with that expected for the title compound.

1-(4-Hydroxy-3-methoxyphenyl)-4,4-dimethyl-3-pentanone (36)

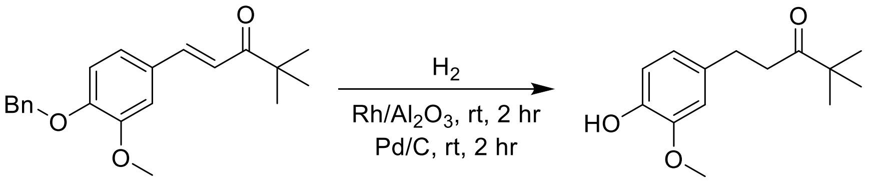

Synthesised using General Method G. White solid (3.51 g, 14.9 mmol, 98.9% yield, mp 62-66 °C (lit. mp 66-68.5 °C[29]), R_f: 0.51 (5:1 (v/v) hexane:ethyl acetate), elemental analysis C: 71.32% H: 8.64% (calc. C: 71.16% H: 8.53%)). ^1H NMR (400 MHz, $CDCl_3$): δ 1.10 (9H, s), 2.78 (4H, m), 3.87 (3H, s), 5.51 (1H, s), 6.67 (1H, dd, *J* = 1.9, 7.9 Hz), 6.69 (1H, d, *J* = 1.8 Hz), 6.82 (1H, d, *J* = 7.9 Hz) ppm. ^{13}C NMR (101 MHz, $CDCl_3$): δ 26.4, 29.9, 38.9, 44.2, 56.0, 111.3, 114.4, 120.9, 133.7, 144.0, 146.5, 215.2 ppm. GC-MS (EI) *m/z* (% of base peak): 236 ($M^{•+}$, 34.0), 179 ($M^{•+}$-$C(CH_3)_3$, 18.8), 152 (3.0), 151 ($M^{•+}$-$COC(CH_3)_3$, 9.1), 137 ($M^{•+}$-$CH_2COC(CH_3)_3$, 100.0), 124 (3.3), 122 (3.9), 119 (6.3), 91 (5.9), 57 (16.9). Experimental spectral data were consistent with literature data[29,30].

2019 Field Trial Compounds

4-(4-(Dimethylamino)-3-methoxyphenyl)-2-butanone (13)

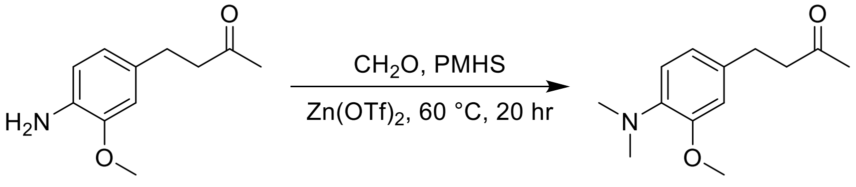

To a yellow solution of 4-(4-amino-3-methoxyphenyl)-2-butanone (2.13 g, 11.0 mmol, 1.0 eq) in THF (11 mL), was added zinc triflate (0.200 g, 0.550 mmol, 0.05 eq), formaldehyde solution (37 wt%) (4.5 mL, 55 mmol, 5.0 eq), and poly(methylhydrosiloxane) (2.2 mL, ~36 mmol of hydride, ~3.3 eq of hydride). The orange solution was stirred at 60 °C for 20 hours, then allowed to cool to room temperature before methanol (5 mL) and sodium hydroxide solution (1.0 mol L^{-1}) (10 mL) were added. Water (50 mL) was added and the crude product was extracted with ethyl acetate (3 × 50 mL) and the combined organic layers were dried with sodium sulfate. The solvent was removed under reduced pressure to yield the crude product as an orange gel, which was purified by flash column chromatography (eluted with 0-30% ethyl acetate in hexane with 1% triethylamine) to give the pure product as a pale yellow oil (0.744 g, 3.36 mmol, 30.6% yield, R_f: 0.15 (3:1 (v/v) hexane:ethyl acetate)). ^1H NMR (400 MHz, $CDCl_3$): δ 2.15 (3H, s), 2.75 (6H, s), 2.75 (2H, m), 2.85 (2H, m), 3.87 (3H, s), 6.68 (1H, d, *J* = 1.8 Hz), 6.71 (1H, dd, *J* = 1.9, 7.9 Hz), 6.86 (1H, d, *J* = 7.9 Hz) ppm. ^{13}C NMR (101 MHz, $CDCl_3$): δ 29.6, 30.2, 43.6, 45.5, 55.4, 111.4, 118.3, 120.2, 135.5, 140.8, 152.5, 208.3 ppm. HRMS (*m/z*): $[M+H]^+$ calc. for $C_{13}H_{20}NO_2$, 222.14886; found, 222.14870. GC-MS (EI) *m/z* (% of base peak): 221 ($M^{•+}$, 50.3), 206 ($M^{•+}$-CH_3, 14.9), 164 ($M^{•+}$-CH_2COCH_3, 100.0), 149 (17.4), 148 (10.4), 134 (13.7), 120 (9.3), 107 (9.7), 91 (11.7), 79

(7.7), 77 (13.0), 65 (10.3). Although no literature spectral data were available, experimental spectral data were consistent with that expected for the title compound.

4-(4-Formamido-3-methoxyphenyl)-2-butanone (14)

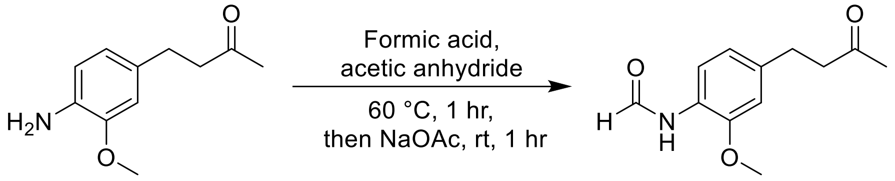

Synthesised using General Method I with 4-(4-amino-3-methoxyphenyl)-2-butanone (3.87 g, 20.0 mmol, 1.0 eq). The product was further purified by recrystallisation from hexane and ethyl acetate. Colourless needles (3.44 g, 15.5 mmol, 77.6% yield, mp 93-94 °C, R_f: 0.05 (3:1 (v/v) hexane:ethyl acetate), elemental analysis C: 65.53% H: 6.78% N: 6.19% (calc. C: 65.14% H: 6.83% N: 6.33%)). Mixture of rotamers in 71:29 ratio. Major rotamer: ^{1}H NMR (400 MHz, $CDCl_3$): δ 2.13 (3H, s), 2.74 (2H, m), 2.85 (2H, m), 3.86 (3H, s), 6.74 (2H, m), 7.78 (1H, br s), 8.22 (1H, d, *J* = 8.1 Hz), 8.41 (1H, d, *J* = 1.4 Hz) ppm. Minor rotamer: ^{1}H NMR (400 MHz, $CDCl_3$): δ 2.13 (3H, s), 2.74 (2H, m), 2.85 (2H, m), 3.84 (3H, s), 6.74 (2H, m), 7.08 (1H, d, *J* = 7.8 Hz), 7.61 (1H, br d, *J* = 10.5 Hz), 8.66 (1H, d, *J* = 11.6 Hz) ppm. Major rotamer: ^{13}C NMR (101 MHz, $CDCl_3$): δ 29.7, 30.2, 45.2, 55.8, 110.5, 120.6, 120.6, 124.9, 137.5, 148.0, 158.8, 208.0 ppm. Minor rotamer: ^{13}C NMR (101 MHz, $CDCl_3$): δ 29.6, 30.2, 45.1, 55.8, 111.7, 117.1, 120.7, 124.3, 138.7, 149.0, 161.6, 207.7 ppm. GC-MS (EI) *m/z* (% of base peak): 221 ($M^{•+}$, 48.7), 193 ($M^{•+}$-CO, 4.9), 178 ($M^{•+}$-$COCH_3$, 14.2), 164 ($M^{•+}$-CH_2COCH_3, 11.4), 150 ($M^{•+}$-CO-$COCH_3$, 17.2), 136 ($M^{•+}$-CO-CH_2COCH_3, 100.0), 135 (8.9), 134 (4.9), 121 (18.4), 106 (5.6), 93 (5.7), 91 (4.8). Although no literature spectral data were available, experimental spectral data were consistent with that expected for the title compound.

***N*-Methyl-4-(4-formamido-3-methoxyphenyl)-2-butanone (15)**

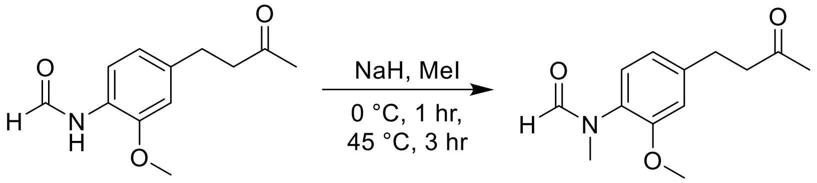

Synthesised using General Method J. Pale brown oil (*impure*, 2.06 g, 8.75 mmol, 87.5% yield, containing approximately 15% *N*-methyl-4-(4-formamido-3-methoxyphenyl)-3-methyl-2-butanone by GC-FID and ^{1}H-NMR, R_f: 0.05 (3:1 (v/v) hexane:ethyl acetate)). ^{1}H NMR (400 MHz, $CDCl_3$): δ 2.16 (3H, s), 2.78 (2H, t, *J* = 7.2 Hz), 2.90 (2H, t, *J* = 7.3 Hz), 3.16 (3H, s), 3.81 (3H, s), 6.77 (2H, m), 7.00 (1H, d, *J* = 7.7 Hz), 8.11 (1H, s) ppm. ^{13}C NMR (101 MHz, $CDCl_3$): δ 29.7, 30.2, 32.9, 45.0, 55.7, 112.4, 120.6, 127.9, 128.9, 142.7, 155.0, 163.7, 207.6 ppm. GC-MS (EI) *m/z* (% of base peak): 235 ($M^{•+}$, 21.2), 207 ($M^{•+}$-CO, 9.0), 192 ($M^{•+}$-$COCH_3$, 8.6), 150 ($M^{•+}$-CO-CH_2COCH_3, 100.0), 135 (19.6), 107 (10.8), 106 (13.1), 91 (13.6), 78 (13.1), 77 (19.3), 65 (12.8), 51 (9.7). Although no literature spectral data were available, experimental spectral data were consistent with that expected for the title compound.

4-(4-Acetamido-3-methoxyphenyl)-2-butanone (16)

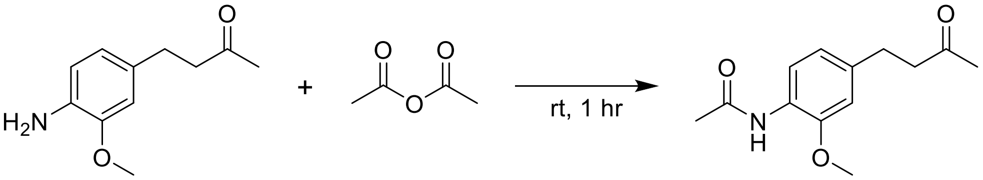

Synthesised using General Method K with acetic anhydride (2.84 mL, 30.0 mmol, 1.5 eq). Pale yellow solid (4.52 g, 19.2 mmol, 96.1% yield, mp 73-76 °C, R_f: 0.05 (3:1 (v/v) hexane:ethyl acetate), elemental analysis C: 66.43% H: 7.43% N: 5.77% (calc. C: 66.36% H: 7.28% N: 5.95%)). ^{1}H NMR (400 MHz, $CDCl_3$): δ 2.13 (3H, s), 2.18 (3H, s), 2.73 (2H, t, *J* = 7.2 Hz), 2.85 (2H, t, *J* = 7.4 Hz), 3.86 (3H, s), 6.70 (1H, d, *J* = 1.6 Hz), 6.75 (1H, dd, *J* = 1.7, 8.2 Hz), 7.67 (1H, br s), 8.22 (1H, d, *J* = 8.2 Hz) ppm. ^{13}C NMR (101 MHz, $CDCl_3$): δ 25.0, 29.8, 30.3, 45.3, 55.8, 110.4, 120.0, 120.6, 125.9, 136.8, 147.8, 168.1, 208.1 ppm. GC-MS (EI) *m/z* (% of base peak): 235 ($M^{•+}$, 38.9), 193 ($M^{•+}$-CH_2CO, 17.4), 192 ($M^{•+}$-$COCH_3$, 7.2), 178 ($M^{•+}$-CH_2COCH_3, 6.2), 150 ($M^{•+}$-CH_2CO-$COCH_3$, 8.5), 136 ($M^{•+}$-CH_2CO-CH_2COCH_3, 100.0), 134 (4.4), 121 (7.6), 106 (4.2), 104 (3.2), 93 (3.3), 91 (3.1). Although no literature spectral data were available, experimental spectral data were consistent with that expected for the title compound.

***N*-Methyl-4-(4-acetamido-3-methoxyphenyl)-2-butanone (17)**

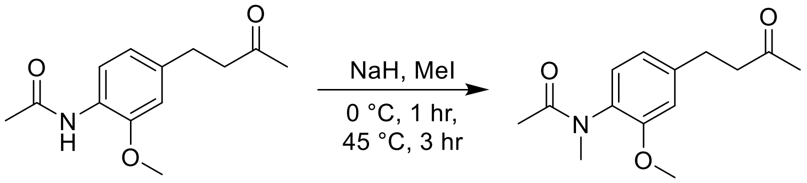

Synthesised using General Method J. Pale yellow solid (*impure*, 1.83 g, 7.34 mmol, 73.4% yield, containing approximately 7% *N*-methyl-4-(4-acetamido-3-methoxyphenyl)-3-methyl-2-butanone and approximately 5% 4-(4-acetamido-3-methoxyphenyl)-2-butanone by GC-FID and ^{1}H-NMR, R_f: 0.04 (3:1 (v/v) hexane:ethyl acetate)). ^{1}H NMR (400 MHz, $CDCl_3$): δ 1.77 (3H, s), 2.16 (3H, s), 2.78 (2H, t, *J* = 7.4 Hz), 2.90 (2H, t, *J* = 7.3 Hz), 3.12 (3H, s), 3.81 (3H, s), 6.77 (2H, m), 7.03 (1H, d, *J* = 7.6 Hz) ppm. ^{13}C NMR (101 MHz, $CDCl_3$): δ 21.8, 29.8, 30.2, 36.1, 45.0, 55.6, 112.3, 120.8, 128.8, 131.2, 142.9, 155.0, 171.6, 207.6 ppm. GC-MS (EI) *m/z* (% of base peak): 249 ($M^{•+}$, 17.3), 218 (10.9), 207 ($M^{•+}$-CH_2CO, 8.5), 150 ($M^{•+}$-CH_2CO-CH_2COCH_3, 78.1), 135 (8.3), 134 (7.8), 106 (7.0), 91 (9.6), 78 (7.1), 77 (11.8), 65 (7.2), 56 (100.0). Although no literature spectral data were available, experimental spectral data were consistent with that expected for the title compound.

4-(4-Trifluoroacetamido-3-methoxyphenyl)-2-butanone (18)

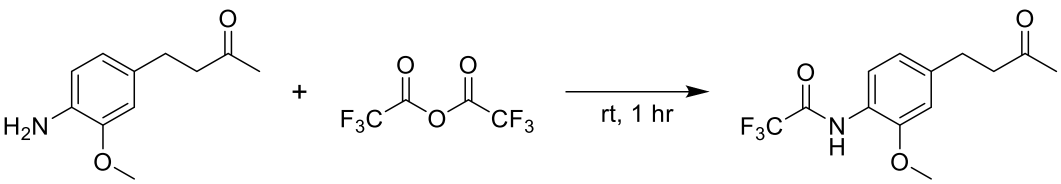

Synthesised using General Method K on a 15.0 mmol scale with trifluoroacetic anhydride (3.13 mL, 22.5 mmol, 1.5 eq) and DCM (40 mL). The solution was washed with water instead of sodium hydrogen carbonate solution. Flash column chromatography was

performed with 0-40% ethyl acetate in hexane. White solid (3.55 g, 12.3 mmol, 81.8% yield, mp 86-87 °C, R_f: 0.27 (3:1 (v/v) hexane:ethyl acetate), elemental analysis C: 54.17% H: 4.88% N: 4.73% (calc. C: 53.98% H: 4.88% N: 4.84%)). ^{1}H NMR (400 MHz, $CDCl_3$): δ 2.14 (3H, s), 2.76 (2H, t, *J* = 7.2 Hz), 2.88 (2H, t, *J* = 7.4 Hz), 3.91 (3H, s), 6.77 (1H, d, *J* = 1.7 Hz), 6.81 (1H, dd, *J* = 1.6, 8.2 Hz), 8.18 (1H, d, *J* = 8.2 Hz), 8.49 (1H, br s) ppm. ^{13}C NMR (101 MHz, $CDCl_3$): δ 29.7, 30.2, 45.1, 56.0, 110.8, 115.9 (q, *J* = 288.5 Hz), 120.3, 120.8, 123.2, 139.5, 148.5, 154.3 (q, *J* = 37.2 Hz), 207.7 ppm. ^{19}F NMR (376 MHz, $CDCl_3$): δ -76.78 ppm. GC-MS (EI) *m/z* (% of base peak): 289 ($M^{•+}$, 100.0), 246 ($M^{•+}$-$COCH_3$, 41.6), 232 ($M^{•+}$-CH_2COCH_3, 56.2), 228 (39.5), 219 (5.5), 162 (9.5), 150 (10.0), 149 (20.9), 148 (5.3), 137 (31.7), 134 (11.3), 91 (5.5). Although no literature spectral data were available, experimental spectral data were consistent with that expected for the title compound.

_N_-Methyl-4-(4-trifluoroacetamido-3-methoxyphenyl)-2-butanone (19)

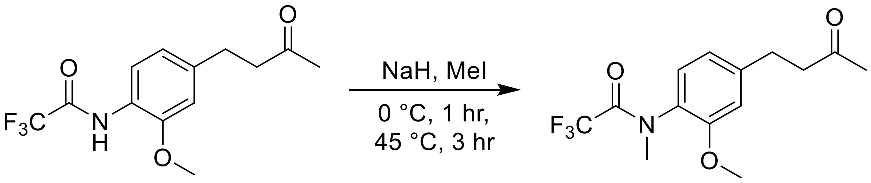

Synthesised using General Method J on an 8.00 mmol scale. Pale brown solid (*impure*, 1.92 g, 6.33 mmol, 79.1% yield, containing approximately 5% 4-(4-trifluoroacetamido-3-methoxyphenyl)-2-butanone by GC-FID and ^{1}H-NMR, R_f: 0.19 (3:1 (v/v) hexane:ethyl acetate)). ^{1}H NMR (400 MHz, $CDCl_3$): δ 2.16 (3H, s), 2.79 (2H, t, *J* = 7.2 Hz), 2.91 (2H, t, *J* = 7.3 Hz), 3.23 (3H, s), 3.82 (3H, s), 6.77 (2H, m), 7.08 (1H, d, *J* = 7.7 Hz) ppm. ^{13}C NMR (101 MHz, $CDCl_3$): δ 29.8, 30.2, 38.1, 44.8, 55.6, 112.0, 116.4 (q, *J* = 287.9 Hz), 120.3, 127.1, 129.1, 144.2, 155.3, 157.7 (q, *J* = 35.3 Hz), 207.6 ppm. ^{19}F NMR (376 MHz, $CDCl_3$): δ -69.93 ppm. GC-MS (EI) *m/z* (% of base peak): 303 ($M^{•+}$, 29.4), 260 ($M^{•+}$-$COCH_3$, 27.3), 246 ($M^{•+}$-CH_2COCH_3, 7.7), 164 (11.3), 163 (54.6), 148 (11.7), 134 (10.2), 110 (100.0), 91 (13.1), 77 (14.9), 69 (10.1), 65 (9.4). Although no literature spectral data were available, experimental spectral data were consistent with that expected for the title compound.

4-Benzyloxy-3-hydroxybenzaldehyde

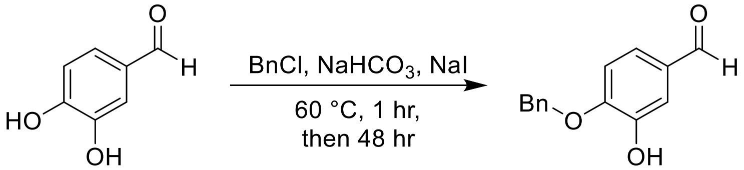

A brown suspension of 3,4-dihydroxybenzaldehyde (16.6 g, 120 mmol, 1.0 eq), sodium hydrogen carbonate (15.1 g, 180 mmol, 1.5 eq), and sodium iodide (8.99 g, 60.0 mmol, 0.5 eq) in DMF (200 mL) was stirred at 60 °C for 1 hour before benzyl chloride (30.4 g, 240 mmol, 2.0 eq) was added. The brown suspension was stirred at 60 °C for a further 48 hours. The volume of the brown suspension was reduced *in vacuo* and water (100 mL) was added. The crude product was extracted with chloroform (3 × 100 mL) and the combined organic layers were dried with sodium sulfate. The solvent was removed under reduced pressure to yield the crude product as a brown liquid, which was purified by flash column chromatography (eluted with 15-35% ethyl acetate in hexane) to give the pure product as a grey solid (16.4 g, 71.6 mmol, 59.7% yield, R_f: 0.29 (4:1 (v/v) hexane:ethyl acetate)). ^{1}H NMR (400 MHz, $CDCl_3$): δ 5.20 (2H, s), 5.86 (1H, s), 7.04 (1H, d, *J* = 8.3 Hz), 7.40 (6H, m), 7.46 (1H, d, *J* = 2.0 Hz), 9.83 (1H, s) ppm. ^{13}C NMR (101 MHz, $CDCl_3$): δ 71.4, 111.7,

114.6, 124.5, 128.0, 128.9, 129.0, 131.0, 135.4, 146.5, 151.1, 191.1 ppm. Experimental spectral data were consistent with literature data[31].

4-(4-Benzyloxy-3-hydroxyphenyl)but-3-en-2-one

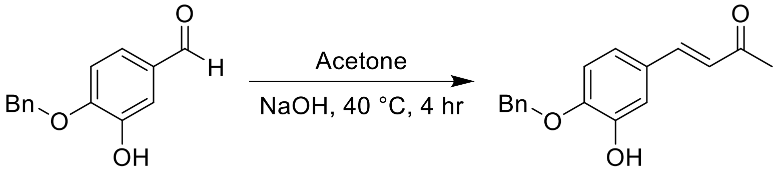

Synthesised using General Method D on a 70.0 mmol scale with 4-benzyloxy-3-hydroxybenzaldehyde (16.0 g, 70.0 mmol, 1.0 eq) and sodium hydroxide solution (5% (w/v)) (62 mL, 78 mmol, 1.1 eq). Water was not added to the reaction but was added after the volume of the mixture was reduced *in vacuo*. The resulting precipitate was collected by vacuum filtration and washed with water to give the crude product as a yellow-brown solid (18.3 g, 68.2 mmol, 97.4% yield, R_f: 0.24 (3:1 (v/v) hexane:ethyl acetate)). ^{1}H NMR (400 MHz, $CDCl_3$): δ 2.35 (3H, s), 5.15 (2H, s), 5.85 (1H, br s), 6.58 (1H, d, *J* = 16.2 Hz), 6.92 (1H, d, *J* = 8.4 Hz), 7.03 (1H, dd, *J* = 2.1, 8.4 Hz), 7.17 (1H, d, *J* = 2.1 Hz), 7.39 (6H, m) ppm. ^{13}C NMR (101 MHz, $CDCl_3$): δ 27.6, 71.3, 112.2, 113.6, 122.1, 125.7, 128.0, 128.4, 128.7, 128.9, 135.8, 143.4, 146.3, 148.1, 198.5 ppm. GC-MS (EI) *m/z* (% of base peak): 268 ($M^{\bullet+}$, 4.7), 226 (3.9), 177 (2.8), 134 (1.2), 91 ($Bn^{\bullet+}$, 100.0), 90 (1.0), 89 (1.7), 78 (1.3), 77 (2.1), 65 (7.8), 63 (1.1), 51 (1.5). Although no literature spectral data were available, experimental spectral data were consistent with that expected for the title compound.

4-(4-Benzyloxy-3-hydroxyphenyl)-2-butanone

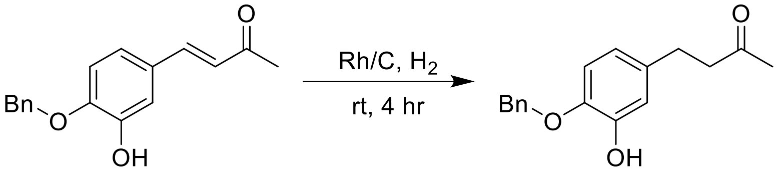

Synthesised using General Method E on a 65.0 mmol scale with 4-(4-benzyloxy-3-hydroxyphenyl)but-3-en-2-one (17.4 g, 65.0 mmol, 1.0 eq) in ethyl acetate (200 mL) and Rh/C (5 wt%, wet support) (2.79 g, 1.0 mol%). The reaction time was 4 hours and the crude product was purified by recrystallisation from aqueous ethanol. Off-white short needles (14.3 g, 52.9 mmol, 81.5% yield, R_f: 0.35 (3:1 (v/v) hexane:ethyl acetate)). ^{1}H NMR (400 MHz, $CDCl_3$): δ 2.13 (3H, s), 2.72 (2H, t, *J* = 7.6 Hz), 2.81 (2H, t, *J* = 7.6 Hz), 5.08 (2H, s), 5.66 (1H, s), 6.64 (1H, dd, *J* = 2.1, 8.2 Hz), 6.78 (1H, d, *J* = 2.1 Hz), 6.83 (1H, d, *J* = 8.2 Hz), 7.38 (5H, m) ppm. ^{13}C NMR (101 MHz, $CDCl_3$): δ 29.3, 30.2, 45.4, 71.4, 112.4, 114.8, 119.8, 127.9, 128.5, 128.8, 134.8, 136.6, 144.3, 146.0, 208.2 ppm. GC-MS (EI) *m/z* (% of base peak): 270 ($M^{\bullet+}$, 5.8), 252 (6.6), 237 (1.1), 179 (1.0), 137 (2.2), 119 (1.2), 91 ($Bn^{\bullet+}$, 100.0), 89 (1.2), 79 (1.1), 77 (1.4), 65 (7.1), 51 (0.9). Although no literature spectral data were available, experimental spectral data were consistent with that expected for the title compound.

4-(3,4-Dihydroxyphenyl)-2-butanone (21)

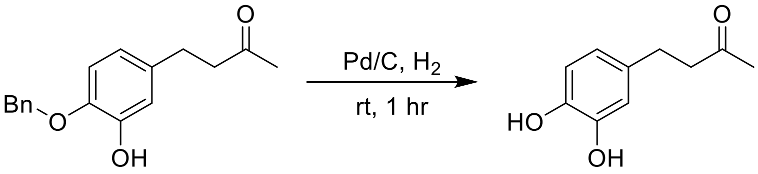

Synthesised using General Method L. Flash column chromatography was performed with 0-50% ethyl acetate in hexane. White solid (1.60 g, 8.86 mmol, 88.6% yield, mp 86-87 °C (lit. mp 85.5-86 °C[20] and 85.5-85.8 °C[32]), R_f: 0.12 (3:1 (v/v) hexane:ethyl acetate), elemental analysis C: 66.62% H: 6.72% (calc. C: 66.65% H: 6.71%)). ^{1}H NMR (400 MHz, $CDCl_3$): δ 2.14 (3H, s), 2.76 (4H, m), 5.80 (1H, br s), 6.06 (1H, br s), 6.58 (1H, dd, *J* = 2.1, 8.1 Hz), 6.70 (1H, d, *J* = 2.0 Hz), 6.76 (1H, d, *J* = 8.0 Hz) ppm. ^{13}C NMR (101 MHz, $CDCl_3$): δ 29.2, 30.3, 45.5, 115.5, 115.5, 120.6, 133.8, 142.1, 143.8, 209.9 ppm. GC-MS (EI) *m/z* (% of base peak): 180 ($M^{\bullet+}$, 35.0), 137 ($M^{\bullet+}$-$COCH_3$, 20.9), 123 ($M^{\bullet+}$-CH_2COCH_3, 100.0), 119 (18.0), 110 (16.2), 91 (53.5), 77 (38.0), 65 (26.8), 63 (14.3), 55 (22.0), 53 (18.7), 51 (41.7). Experimental spectral data were consistent with that expected for the title compound and with literature data[33].

4-(4-Hydroxy-3-nitrophenyl)-2-butanone (22)

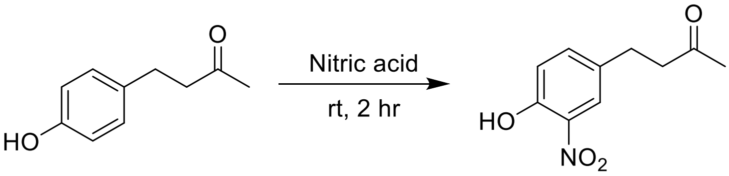

To a white suspension of raspberry ketone (8.21 g, 50.0 mmol, 1.0 eq) in water (40 mL) at 0 °C, was added concentrated nitric acid (7.5 mL, ~110 mmol, ~2.2 eq) dropwise. The yellow suspension was then allowed to warm to room temperature. The dark red suspension was stirred at room temperature for 2 hours before water (100 mL) was added and the crude product was extracted with DCM (3 × 100 mL). The combined organic layers were dried with sodium sulfate and the solvent was removed under reduced pressure to yield the crude product as an orange solid, which was purified by flash column chromatography (eluted with 0-30% ethyl acetate in hexane) to give the pure product as a yellow solid (9.19 g, 43.9 mmol, 87.9% yield, mp 63-66 °C, R_f: 0.41 (3:1 (v/v) hexane:ethyl acetate), elemental analysis C: 57.47% H: 5.22% N: 6.58% (calc. C: 57.41% H: 5.30% N: 6.70%)). ^{1}H NMR (400 MHz, $CDCl_3$): δ 2.15 (3H, s), 2.77 (2H, t, *J* = 7.2 Hz), 2.88 (2H, t, *J* = 7.2 Hz), 7.07 (1H, d, *J* = 8.6 Hz), 7.43 (1H, dd, *J* = 2.2, 8.6 Hz), 7.90 (1H, d, *J* = 2.0 Hz), 10.44 (1H, br s) ppm. ^{13}C NMR (101 MHz, $CDCl_3$): δ 28.3, 30.2, 44.5, 120.1, 124.0, 133.4, 133.6, 138.3, 153.7, 207.1 ppm. GC-MS (EI) *m/z* (% of base peak): 209 ($M^{\bullet+}$, 5.0), 191 (6.2), 152 ($M^{\bullet+}$-CH_2COCH_3, 37.8), 149 (100.0), 132 (11.6), 120 (10.0), 119 (8.5), 106 (14.0), 105 (8.9), 91 (13.1), 77 (9.5), 65 (7.2). Although no literature spectral data were available, experimental spectral data were consistent with that expected for the title compound.

4-(4-Benzyloxy-3-formoxyphenyl)-2-butanone

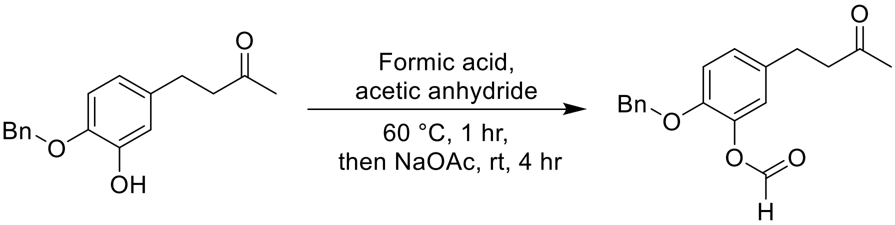

Synthesised using General Method I on a 10.0 mmol scale with 4-(4-benzyloxy-3-hydroxyphenyl)-2-butanone (2.70 g, 10.0 mmol, 1.0 eq). The reaction time was 4 hours. Water (40 mL) was added to the reaction and the crude product was extracted with ethyl acetate (3 × 40 mL). The crude product was not purified. Brown oil (3.14 g, 10.0 mmol, quantitative, R_f: 0.37 (3:1 (v/v) hexane:ethyl acetate)). ^{1}H NMR (400 MHz, $CDCl_3$): δ 2.14 (3H, s), 2.73 (2H, t, *J* = 7.4 Hz), 2.84 (2H, t, *J* = 7.3 Hz), 5.07 (2H, s), 6.94 (2H, m), 7.01 (1H, dd, *J* = 2.0, 8.5 Hz), 7.33 (5H, m), 8.26 (1H, s) ppm. ^{13}C NMR (101 MHz, $CDCl_3$): δ 28.8, 30.2, 45.1, 71.0, 114.4, 122.6, 127.1, 127.4, 128.2, 128.7, 134.5, 136.6, 139.2, 148.4, 159.3, 207.9 ppm. Although no literature spectral data were available, experimental spectral data were consistent with that expected for the title compound.

4-(4-Hydroxy-3-formoxyphenyl)-2-butanone and 4-(3-Hydroxy-4-formoxyphenyl)-2-butanone

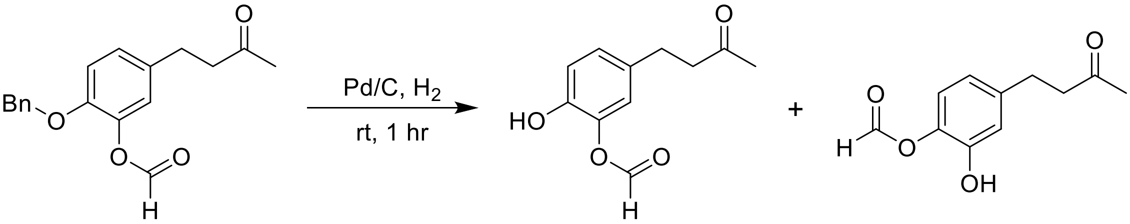

Synthesised using General Method L. Flash column chromatography was performed with 0-50% ethyl acetate in hexane to give a mixture of products as a white solid (2.00 g, 9.63 mmol, 96.3% yield).

4-(3,4-Diformoxyphenyl)-2-butanone (23)

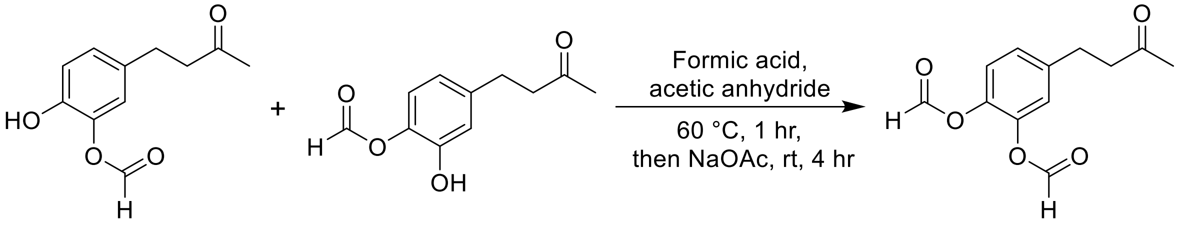

Synthesised using General Method I on an 8.89 mmol scale with a mixture of 4-(4-hydroxy-3-formoxyphenyl)-2-butanone and 4-(3-hydroxy-4-formoxyphenyl)-2-butanone (1.85 g, 8.89 mmol, 1.0 eq). The reaction time was 4 hours. Water (30 mL) was added to the reaction and the crude product was extracted with ethyl acetate (3 × 30 mL). Flash column chromatography was performed with 0-30% ethyl acetate in hexane. Pale yellow oil (1.99 g, 8.44 mmol, 95.0% yield, R_f: 0.18 (3:1 (v/v) hexane:ethyl acetate)). ^{1}H NMR (400 MHz, $CDCl_3$): δ 2.15 (3H, s), 2.77 (2H, t, *J* = 7.3 Hz), 2.91 (2H, t, *J* = 7.4 Hz), 7.08 (1H, d, *J* = 1.3 Hz), 7.14 (2H, m), 8.22 (1H, s), 8.22 (1H, s) ppm. ^{13}C NMR (101 MHz, $CDCl_3$): δ 29.0, 30.2, 44.7, 123.2, 127.4, 139.4, 141.0, 141.1, 158.2, 158.3, 207.2 ppm. HRMS (*m*/*z*):

$[M+H]^+$ calc. for $C_{12}H_{13}O_5$, 237.07576; found, 237.07570. GC-MS (EI) *m/z* (% of base peak): 236 ($M^{•+}$, 0.9), 180 ($M^{•+}$-CO-CO, 20.2), 137 ($M^{•+}$-CO-CO-$COCH_3$, 29.1), 123 ($M^{•+}$-CO-CO-CH_2COCH_3, 100.0), 119 (16.0), 110 (21.2), 91 (45.3), 77 (35.0), 65 (26.5), 55 (21.0), 53 (18.6), 51 (39.0). Although no literature spectral data were available, experimental spectral data were consistent with that expected for the title compound.

4-(4-Benzyloxy-3-propoxyphenyl)-2-butanone

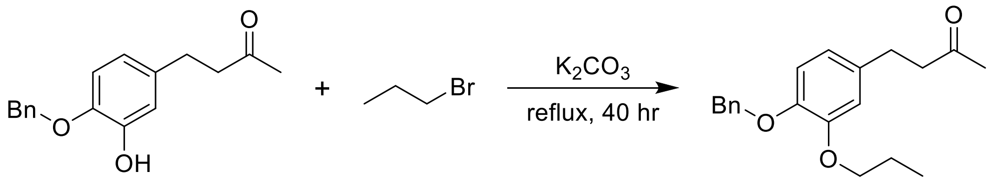

Synthesised using General Method M with 1-bromopropane (1.82 mL, 20.0 mmol, 2.0 eq). Crude pale brown solid (3.14 g, 10.0 mmol, quantitative, R_f: 0.41 (4:1 (v/v) hexane:ethyl acetate)). ^1H NMR (400 MHz, $CDCl_3$): δ 1.06 (3H, t, *J* = 7.4 Hz), 1.85 (2H, m), 2.13 (3H, s), 2.73 (2H, t, *J* = 7.7 Hz), 2.82 (2H, t, *J* = 7.7 Hz), 3.98 (2H, t, *J* = 6.6 Hz), 5.10 (2H, s), 6.65 (1H, dd, *J* = 2.1, 8.1 Hz), 6.75 (1H, d, *J* = 2.0 Hz), 6.82 (1H, d, *J* = 8.1 Hz), 7.36 (5H, m) ppm. ^{13}C NMR (101 MHz, $CDCl_3$): δ 10.7, 22.8, 29.5, 30.3, 45.5, 70.8, 71.7, 114.4, 115.7, 120.4, 127.4, 127.8, 128.5, 134.7, 137.8, 147.1, 149.6, 208.3 ppm. GC-MS (EI) *m/z* (% of base peak): 312 ($M^{•+}$, 50.3), 270 ($M^{•+}$-C_3H_6, 5.9), 221 (11.1), 179 ($M^{•+}$-C_3H_6-Bn, 31.3), 163 (2.6), 137 (82.0), 119 (16.9), 105 (2.3), 91 ($Bn^{•+}$, 100.0), 79 (3.0), 77 (3.6), 65 (9.7). Although no literature spectral data were available, experimental spectral data were consistent with that expected for the title compound.

4-(4-Hydroxy-3-propoxyphenyl)-2-butanone (26)

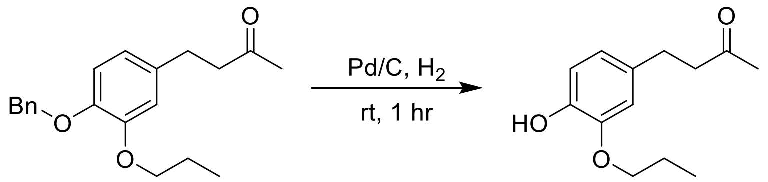

Synthesised using General Method L. White solid (1.84 g, 8.29 mmol, 82.9% yield, mp 37-38 °C (lit. mp 36-37 °C[20]), R_f: 0.40 (3:1 (v/v) hexane:ethyl acetate), elemental analysis C: 70.38% H: 8.30% (calc. C: 70.24% H: 8.16%)). ^1H NMR (400 MHz, $CDCl_3$): δ 1.04 (3H, t, *J* = 7.4 Hz), 1.83 (2H, m), 2.13 (3H, s), 2.72 (2H, t, *J* = 7.5 Hz), 2.81 (2H, t, *J* = 7.6 Hz), 3.98 (2H, t, *J* = 6.6 Hz), 5.58 (1H, s), 6.65 (1H, dd, *J* = 1.9, 8.0 Hz), 6.68 (1H, d, *J* = 1.9 Hz), 6.82 (1H, d, *J* = 8.0 Hz) ppm. ^{13}C NMR (101 MHz, $CDCl_3$): δ 10.6, 22.7, 29.6, 30.2, 45.7, 70.5, 112.1, 114.4, 120.7, 132.9, 144.2, 145.9, 208.4 ppm. GC-MS (EI) *m/z* (% of base peak): 222 ($M^{•+}$, 60.0), 180 ($M^{•+}$-C_3H_6, 29.9), 165 ($M^{•+}$-CH_2COCH_3, 11.0), 162 (17.6), 147 (6.4), 137 ($M^{•+}$-C_3H_6-$COCH_3$, 63.5), 123 ($M^{•+}$-C_3H_6-CH_2COCH_3, 100.0), 122 (7.0), 119 (14.3), 110 (18.0), 91 (11.7), 77 (5.3). Although no literature spectral data were available, experimental spectral data were consistent with that expected for the title compound.

4-(4-Benzyloxy-3-isopropoxyphenyl)-2-butanone

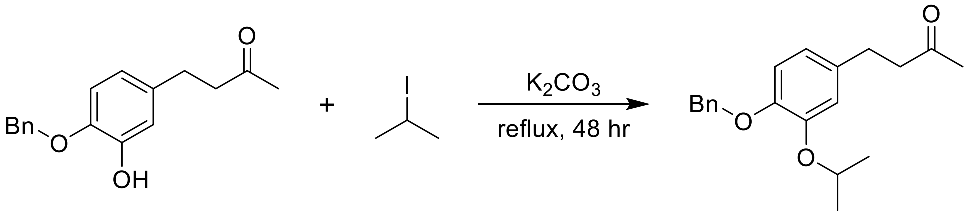

Synthesised using General Method M with 2-iodopropane (2.00 mL, 20.0 mmol, 2.0 eq). The reaction time was 48 hours. Crude brown liquid (3.09 g, 9.90 mmol, 99.0% yield, R_f: 0.41 (4:1 (v/v) hexane:ethyl acetate)). ^{1}H NMR (400 MHz, $CDCl_3$): δ 1.35 (6H, d, *J* = 6.1 Hz), 2.13 (3H, s), 2.72 (2H, t, *J* = 7.6 Hz), 2.81 (2H, t, *J* = 7.6 Hz), 4.51 (1H, sep, *J* = 6.1 Hz), 5.08 (2H, s), 6.68 (1H, dd, *J* = 2.1, 8.2 Hz), 6.77 (1H, d, *J* = 2.1 Hz), 6.83 (1H, d, J = 8.2 Hz), 7.36 (5H, m) ppm. ^{13}C NMR (101 MHz, $CDCl_3$): δ 22.4, 29.4, 30.3, 45.5, 71.6, 72.2, 115.9, 118.1, 121.3, 127.4, 127.8, 128.5, 134.6, 137.8, 148.2, 148.4, 208.3 ppm. GC-MS (EI) *m/z* (% of base peak): 312 ($M^{\bullet+}$, 29.5), 270 ($M^{\bullet+}$-C_3H_6, 18.6), 253 (2.5), 252 (11.1), 221 (2.0), 192 (2.0), 179 ($M^{\bullet+}$-C_3H_6-Bn, 25.0), 137 (47.7), 123 (2.1), 119 (9.6), 91 ($Bn^{\bullet+}$, 100.0), 65 (4.7). Although no literature spectral data were available, experimental spectral data were consistent with that expected for the title compound.

4-(4-Hydroxy-3-isopropoxyphenyl)-2-butanone (27)

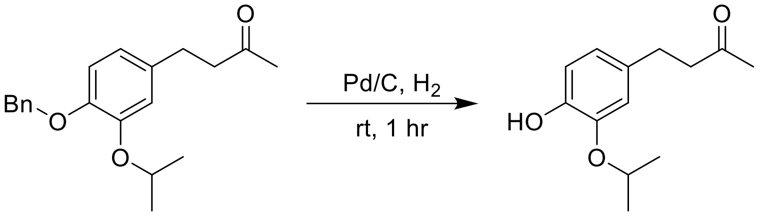

Synthesised using General Method L on a 9.90 mmol scale. White solid (1.84 g, 8.28 mmol, 83.7% yield, mp 33-34 °C, R_f: 0.48 (3:1 (v/v) hexane:ethyl acetate), elemental analysis C: 70.34% H: 8.33% (calc. C: 70.24% H: 8.16%)). ^{1}H NMR (400 MHz, $CDCl_3$): δ 1.35 (6H, d, *J* = 6.1 Hz), 2.12 (3H, s), 2.71 (2H, t, *J* = 7.5 Hz), 2.80 (2H, t, *J* = 7.5 Hz), 4.55 (1H, sep, *J* = 6.1 Hz), 5.61 (1H, s), 6.64 (1H, dd, *J* = 1.8, 8.1 Hz), 6.70 (1H, d, *J* = 1.8 Hz), 6.82 (1H, d, *J* = 8.0 Hz) ppm. ^{13}C NMR (101 MHz, $CDCl_3$): δ 22.3, 29.6, 30.3, 45.7, 71.7, 113.8, 114.5, 120.9, 132.9, 144.6, 145.0, 208.4 ppm. GC-MS (EI) *m/z* (% of base peak): 222 ($M^{\bullet+}$, 35.6), 180 ($M^{\bullet+}$-C_3H_6, 55.1), 163 (5.0), 162 (17.3), 147 (7.6), 137 ($M^{\bullet+}$-C_3H_6-$COCH_3$, 62.8), 123 ($M^{\bullet+}$-C_3H_6-CH_2COCH_3, 100.0), 122 (6.6), 119 (15.1), 110 (18.4), 91 (14.6), 77 (6.5). Although no literature spectral data were available, experimental spectral data were consistent with that expected for the title compound.

4-(4-Benzyloxy-3-(*tert*-butoxy)phenyl)-2-butanone

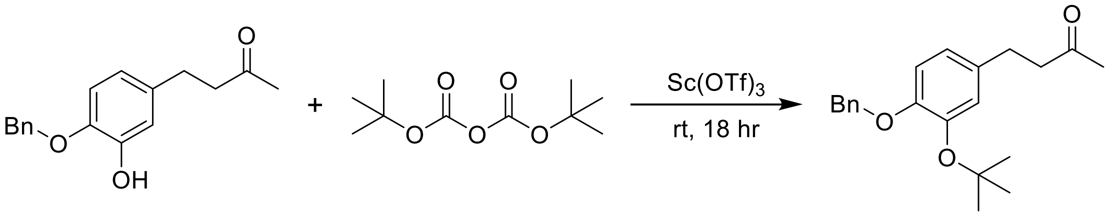

To a pale brown suspension of 4-(4-benzyloxy-3-hydroxyphenyl)-2-butanone (1.62 g, 6.00 mmol, 1.0 eq) in DCM (9 mL), was added scandium(III) triflate (0.148 g, 0.300 mmol, 5.0 mol%) followed by di-*tert*-butyl dicarbonate (4.58 g, 21.0 mmol, 3.5 eq). The brown suspension was stirred at room temperature for 18 hours. Water (20 mL) was added and the crude product was extracted with DCM (3 × 20 mL). The combined organic layers were dried with sodium sulfate and the solvent removed under reduced pressure to yield the crude product as a grey partially solidified liquid, which was purified by flash column chromatography (eluted with 0-30% ethyl acetate in hexane) to give the pure product as a colourless liquid (1.15 g, 3.52 mmol, 58.7% yield, R_f: 0.45 (4:1 (v/v) hexane:ethyl acetate)) and recovered starting material as a white solid (0.607 g, 2.25 mmol, 37.5% yield). Title product: ^{1}H NMR (400 MHz, $CDCl_3$): δ 1.36 (9H, s), 2.12 (3H, s), 2.71 (2H, t, *J* = 7.7 Hz), 2.81 (2H, t, *J* = 7.7 Hz), 5.05 (2H, s), 6.81 (1H, dd, *J* = 2.1, 8.3 Hz), 6.85 (2H, m), 7.37 (5H, m) ppm. ^{13}C NMR (101 MHz, $CDCl_3$): δ 28.9, 29.2, 30.3, 45.5, 71.3, 80.0, 115.3, 123.9, 126.0, 127.4, 127.8, 128.5, 133.9, 137.7, 145.3, 151.8, 208.3 ppm. HRMS (*m*/*z*): $[M]^+$ calc. for $C_{21}H_{26}O_3$, 326.18765; found, 326.18754. GC-MS (EI) *m/z* (% of base peak): 326 ($M^{\bullet+}$, 0.7), 270 ($M^{\bullet+}$-C_4H_8, 21.6), 253 (5.2), 252 (17.7), 237 (1.8), 195 (1.6), 179 ($M^{\bullet+}$-C_4H_8-Bn, 2.0), 137 (4.4), 119 (1.6), 91 ($Bn^{\bullet+}$, 100.0), 65 (2.7), 57 (2.7). Although no literature spectral data were available, experimental spectral data were consistent with that expected for the title compound.

4-(4-Hydroxy-3-(*tert*-butoxy)phenyl)-2-butanone (28)

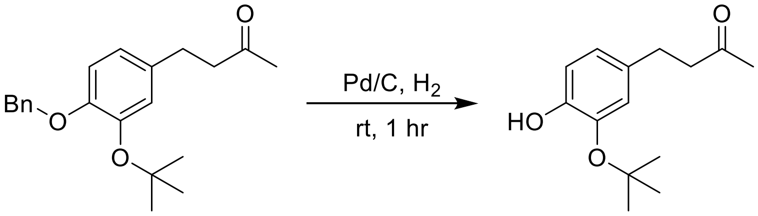

Synthesised using General Method L on a 7.00 mmol scale. Colourless oil (1.59 g, 6.72 mmol, 96.0% yield, R_f: 0.37 (4:1 (v/v) hexane:ethyl acetate)). ^{1}H NMR (400 MHz, $CDCl_3$): δ 1.39 (9H, s), 2.11 (3H, s), 2.69 (2H, t, *J* = 7.3 Hz), 2.78 (2H, t, *J* = 7.3 Hz), 5.68 (1H, s), 6.75 (1H, dd, *J* = 2.0, 8.2 Hz), 6.83 (2H, m) ppm. ^{13}C NMR (101 MHz, $CDCl_3$): δ 29.0, 29.4, 30.2, 45.6, 80.6, 114.8, 122.4, 123.8, 132.4, 142.1, 148.1, 208.4 ppm. HRMS (*m*/*z*): $[M+Na]^+$ calc. for $C_{14}H_{20}O_3Na$, 259.13047; found, 259.13036. GC-MS (EI) *m/z* (% of base peak): 236 ($M^{\bullet+}$, 1.7), 180 ($M^{\bullet+}$-C_4H_8, 62.6), 137 ($M^{\bullet+}$-C_4H_8-$COCH_3$, 43.8), 123 ($M^{\bullet+}$-C_4H_8-CH_2COCH_3, 100.0), 119 (11.6), 110 (24.9), 91 (24.8), 77 (17.5), 65 (12.2), 57 (96.7), 56 (12.6), 55 (15.6). Although no literature spectral data were available, experimental spectral data were consistent with that expected for the title compound.

Ethyl 3-(4-benzyloxy-3-methoxyphenyl)acrylate

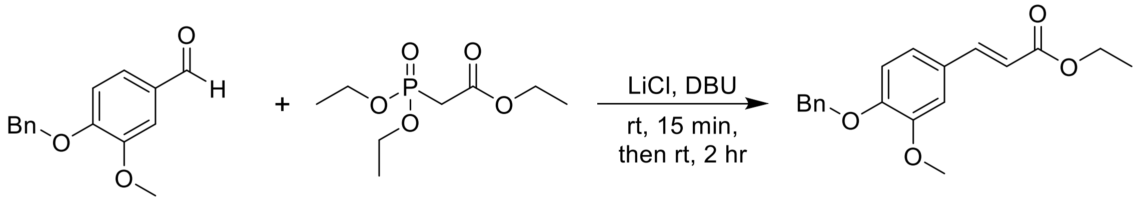

To a colourless solution of lithium chloride (1.02 g, 24.1 mmol, 1.2 eq) in THF (48 mL), was added triethyl phosphonoacetate (4.80 mL, 24.2 mmol, 1.2 eq). The colourless solution was stirred at room temperature for 15 minutes before DBU (3.30 mL, 22.1 mmol, 1.1 eq) and 4-benzyloxy-3-methoxybenzaldehyde (4.85 g, 20.0 mmol, 1.0 eq) were added. The yellow mixture was stirred at room temperature for 2 hours. Water (50 mL) was added and the crude product was extracted with ethyl acetate (3 × 50 mL) and the combined organic layers were dried with sodium sulfate. The solvent was removed under reduced pressure to yield the crude product as a pale orange solid (6.81 g, 20.0 mmol, quantitative yield, R_f: 0.39 (5:1 (v/v) hexane:ethyl acetate)). ^{1}H NMR (400 MHz, $CDCl_3$): δ 1.33 (3H, t, *J* = 7.1 Hz), 3.91 (3H, s), 4.25 (2H, q, *J* = 7.1 Hz), 5.18 (2H, s), 6.30 (1H, d, *J* = 15.9 Hz), 6.86 (1H, d, *J* = 8.3 Hz), 7.03 (1H, dd, *J* = 2.0, 8.3 Hz), 7.07 (1H, d, *J* = 2.0 Hz), 7.36 (5H, m), 7.61 (1H, d, *J* = 15.9 Hz) ppm. ^{13}C NMR (101 MHz, $CDCl_3$): δ 14.5, 56.1, 60.5, 71.0, 110.3, 113.5, 116.2, 122.5, 127.3, 127.9, 128.1, 128.8, 136.7, 144.6, 149.9, 150.3, 167.3 ppm. Experimental spectral data were consistent with literature data[34].

Ethyl 3-(4-benzyloxy-3-methoxyphenyl)propionate

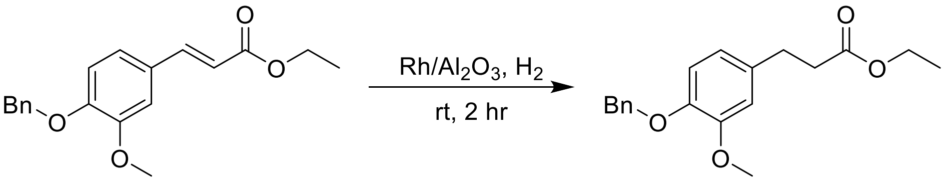

Synthesised using General Method E on a 20.0 mmol scale with ethyl 3-(4-benzyloxy-3-methoxyphenyl)acrylate (6.81 g, 20.0 mmol, 1.0 eq) in ethyl acetate (100 mL) and Rh/Al_2O_3 (0.5 wt%) (4.12 g, 1.00 mol%). The reaction time was 2 hours and flash column chromatography was performed with 0-20% ethyl acetate in hexane to give the title product as a white solid (3.40 g, 10.8 mmol, 54.0% yield, mp 31-32 °C, R_f: 0.42 (5:1 (v/v) hexane:ethyl acetate)) and the deprotected product (ethyl 3-(4-hydroxy-3-methoxyphenyl)propionate) as a white solid (1.81 g, 8.08 mmol, 40.4% yield, mp 41-43 °C (lit. mp 42 °C[35]), R_f: 0.27 (5:1 (v/v) hexane:ethyl acetate)). Title product: ^{1}H NMR (400 MHz, $CDCl_3$): δ 1.23 (3H, t, *J* = 7.1 Hz), 2.59 (2H, t, *J* = 7.8 Hz), 2.89 (2H, t, *J* = 7.8 Hz), 3.87 (3H, s), 4.13 (2H, q, *J* = 7.1 Hz), 5.12 (2H, s), 6.67 (1H, dd, *J* = 2.0, 8.1 Hz), 6.76 (1H, d, *J* = 2.0 Hz), 6.80 (1H, d, *J* = 8.2 Hz), 7.36 (5H, m) ppm. ^{13}C NMR (101 MHz, $CDCl_3$): δ 14.4, 30.8, 36.3, 56.1, 60.5, 71.3, 112.4, 114.4, 120.3, 127.4, 127.9, 128.6, 134.0, 137.5, 146.7, 149.7, 173.1 ppm. Experimental spectral data were consistent with literature data.[36] Deprotected product, ethyl 3-(4-hydroxy-3-methoxyphenyl)propionate: ^{1}H NMR (400 MHz, $CDCl_3$): δ 1.24 (3H, t, *J* = 7.1 Hz), 2.58 (2H, t, *J* = 7.8 Hz), 2.88 (2H, t, *J* = 7.8 Hz), 3.86 (3H, s), 4.12 (2H, q, *J* = 7.1 Hz), 5.55 (1H, s), 6.69 (2H, m), 6.82 (1H, d, *J* = 7.9 Hz) ppm. ^{13}C NMR (101 MHz, $CDCl_3$): δ 14.3, 30.8, 36.5, 56.0, 60.5, 111.1, 114.4, 121.0, 132.6, 144.1, 146.5, 173.1 ppm. Experimental spectral data were consistent with literature data[36].

3-(4-Benzyloxy-3-methoxyphenyl)propionaldehyde

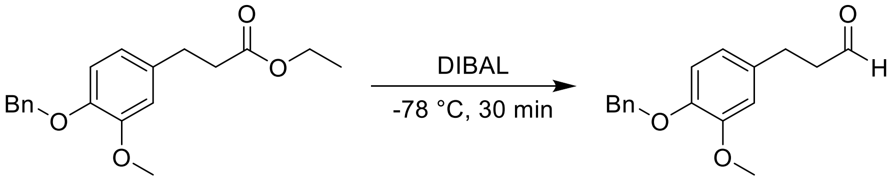

To a colourless solution of ethyl 3-(4-benzyloxy-3-methoxyphenyl)propionate (3.14 g, 10.0 mmol, 1.0 eq) in dry DCM (100 mL) under an argon atmosphere at -78 °C, was slowly added a solution of DIBAL in toluene (1 mol L^{-1}) (13 mL, 13 mmol, 1.3 eq) over 20 minutes. The slightly cloudy white mixture was stirred at -78 °C for 30 minutes before being quenched at -78 °C by the slow addition of a saturated solution of potassium sodium tartrate (60 mL). The mixture was then allowed to warm to room temperature and was stirred for 2 hours. The organic layer was separated and the aqueous layer was washed with DCM (2 × 60 mL). The combined organic layers were dried with sodium sulfate and the solvent removed under reduced pressure to yield the crude product as a white solid (2.64 g, 9.76 mmol, 97.6% yield, R_f: 0.23 (5:1 (v/v) hexane:ethyl acetate)). ^{1}H NMR (400 MHz, $CDCl_3$): δ 2.75 (2H, m), 2.90 (2H, t, *J* = 7.4 Hz), 3.88 (3H, s), 5.12 (2H, s), 6.66 (1H, dd, *J* = 2.1, 8.1 Hz), 6.74 (1H, d, *J* = 2.0 Hz), 6.81 (1H, d, *J* = 8.2 Hz), 7.36 (5H, m), 9.81 (1H, t, *J* = 1.4 Hz) ppm. ^{13}C NMR (101 MHz, $CDCl_3$): δ 27.9, 45.6, 56.1, 71.3, 112.4, 114.4, 120.2, 127.4, 127.9, 128.6, 133.7, 137.4, 146.8, 149.8, 201.8 ppm. Experimental spectral data were consistent with literature data[36].

3-(4-Hydroxy-3-methoxyphenyl)propionaldehyde (33)

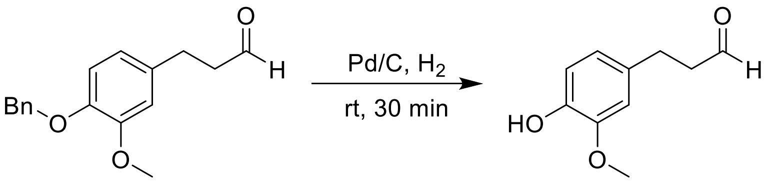

Synthesised using General Method L on a 9.76 mmol scale with a reaction time of 30 minutes. Flash column chromatography was performed with 0-40% ethyl acetate in hexane. Colourless oil (1.08 g, 5.99 mmol, 61.4% yield, R_f: 0.29 (3:1 (v/v) hexane:ethyl acetate)). ^{1}H NMR (400 MHz, $CDCl_3$): δ 2.75 (2H, m), 2.89 (2H, t, *J* = 7.4 Hz), 3.87 (3H, s), 5.52 (1H, br s), 6.68 (2H, m), 6.84 (1H, d, *J* = 7.8 Hz), 9.81 (1H, t, *J* = 1.4 Hz) ppm. ^{13}C NMR (101 MHz, $CDCl_3$): δ 27.9, 45.6, 55.9, 111.0, 114.4, 120.8, 132.2, 144.1, 146.5, 201.8 ppm. HRMS (*m*/*z*): $[M]^+$ calc. for $C_{10}H_{12}O_3$, 180.07810; found, 180.07800. GC-MS (EI) *m/z* (% of base peak): 180 ($M^{\bullet+}$, 61.0), 152 (13.8), 137 ($M^{\bullet+}$-CH_2CHO, 100.0), 124 (33.4), 122 (18.4), 119 (7.8), 109 (9.6), 94 (6.5), 91 (11.6), 79 (5.0), 77 (7.8), 65 (4.7). Experimental spectral data were consistent with that expected for the title compound and with literature data[37].

4-(4-Benzyloxy-3-methoxyphenyl)-2-butanone

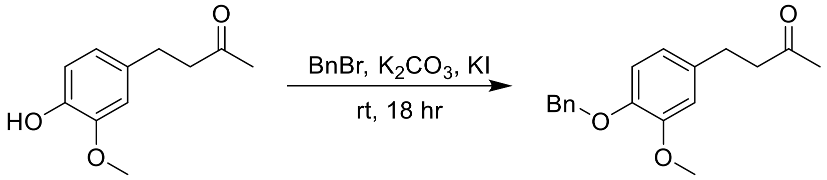

Synthesised using General Method F on a 50.0 mmol scale with zingerone (9.71 g, 50.0 mmol, 1.0 eq) and acetone (60 mL). At the end of the reaction, the white suspension was then diluted with water (100 mL) and the crude product extracted with ethyl acetate (3 × 100 mL). The crude product was recrystallised from hexane. Colourless needles (12.3 g, 43.1 mmol, 86.3% yield, mp 64-66 °C (lit. mp 64 °C[38]), R_f: 0.28 (4:1 (v/v) hexane:ethyl acetate), elemental analysis C: 75.95% H: 7.13% (calc. C: 76.03% H: 7.09%)). ^{1}H NMR (400 MHz, $CDCl_3$): δ 2.13 (3H, s), 2.73 (2H, t, *J* = 7.4 Hz), 2.83 (2H, t, *J* = 7.4 Hz), 3.87 (3H, s), 5.12 (2H, s), 6.64 (1H, dd, *J* = 2.0, 8.1 Hz), 6.74 (1H, d, *J* = 2.0 Hz), 6.79 (1H, d, *J* = 8.2 Hz), 7.36 (5H, m) ppm. ^{13}C NMR (101 MHz, $CDCl_3$): δ 29.5, 30.3, 45.5, 56.1, 71.3, 112.4, 114.4, 120.2, 127.4, 127.9, 128.6, 134.4, 137.5, 146.7, 149.7, 208.2 ppm. GC-MS (EI) *m/z* (% of base peak): 284 ($M^{\bullet+}$, 51.0), 227 ($M^{\bullet+}$-CH_2COCH_3, 6.0), 194 (6.0), 193 ($M^{\bullet+}$-Bn, 15.1), 161 (8.0), 137 (8.2), 133 (15.2), 119 (23.4), 105 (4.2), 91 ($Bn^{\bullet+}$, 100.0), 77 (3.5), 65 (7.5). Although no literature spectral data were available, experimental spectral data were consistent with that expected for the title compound.

3-(4-Benzyloxy-3-methoxybenzyl)but-3-en-2-one

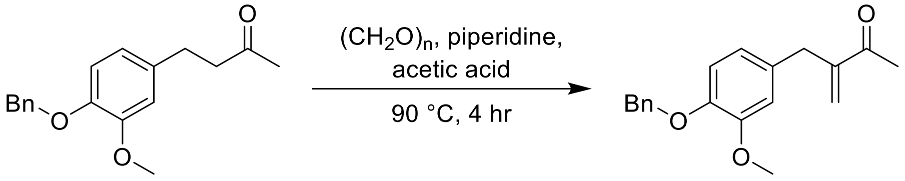

To a colourless solution of 4-(4-benzyloxy-3-methoxyphenyl)-2-butanone (4.27 g, 15.0 mmol, 1.0 eq) in DMF (30 mL) in a pressure tube, was added piperidine (0.74 mL, 7.5 mmol, 0.5 eq), acetic acid (0.74 mL, 13 mmol, 0.9 eq), and paraformaldehyde (2.25 g, 75.0 mmol, 5.0 eq). The white suspension was stirred at 90 °C for 4 hours. The yellow suspension was allowed to cool to room temperature and water (200 mL) and saturated sodium chloride solution (100 mL) were added. The crude product was extracted with ethyl acetate (3 × 100 mL) and the combined organic layers were dried with sodium sulfate. The solvent was removed under reduced pressure to yield the crude product as a yellow-brown oil, which was purified by flash column chromatography (eluted with 0-25% ethyl acetate in hexane) to give the pure product as a white solid (0.906 g, 3.06 mmol, 20.4% yield, R_f: 0.30 (5:1 (v/v) hexane:ethyl acetate)) and recovered starting material as a white solid (0.785 g, 2.76 mmol, 18.4% yield). Title product: ^{1}H NMR (400 MHz, $CDCl_3$): δ 2.34 (3H, s), 3.52 (2H, s), 3.86 (3H, s), 5.12 (2H, s), 5.64 (1H, t, *J* = 1.3 Hz), 6.07 (1H, s), 6.64 (1H, dd, *J* = 2.0, 8.1 Hz), 6.72 (1H, d, *J* = 2.0 Hz), 6.80 (1H, d, *J* = 8.2 Hz), 7.36 (5H, m) ppm. ^{13}C NMR (101 MHz, $CDCl_3$): δ 26.2, 36.5, 56.1, 71.3, 113.2, 114.3, 121.2, 126.4, 127.4, 127.9, 128.6, 132.4, 137.5, 146.8, 148.9, 149.7, 199.5 ppm. GC-MS (EI) *m/z* (% of base peak): 296 ($M^{\bullet+}$, 19.7), 205 ($M^{\bullet+}$-Bn, 25.8), 187 (7.6), 173 (9.5), 155 (9.9), 145 (15.0), 131 (14.2), 115 (3.2), 103 (5.7), 91 ($Bn^{\bullet+}$, 100.0), 77 (3.7), 65 (12.9). Although no literature spectral data were available, experimental spectral data were consistent with that expected for the title compound.

4-(4-Hydroxy-3-methoxyphenyl)-3-methyl-2-butanone (37)

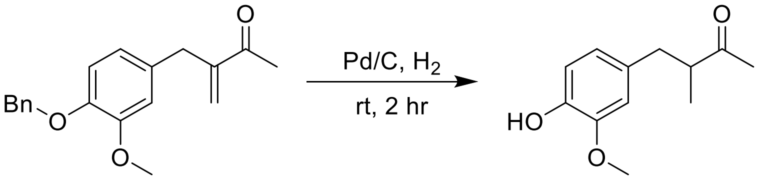

Synthesised using General Method L on a 3.06 mmol scale with a reaction time of 2 hours. Flash column chromatography was performed with 0-40% ethyl acetate in hexane. Off-white solid (0.537 g, 2.58 mmol, 84.4% yield, mp 49-50 °C, R_f: 0.24 (4:1 (v/v) hexane:ethyl acetate), elemental analysis C: 69.22% H: 7.79% (calc. C: 69.21% H: 7.74%)). ^{1}H NMR (400 MHz, $CDCl_3$): δ 1.09 (3H, d, J = 6.9 Hz), 2.08 (3H, s), 2.50 (1H, m), 2.79 (1H, m), 2.91 (1H, m), 3.87 (3H, s), 5.49 (1H, br s), 6.64 (2H, m), 6.82 (1H, d, J = 8.5 Hz) ppm. ^{13}C NMR (101 MHz, $CDCl_3$): δ 16.3, 29.0, 38.7, 49.1, 55.9, 111.5, 114.3, 121.6, 131.6, 144.0, 146.4, 212.5 ppm. GC-MS (EI) *m/z* (% of base peak): 208 ($M^{\bullet+}$, 18.2), 137 ($M^{\bullet+}$-$CH(CH_3)COCH_3$, 100.0), 122 (15.6), 105 (17.7), 94 (17.8), 79 (16.7), 77 (37.2), 65 (22.7), 55 (24.8), 53 (18.6), 52 (15.4), 51 (30.3). Although no literature spectral data were available, experimental spectral data were consistent with that expected for the title compound.

4-(4-Benzyloxy-3-methoxyphenyl)but-3-en-2-one

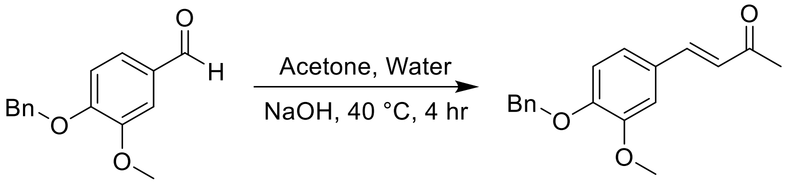

Synthesised using General Method D on a 50.0 mmol scale with 4-benzyloxy-3-methoxybenzaldehyde (12.1 g, 50.0 mmol, 1.0 eq). The crude product was extracted with ethyl acetate (3 × 150 mL) and was purified by recrystallisation from aqueous ethanol. Yellow needles (13.0 g, 46.1 mmol, 92.1% yield, mp 96-97 °C (lit. mp 93 °C[39]), R_f: 0.19 (5:1 (v/v) hexane:ethyl acetate)). ^{1}H NMR (400 MHz, $CDCl_3$): δ 2.36 (3H, s), 3.92 (3H, s), 5.19 (2H, s), 6.59 (1H, d, J = 16.2 Hz), 6.88 (1H, d, J = 8.3 Hz), 7.05 (1H, dd, J = 2.0, 8.3 Hz), 7.09 (1H, d, J = 2.0 Hz), 7.38 (6H, m) ppm. ^{13}C NMR (101 MHz, $CDCl_3$): δ 27.5, 56.1, 71.0, 110.3, 113.6, 122.9, 125.5, 127.3, 127.8, 128.2, 128.8, 136.6, 143.6, 149.9, 150.6, 198.5 ppm. Experimental spectral data were consistent with literature data[40].

4-(4-Benzyloxy-3-methoxyphenyl)-2-pentanone

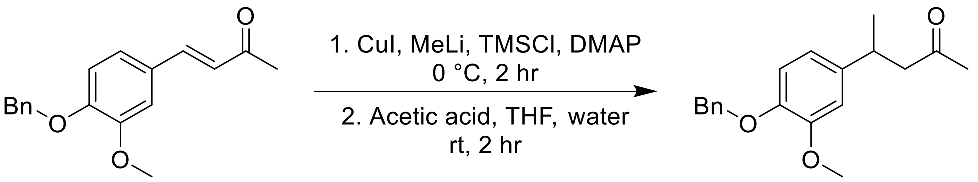

A two neck round bottom flask containing copper(I) iodide (17.1 g, 89.8 mmol, 3.0 eq) was evacuated and filled with argon gas. Dry THF (150 mL) was added and the grey suspension was cooled to 0 °C. Methyllithium solution (1.6 mol L^{-1} in diethyl ether) (113 mL, 181 mmol, 6.0 eq) was slowly added to the grey suspension by cannula transfer. A solution of 4-(4-benzyloxy-3-methoxyphenyl)but-3-en-2-one (8.47 g, 30.0 mmol, 1.0 eq) and trimethylsilyl chloride (7.6 mL, 60. mmol, 2.0 eq) in dry THF (60 mL) was slowly added to

the colourless solution at 0 °C followed by a solution of DMAP (1.83 g, 15.0 mmol, 0.5 eq) in dry THF (30 mL). The yellow solution was stirred at 0 °C for 2 hours and then quenched with a saturated solution of ammonium chloride (100 mL). The crude product was extracted with ethyl acetate (3 × 150 mL) and the combined organic layers were dried with sodium sulfate. The solvent was removed under reduced pressure to yield a pale blue oil. To a solution of the pale blue oil in THF (90 mL) and water (30 mL), was added acetic acid (10.3 mL, 180 mmol, 6.0 eq). The green solution was stirred at room temperature for 2 hours. Sodium hydrogen carbonate solution (50 mL, 10% (w/v)) was added and the crude product was extracted with ethyl acetate (3 × 80 mL). The combined organic layers were dried with sodium sulfate and the solvent was removed under reduced pressure to yield the crude product as a yellow solid, which was purified by flash column chromatography (eluted with 0-25% ethyl acetate in hexane) to give the pure product as a white solid (6.20 g, 20.8 mmol, 69.3% yield, mp 56-57 °C, R_f: 0.25 (4:1 (v/v) hexane:ethyl acetate), elemental analysis C: 76.45% H: 7.35% (calc. C: 76.48% H: 7.43%)). ^{1}H NMR (400 MHz, $CDCl_3$): δ 1.24 (3H, d, *J* = 6.9 Hz), 2.06 (3H, s), 2.61 (1H, dd, *J* = 7.8, 16.1 Hz), 2.72 (1H, dd, *J* = 6.70, 16.0 Hz), 3.24 (1H, m), 3.88 (3H, s), 5.12 (2H, s), 6.68 (1H, dd, *J* = 2.1, 8.2 Hz), 6.75 (1H, d, *J* = 2.0 Hz), 6.81 (1H, d, *J* = 8.2 Hz), 7.36 (5H, m) ppm. ^{13}C NMR (101 MHz, $CDCl_3$): δ 22.2, 30.8, 35.3, 52.4, 56.2, 71.3, 111.1, 114.3, 118.5, 127.4, 127.9, 128.6, 137.5, 139.6, 146.8, 149.7, 208.2 ppm. GC-MS (EI) *m/z* (% of base peak): 298 ($M^{\bullet+}$, 46.4), 241 ($M^{\bullet+}$-CH_2COCH_3, 22.5), 240 (20.6), 207 (21.1), 175 (17.7), 165 (21.2), 149 (4.9), 147 (21.4), 133 (13.2), 105 (7.8), 91 ($Bn^{\bullet+}$, 100.0), 65 (7.2). Although no literature spectral data were available, experimental spectral data were consistent with that expected for the title compound.

4-(4-Hydroxy-3-methoxyphenyl)-2-pentanone (38)

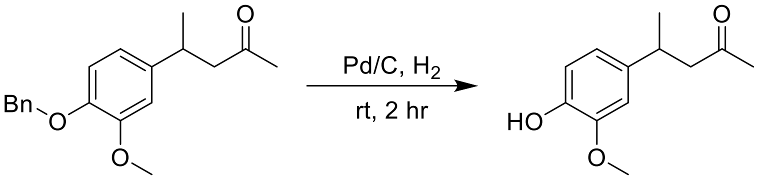

Synthesised using General Method L except the reaction time was 2 hours and flash column chromatography was performed with 0-40% ethyl acetate in hexane. White solid (1.84 g, 8.84 mmol, 88.4% yield, mp 39-42 °C, R_f: 0.16 (4:1 (v/v) hexane:ethyl acetate), elemental analysis C: 69.19% H: 7.64% (calc. C: 69.21% H: 7.74%)). ^{1}H NMR (400 MHz, $CDCl_3$): δ 1.24 (3H, d, *J* = 7.0 Hz), 2.06 (3H, s), 2.62 (1H, dd, *J* = 7.7, 16.1 Hz), 2.72 (1H, dd, *J* = 6.7, 16.1 Hz), 3.24 (1H, m), 3.88 (3H, s), 5.48 (1H, s), 6.70 (2H, m), 6.84 (1H, d, *J* = 8.6 Hz) ppm. ^{13}C NMR (101 MHz, $CDCl_3$): δ 22.4, 30.7, 35.4, 52.4, 56.0, 109.8, 114.5, 119.1, 138.3, 144.1, 146.5, 208.3 ppm. GC-MS (EI) *m/z* (% of base peak): 208 ($M^{\bullet+}$, 35.6), 165 ($M^{\bullet+}$-$COCH_3$, 5.6), 151 ($M^{\bullet+}$-CH_2COCH_3, 100.0), 137 (3.3), 136 (4.0), 135 (2.7), 133 (6.3), 119 (19.3), 105 (5.0), 91 (17.2), 79 (3.1), 77 (4.5). Although no literature spectral data were available, experimental spectral data were consistent with that expected for the title compound.

Field Trials

Supplementary Table S1. Male *B. jarvisi* caught per trap per day (mean ± SE) for the field trials conducted in the years 2017-2019. Means in the same column that have a common letter are not significantly different ($p > 0.05$). Statistical analysis was performed on square root transformed values. A pairwise comparison between each compound was performed separately for each year with Tukey's Honest Significant Difference test for multiple comparisons.

**2017**		**2018**		**2019**	
**Compound**	***B. jarvisi* per Trap per Day**	**Compound**	***B. jarvisi* per Trap per Day**	**Compound**	***B. jarvisi* per Trap per Day**
**1**	27.5 ± 4.0 **a**	**1**	74 ± 17 **a**	**1**	22.9 ± 4.9 **a**
**2**	14.9 ± 2.0 **bc**	**6**	3.8 ± 1.4 **b**	**13**	0.95 ± 0.36 **b**
**3**	5.9 ± 1.1 **d**	**7**	3.87 ± 0.88 **b**	**14**	0.018 ± 0.018 **b**
**4**	2.73 ± 0.64 **e**	**12**	0.200 ± 0.088 **b**	**15**	0.0 ± 0.0 **b**
**5**	13.2 ± 2.5 **b**	**25**	54 ± 11 **a**	**16**	0.0 ± 0.0 **b**
**8**	0.020 ± 0.020 **f**	**34**	1.80 ± 0.57 **b**	**17**	0.0 ± 0.0 **b**
**9**	0.0 ± 0.0 **f**	**35**	0.0 ± 0.0 **b**	**18**	0.035 ± 0.025 **b**
**10**	0.0 ± 0.0 **f**	**36**	0.0 ± 0.0 **b**	**19**	0.0 ± 0.0 **b**
**11**	0.0 ± 0.0 **f**	**39**	0.0 ± 0.0 **b**	**21**	0.0 ± 0.0 **b**
**20**	0.0 ± 0.0 **f**			**22**	0.0 ± 0.0 **b**
**24**	20.7 ± 4.1 **c**			**23**	0.0 ± 0.0 **b**
**29**	0.0 ± 0.0 **f**			**26**	0.263 ± 0.095 **b**
**30**	0.176 ± 0.073 **f**			**27**	21.3 ± 5.1 **a**
**31**	0.098 ± 0.051 **f**			**28**	0.0 ± 0.0 **b**
**32**	0.020 ± 0.020 **f**			**33**	0.263 ± 0.081 **b**
**39**	0.0 ± 0.0 **f**			**37**	8.5 ± 1.7 **c**
				**38**	1.02 ± 0.32 **b**
				**39**	0.05 ± 0.03 **b**

Vapour Pressure

Supplementary Table S2. Temperature-pressure data for compounds **1-3**, **8**, **10-11**, and **24** derived from DSC data with the calculated pressure from the fitted Antoine Equation (Supplementary Table S3) and the percentage difference between the experiment and calculated pressures.

Compound	T / K	P_{exp} / kPa	P_{calc} / kPa	Percentage Difference [a]
**1**	522.6	14.7	14.6	1.30
	503.5	7.73	7.97	-3.03
	483.6	4.03	3.99	0.82
	465.7	2.07	2.01	2.90
	450.8	1.08	1.08	-0.67
	436.8	0.559	0.574	-2.56
	422.5	0.290	0.285	1.60
	410.4	0.149	0.150	-0.42
**2**[b]	498.6	4.00	4.06	-1.48
	480.8	2.09	2.05	1.93
	465.0	1.07	1.06	1.06
	450.5	0.546	0.554	-1.48
	436.9	0.286	0.287	-0.19
	423.0	0.141	0.140	0.88
**3**	499.9	4.00	4.06	-1.40
	481.6	2.07	2.02	2.64
	466.4	1.08	1.08	-0.09
	451.7	0.551	0.560	-1.62
	437.9	0.287	0.289	-0.61
	425.1	0.151	0.150	0.68
**8**	475.9	3.96	4.01	-1.30
	459.7	2.07	2.05	0.86
	445.3	1.08	1.08	0.17
	431.1	0.559	0.545	2.60
	419.2	0.289	0.297	-2.57
	406.7	0.151	0.151	0.10
**10**	503.1	15.2	14.8	2.24
	483.7	7.64	7.85	-2.76
	464.9	4.00	4.00	0.01
	447.4	2.08	2.02	2.84
	433.1	1.07	1.10	-2.45
	417.3	0.543	0.532	2.10
	405.0	0.288	0.290	-0.55
	392.1	0.147	0.145	1.39

**11**	472.5	14.7	14.6	0.74
	453.0	7.55	7.68	-1.80
	435.7	4.07	4.07	-0.07
	418.2	2.06	2.01	2.59
	404.2	1.07	1.08	-0.70
	390.4	0.553	0.554	-0.12
	378.6	0.293	0.298	-1.64
	366.0	0.147	0.146	1.01
**24**	492.8	15.6	15.3	1.61
	472.6	7.55	7.85	-3.84
	453.3	3.94	3.85	2.43
	437.9	2.07	2.05	1.07
	423.6	1.07	1.09	-1.51
	409.7	0.555	0.554	0.12
	396.9	0.282	0.280	0.56
	386.0	0.150	0.150	-0.28

[a] Percentage difference = 100 × (P_{exp} - P_{calc}) / P_{calc}

[b] Impure sample (contains approximately 5% zingerone).

Supplementary Table S3. Antoine Equation parameters A, B, and C and validity range for compounds **1-3**, **8**, **10-11**, and **24** derived from DSC data.

Compound	A	B (K)	C (K)	Validity Range (K)
**1**	6.842163	2457.631516	-89.818662	522.6 to 410.4
**2**	7.731833	3158.757615	-55.111380	498.6 to 423.0
**3**	7.755923	3202.359988	-51.818821	499.9 to 425.1
**8**	8.347736	3444.697922	-31.037649	475.9 to 406.7
**10**	7.193963	2672.791488	-59.369448	503.1 to 392.1
**11**	6.518348	2093.708494	-81.330456	472.5 to 366.0
**24**	6.540607	2096.619364	-101.250770	492.8 to 386.0

References

1. Nomura, H. LXIV.—The pungent principles of ginger. Part I. A new ketone, zingerone (4-hydroxy-3-methoxyphenylethyl methyl ketone) occurring in ginger. *J. Chem. Soc., Trans.* **111**, 769-776 (1917).
2. Agarwal, M., Walia, S., Dhingra, S. & Khambay, B. P. S. Insect growth inhibition, antifeedant and antifungal activity of compounds isolated/derived from *Zingiber officinale* Roscoe (ginger) rhizomes. *Pest Manage. Sci.* **57**, 289-300 (2001).
3. Píšová, M., Pospíšek, J. & Souček, M. Reaction of 4-alkylidene-2, 5-cyclohexadienones with triethylammonium acetate in acetic acid. *Collect. Czech. Chem. Commun.* **40**, 1768-1774 (1975).
4. Allevi, P., Ciuffreda, P., Longo, A. & Anastasia, M. Lipase-catalysed chemoselective monoacetylation of hydroxyalkylphenols and chemoselective removal of a single acetyl group from their diacetates. *Tetrahedron: Asymmetry* **9**, 2915-2924 (1998).
5. Barbero, G. F. *et al.* Application of Hansch's model to capsaicinoids and capsinoids: a study using the quantitative structure− activity relationship. A novel method for the synthesis of capsinoids. *J. Agric. Food Chem.* **58**, 3342-3349 (2010).
6. Poli, G. & Giambastiani, G. An epiisopicropodophyllin aza analogue via palladium-catalyzed pseudo-domino cyclization. *J. Org. Chem.* **67**, 9456-9459 (2002).
7. Iinuma, M., Moriyama, K. & Togo, H. Various oxidative reactions with novel ion-supported (diacetoxyiodo) benzenes. *Tetrahedron* **69**, 2961-2970 (2013).
8. Lapworth, A. & Wykes, F. H. LXVI.—The pungent principles of ginger. Part II. Synthetic preparations of zingerone, methylzingerone and some related acids. *J. Chem. Soc., Trans.* **111**, 790-798 (1917).
9. Dhuru, S. *et al.* Novel diarylheptanoids as inhibitors of TNF-α production. *Bioorg. Med. Chem. Lett.* **21**, 3784-3787 (2011).
10. Pandey, G., Karthikeyan, M. & Murugan, A. New intramolecular α-arylation strategy of ketones by the reaction of silyl enol ethers to photosensitized electron transfer generated arene radical cations: construction of benzannulated and benzospiroannulated compounds. *J. Org. Chem.* **63**, 2867-2872 (1998).
11. Denniff, P., Macleod, I. & Whiting, D. A. Syntheses of the (±)-[n]-gingerols (pungent principles of ginger) and related compounds through regioselective aldol condensations: relative pungency assays. *J. Chem. Soc., Perkin Trans. 1*, 82-87 (1981).
12. Mannich, C. & Merz, K. W. Phenolic bases derived from 1-phenyl-3-aminobutane. *Arch. Pharm. Ber. Dtsch. Pharm. Ges.* **265**, 15-26 (1927).
13. McQuillin, F. J. & Ord, W. O. Mechanisms of catalytic hydrogenation. Part I. An examination of the role of alkali and of acid. *J. Chem. Soc.*, 2902-2908 (1959).
14. Chen, Y.-L. & Barthel, W. F. Some new pyrethrin-type esters. *J. Am. Chem. Soc.* **75**, 4287-4289 (1953).
15. Berlin, A. Y. & Sycheva, T. P. Synthesis of some analogs of zingerone. V. Derivatives of resorcinol. *Zh. Obshch. Khim.* **22**, 1998-2003 (1952).
16. Pinney, K. G. *et al.* Synthesis and biological evaluation of aryl azide derivatives of combretastatin A-4 as molecular probes for tubulin. *Biorg. Med. Chem.* **8**, 2417-2425 (2000).
17. Julia, M. & Chastrette, F. Sur quelques acides salicyliques portant en-4 une chaine acide aliphatique. *Bull. Soc. Chim. Fr.*, 2255-2261 (1962).
18. Zhao, Z. *et al.* Discovery of a tetrahydrobenzisoxazole series of γ-secretase modulators. *ACS Med. Chem. Lett.* **8**, 1002-1006 (2017).
19. Chern, J. *et al.* Affinity-driven covalent modulator of the glyceraldehyde-3-phosphate dehydrogenase (GAPDH) cascade. *Angew. Chem. Int. Ed.* **57**, 7040-7045 (2018).

20. Berlin, A. Y., Sherlin, S. & Serebrennikova, T. Proizvodnye tsingerona. 3. *Zh. Obshch. Khim.* **19**, 759-768 (1949).
21. Winter, M. Odeur et constitution XIX. Sur des homologues et analogues de la p-hydroxyphényl-1-butanone-3 («cétone de framboise»). *Helv. Chim. Acta* **44**, 2110-2121 (1961).
22. Lancefield, C. S., Ojo, O. S., Tran, F. & Westwood, N. J. Isolation of functionalized phenolic monomers through selective oxidation and C-O bond cleavage of the β-O-4 linkages in lignin. *Angew. Chem. Int. Ed.* **54**, 258-262 (2015).
23. Otto, N., Ferenc, D. & Opatz, T. A modular access to (±)-tubocurine and (±)-curine-formal total synthesis of tubocurarine. *J. Org. Chem.* **82**, 1205-1217 (2017).
24. Qin, H., Xu, Z., Cui, Y. & Jia, Y. Total synthesis of (±)-decursivine and (±)-serotobenine: a witkop photocyclization/elimination/*O*-Michael addition cascade approach. *Angew. Chem. Int. Ed.* **50**, 4447-4449 (2011).
25. Ploypradith, P., Cheryklin, P., Niyomtham, N., Bertoni, D. R. & Ruchirawat, S. Solid-supported acids as mild and versatile reagents for the deprotection of aromatic ethers. *Org. Lett.* **9**, 2637-2640 (2007).
26. Nomura, H. & Hotta, S. Synthesis of the homologs of zingerone. *The Science Reports of the Tohoku Imperial University* **14**, 131-142 (1925).
27. Locksley, H., Rainey, D. & Rohan, T. Pungent compounds. Part I. An improved synthesis of the paradols (alkyl 4-hydroxy-3-methoxyphenethyl ketones) and an assessment of their pungency. *J. Chem. Soc., Perkin Trans. 1*, 3001-3006 (1972).
28. Plourde, G. L. 1-(4-Hydroxy-3-methoxyphenyl)-4-methyl-3-pentanone. *Molbank* **2003**, M317 (2003).
29. Kovalenko, V. & Pratsko, A. Selective hydrogenation of conjugated unsaturated ketones containing a hydroxyaryl substituent in the β-position. *Russ. J. Org. Chem.* **53**, 24-28 (2017).
30. Plourde, G. L. 1-(4-Hydroxy-3-methoxyphenyl)-4,4-dimethyl-3-pentanone. *Molbank* **2003**, M320 (2003).
31. Li, P. *et al.* Evolution of the total syntheses of ustiloxin natural products and their analogues. *J. Am. Chem. Soc.* **130**, 2351-2364 (2008).
32. Baranovsky, A., Schmitt, B., Fowler, D. J. & Schneider, B. Synthesis of new biosynthetically important diarylheptanoids and their oxa-and fluoro-analogues by three different strategies. *Synth. Commun.* **33**, 1019-1045 (2003).
33. Das, B., Takhi, M., Kumar, H. S., Srinivas, K. & Yadav, J. Stereochemistry of 4-aryl-2-butanols from Himalayan *Taxus baccata*. *Phytochemistry* **33**, 697-699 (1993).
34. Lalwani, K. G. & Sudalai, A. First enantioselective synthesis of surinamensinol B and a non-natural polysphorin analogue by a two-stereocentered hydrolytic kinetic resolution. *Eur. J. Org. Chem.* **2015**, 7344-7351 (2015).
35. Maiorana, A. *et al.* Structure property relationships of biobased n-alkyl bisferulate epoxy resins. *Green Chem.* **18**, 4961-4973 (2016).
36. Yadav, J., Singh, V. K., Thirupathaiah, B. & Reddy, A. B. First total synthesis and reassignment of absolute configuration of diosniponol A and B. *Tetrahedron Lett.* **55**, 4427-4429 (2014).
37. Luo, D. *et al.* Novel multifunctional dopamine D_2/D_3 receptors agonists with potential neuroprotection and anti-alpha synuclein protein aggregation properties. *Biorg. Med. Chem.* **24**, 5088-5102 (2016).
38. Enders, D., Eichenauer, H. & Pieter, R. Enantioselektive Synthese von (-)-(R)-und (+)-(S)-[6]-Gingerol-Gewürzprinzip des Ingwers. *Chem. Ber.* **112**, 3703-3714 (1979).
39. Dickinson, R., Heilbron, I. M. & Irving, F. CCXLIII.—The intermolecular condensation of styryl methyl ketones. Part I. *J. Chem. Soc.*, 1888-1897 (1927).

40. Mal, K., Sharma, A., Maulik, P. R. & Das, I. PPh_3·HBr-DMSO mediated expedient synthesis of γ-substituted β,γ-unsaturated α-ketomethylthioesters and α-bromo enals: application to the synthesis of 2-methylsulfanyl-3(2 H)-furanones. *Chem. Eur. J.* **20**, 662-667 (2014).
